# Bifunctional organocatalysts for the asymmetric synthesis of axially chiral benzamides

**DOI:** 10.3762/bjoc.13.151

**Published:** 2017-08-02

**Authors:** Ryota Miyaji, Yuuki Wada, Akira Matsumoto, Keisuke Asano, Seijiro Matsubara

**Affiliations:** 1 Department of Material Chemistry, Graduate School of Engineering, Kyoto University, Kyotodaigaku-Katsura, Nishikyo, Kyoto 615-8510, Japan

**Keywords:** axial chirality, benzamide, bifunctional organocatalyst, molecular conformation, multipoint recognition

## Abstract

Bifunctional organocatalysts bearing amino and urea functional groups in a chiral molecular skeleton were applied to the enantioselective synthesis of axially chiral benzamides via aromatic electrophilic bromination. The results demonstrate the versatility of bifunctional organocatalysts for the enantioselective construction of axially chiral compounds. Moderate to good enantioselectivities were afforded with a range of benzamide substrates. Mechanistic investigations were also carried out.

## Introduction

Bifunctional organocatalysts have significantly contributed to the field of asymmetric synthesis [1–6]. In these catalysts, (thio)urea and tertiary amino functional groups cooperatively activate a nucleophile and an electrophile simultaneously, in a suitable spatial configuration. Thus, they enable various stereoselective addition reactions to occur. Organocatalysts have also been employed in several asymmetric cyclization reactions via intramolecular hetero-Michael addition [7–16]. In these reactions, multipoint recognition by the catalysts favors the specific conformations of the substrates in the transition state. Several successful results and a recent trend in organocatalytic atroposelective reactions, including enantioselective formation of chiral axes [17–24], dynamic kinetic resolution [25–41], kinetic resolution [42–47], desymmetrization [48–54], de novo annulation [55–61], and point-to-axial chirality transfer [58–59] (for reviews, see references [31,62–63]), motivated us to expand on the utility of this class of small-molecule catalysts. We have recently demonstrated that bifunctional organocatalysts can also be applied to the asymmetric synthesis of axially chiral compounds (biaryls bearing isoquinoline *N*-oxides or quinolines and phenolic moieties) by translating a specific conformation, recognized by bifunctional organocatalysts, into axial chirality [36–37]. Thus, we assumed that this method could be further applied to the enantioselective synthesis of a range of axially chiral compounds. In this study, we present the enantioselective synthesis of 3-hydroxybenzamides via aromatic electrophilic bromination [28–29]. The 3-hydroxybenzamide substrates comprise both amide and phenolic moieties. These can interact with a hydrogen-bond donor and a hydrogen-bond acceptor, respectively. Such interactions are expected to recognize a specific conformation of the substrate molecule to realize the enantioselective construction of axially chiral benzamides [36–37].

## Results and Discussion

**Figure 1:**
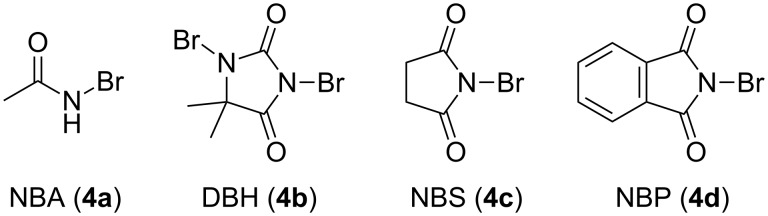
Brominating reagents.

We initiated our investigations using 3-hydroxy-*N*,*N*-diisopropylbenzamide (**1a**) and *N*-bromoacetamide (NBA, **4a**) as a brominating reagent, with 10 mol % quinidine-derived bifunctional catalyst **3a**, in toluene, at −40 °C. As expected, the tribrominated product **2a** was formed enantioselectively (Table 1, entry 1). Although a lower temperature did not improve the enantioselectivity (Table 1, entry 2), lowering the concentration of the reaction mixture was effective (Table 1, entry 3). The screening of solvents identified ethyl acetate as the most suitable solvent (Table 1, entries 4–7). Other brominating reagents (Figure 1) were also investigated; however, NBA (**4a**) still afforded the best enantioselective results (Table 1, entries 8–10). In addition, other bifunctional organocatalysts derived from easily available cinchona alkaloids exhibited similarly good enantioselectivities; **3c** and **3d** afforded the opposite enantiomer of the product (Table 1, entries 11–13, results of further catalyst screening are described in the Supporting Information File 1).

**Table 1:** Optimization of conditions.^a^

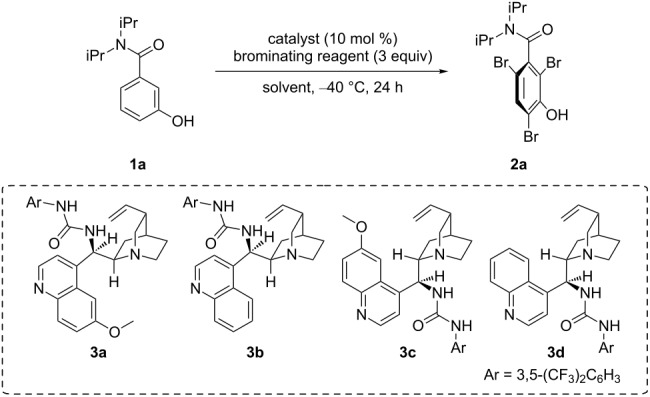					

Entry	Catalyst	Brominating reagent	Solvent	Yield (%)^b^	ee (%)

1^c^	**3a**	NBA (**4a**)	toluene	88	78
2^c,d^	**3a**	NBA (**4a**)	toluene	48	78
3	**3a**	NBA (**4a**)	toluene	58	84
4	**3a**	NBA (**4a**)	CHCl_3_	73	84
5	**3a**	NBA (**4a**)	Et_2_O	66	42
6	**3a**	NBA (**4a**)	THF	69	82
7	**3a**	NBA (**4a**)	EtOAc	84	87
8^e^	**3a**	DBH (**4b**)	EtOAc	99	77
9	**3a**	NBS (**4c**)	EtOAc	99	51
10	**3a**	NBP (**4d**)	EtOAc	99	72
11	**3b**	NBA (**4a**)	EtOAc	56	84
12	**3c**	NBA (**4a**)	EtOAc	89	−81
13	**3d**	NBA (**4a**)	EtOAc	76	−80

^a^Reactions were run using **1a** (0.1 mmol), the catalyst (0.01 mmol), and the brominating reagent (0.3 mmol) in the solvent (10 mL). ^b^Isolated yields. ^c^Reactions were run in 0.5 mL of toluene. ^d^Reaction was run at −45 °C. ^e^1.5 equiv of **4b** was used for the reaction.

**Scheme 1:**
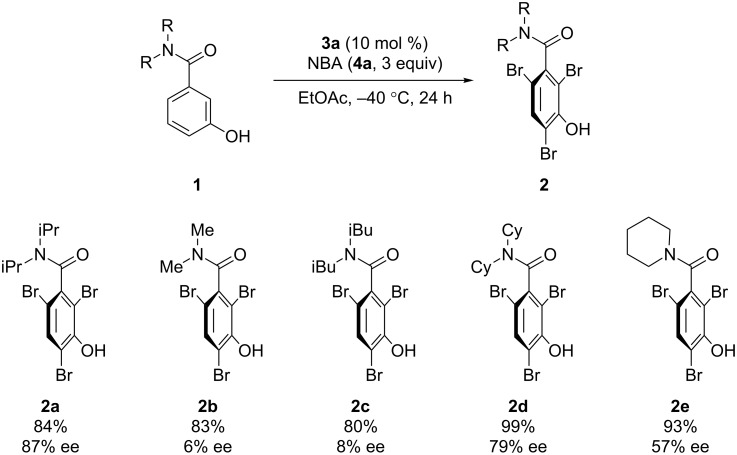
Optimization of the substituents of the amide group. Reactions were run using **1** (0.1 mmol), **3a** (0.01 mmol), and **4a** (0.3 mmol) in EtOAc (10 mL). Yields represent material isolated after silica gel column chromatography.

We then investigated substrates bearing other substituents on the amino group (Scheme 1). Dimethyl- and diisobutylamide groups resulted in much lower enantioselectivities (**2b** and **2c**). Substrates bearing cyclohexyl groups or a piperidinyl moiety provided the corresponding products in high yields; however, the enantioselectivities were not as high as that of **2a**. The absolute configuration of **2d** was determined by X-ray analysis (see the Supporting Information File 1 for details), and the configurations of all other examples were assigned analogously.

**Scheme 2:**
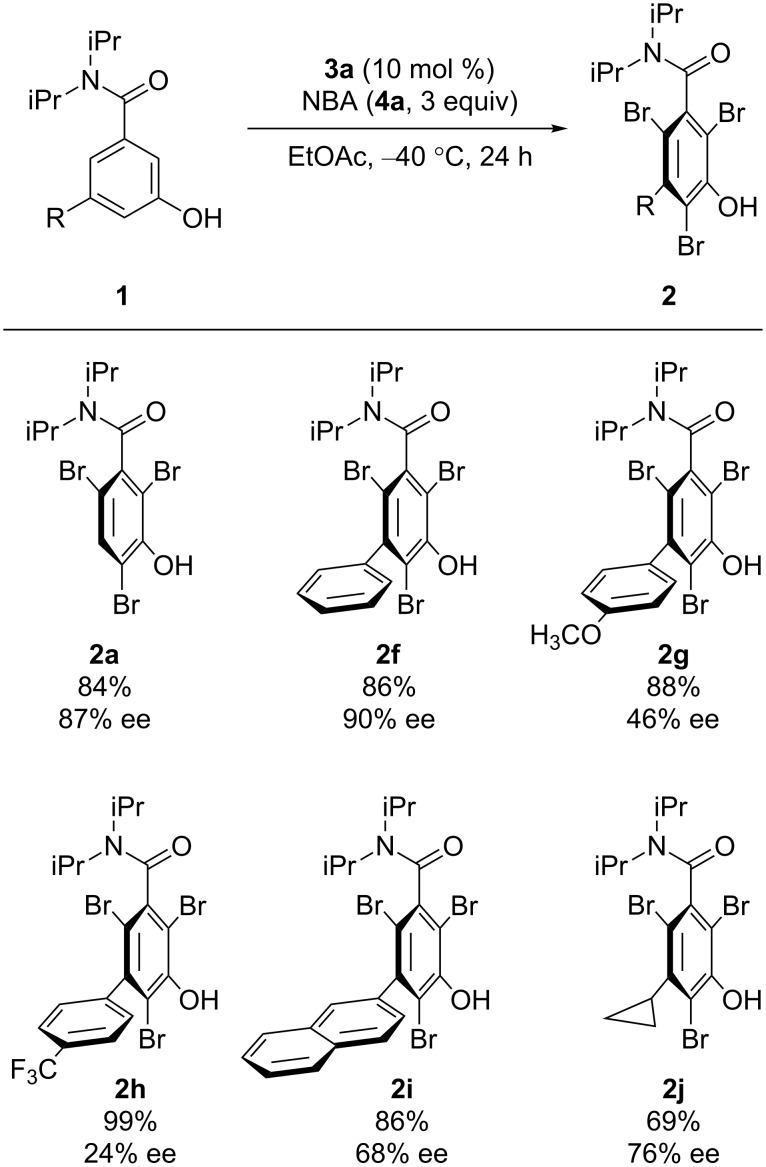
Substrate scope. Reactions were run using **1** (0.1 mmol), **3a** (0.01 mmol), and **4a** (0.3 mmol) in EtOAc (10 mL). Yields represent material isolated after silica gel column chromatography.

**Scheme 3:**
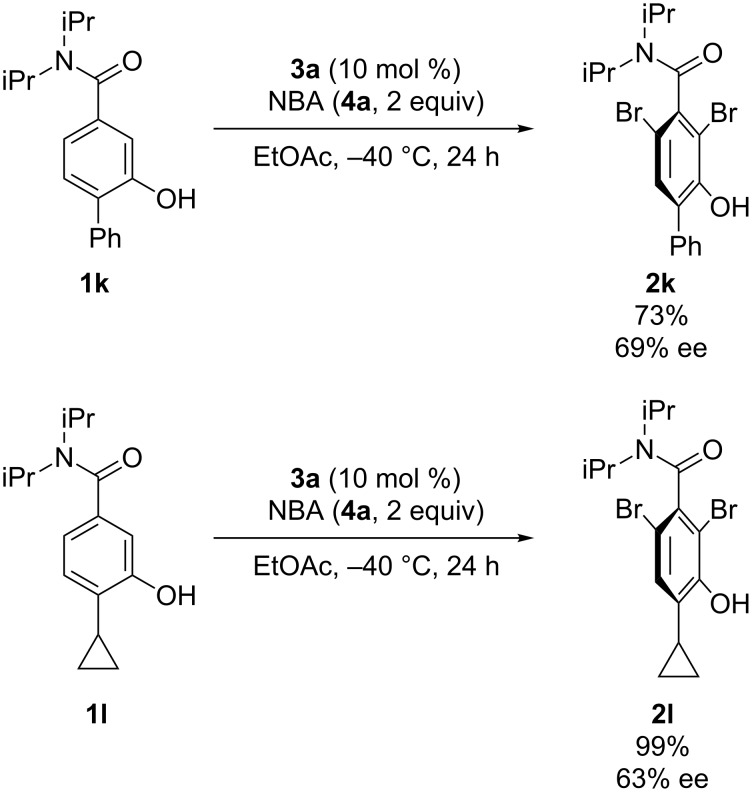
Reactions of substrates with substituted phenols.

Once the optimal conditions for the transformation were established, we next proceeded to explore the substrate scope (Scheme 2). The substrate bearing a phenyl group yielded the product with the highest enantioselectivity (Scheme 2, **2f**). However, a decrease in enantioselectivity was observed when the phenyl group was replaced by substituted phenyl groups (Scheme 2, **2g** and **2h**). The substrate bearing a naphthyl group afforded the corresponding product in moderate enantioselectivity (Scheme 2, **2i**). In addition, a benzamide with a cyclopropyl group also provided the product in good enantioselectivity (Scheme 2, **2j**). Furthermore, when the reaction was carried out using **1k** and **1l** with 2 equiv of NBA (**4a**), dibromination proceeded in high yields and moderate enantioselectivities (Scheme 3); both **1k** and **1l** comprise a substituent ortho to the hydroxy group. These brominated axially chiral benzamides can further be derivatized for the synthesis of functional molecules [64].

**Scheme 4:**
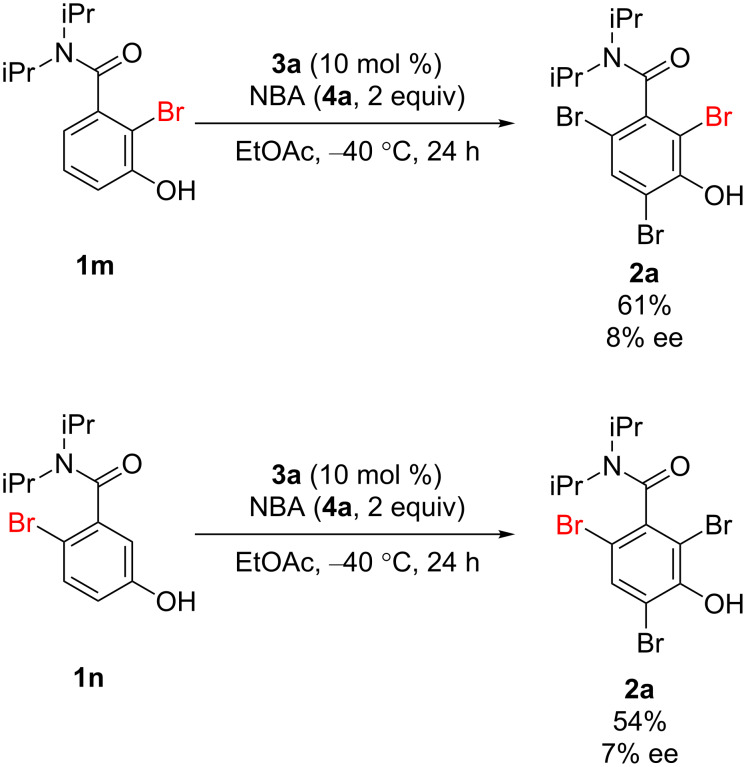
Reactions of monobrominated substrates.

**Scheme 5:**
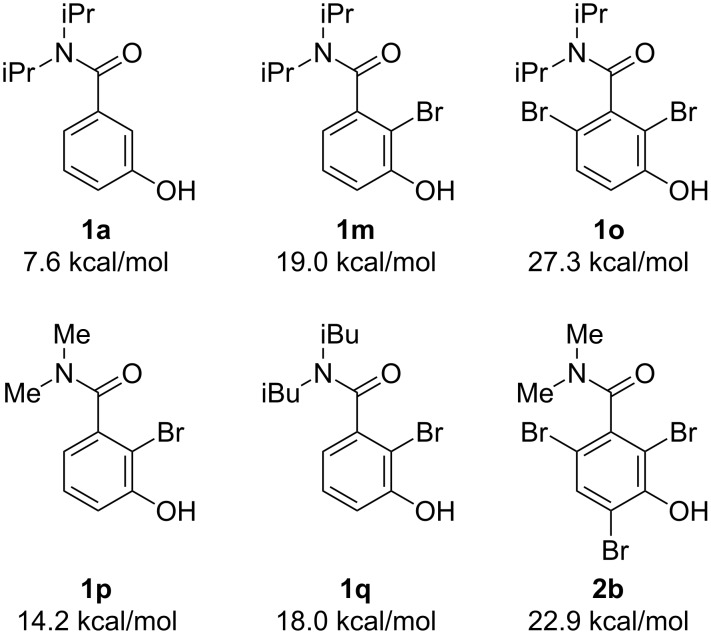
Rotational barriers of substrates and intermediates calculated at the B3YLP/6-31G(d) level of theory.

To gain insight into the reaction mechanism, the reactions were performed using substrates **1m** and **1n**, previously monobrominated at the *ortho*-positions of the rotational axis. Much lower enantioselectivities than that afforded by **1a** were observed in both reactions (Scheme 4). In addition, the reaction was also carried out with 1 equiv of NBA (**4a**). The sole product afforded was **1m** and most of the starting material was recovered (see Supporting Information File 1 for details). These results imply that the first bromination, occurring at the *ortho*-position of the axis (probably at the 2-position), is the enantiodetermining step of the reaction. Moreover, once one of the *ortho*-positions is brominated, racemization through bond rotation is negligible during further brominations [65]. Indeed, the rotational barrier of substrate **1a**, calculated at the B3YLP/6-31G(d) level of theory, is only 7.6 kcal/mol; on the other hand, that of the monobrominated intermediate **1m** is 19.0 kcal/mol (Scheme 5). However, this latter value is not high enough to inhibit bond rotation at room temperature. This explains why the reactions must be carried out at such a low temperature (−40 °C) to afford high enantioselectivities. Compound **1o**, with both *ortho*-positions brominated, has a rotational barrier that is high enough to enable the isolation of the optically active form, even at room temperature. Furthermore, it is also important to employ substrates bearing bulky substituents on the nitrogen atom. Such substrates limit the bond rotation about the chiral axis to realize high enantioselectivity (Scheme 1). The rotational barriers of monobrominated compounds **1p** and **1q** (bearing methyl and isobutyl groups, respectively, on the amide moiety) are lower than that of **1m**. Although racemization of **2b**, the rotational barrier of which is 22.9 kcal/mol, was observed after a lot of months, it is enough slow to enable the immediate analysis of the reaction selectivity (the decrease of the enantiomeric purity of **2b** was negligible after a day).

**Scheme 6:**
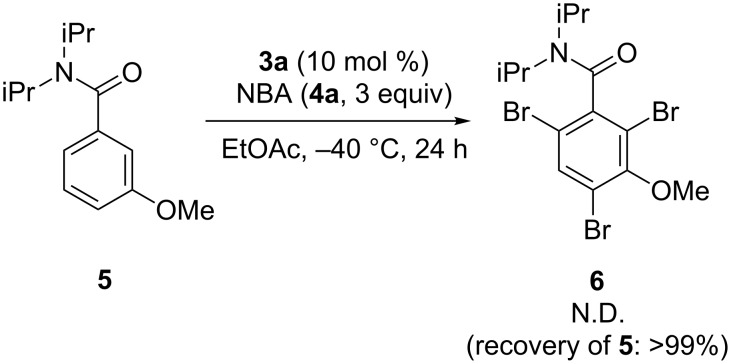
Reaction of substrate with protected phenol.

Furthermore, the reaction of benzamide **5**, bearing a protected phenol, was carried out (Scheme 6). It failed to give the corresponding product **6**, indicating the significance of multipoint activation involving the phenolic hydroxy group.

## Conclusion

In summary, we demonstrated a novel enantioselective synthesis of axially chiral benzamides, using bifunctional organocatalysts, via aromatic electrophilic halogenation. Moderate to good enantioselectiveties were accomplished with various benzamide substrates. These results, along with ones reported in our previous work and other literature [35–38,58–59], verify the utility of bifunctional organocatalysts for application in the synthesis of various axially chiral compounds. Further studies regarding the detailed clarification of the reaction mechanism and application of this method to the construction of other axially chiral structures are currently underway and will be reported in due course.

## Supporting Information

File 1 Experimental procedures, characterization data, copies of the ^1^H, ^13^C NMR spectra, HPLC chromatogram profiles, and the ORTEP drawing.

File 2 Crystallographic information file of compound **2d**.
