# Supplementary material for: Bifunctional organocatalysts for the asymmetric synthesis of axially chiral benzamides
Source: Beilstein J Org Chem. 2017 Aug 2;13:1518–23. doi: 10.3762/bjoc.13.151 (PMC5550815; doi:10.3762/bjoc.13.151)
Supplement: File 1 — Experimental procedures, characterization data, copies of the 1H, 13C NMR spectra, HPLC chromatogram profiles, and the ORTEP drawing. [file Beilstein_J_Org_Chem-13-1518-s001.pdf]

Supporting Information
for

Bifunctional organocatalysts for the asymmetric synthesis of axially chiral benzamides

Ryota Miyaji, Yuuki Wada, Akira Matsumoto, Keisuke Asano* and Seijiro Matsubara*

Address: Department of Material Chemistry, Graduate School of Engineering, Kyoto University, Kyotodaigaku-Katsura, Nishikyo, Kyoto 615-8510, Japan

Email: Keisuke Asano* - asano.keisuke.5w@kyoto-u.ac.jp; Seijiro Matsubara* - matsubara.seijiro.2e@kyoto-u.ac.jp

*Corresponding author

Experimental procedures, characterization data, copies of the ^{1}H, ^{13}C NMR spectra, HPLC chromatogram profiles, and the ORTEP drawing

Contents

Instrumentation and Chemicals S2

Experimental Procedure S3

Table S1 S19

Scheme S1 S21

Characterization Data of Products S22

NMR Spectra (^{1}H, ^{13}C) of Products S30

HPLC Chromatogram Profiles S54

ORTEP Drawing of 2d S66

DFT Calculations of Rotational Barriers of 1a, 1m, 1o, 1p, 1q, 2b and 2e S69

Instrumentation and Chemicals

^{1}H and ^{13}C Nuclear magnetic resonance spectra were taken on a Varian UNITY INOVA 500 (^{1}H, 500 MHz; ^{13}C, 125.7 MHz) spectrometer using tetramethylsilane as an internal standard for ^{1}H NMR (δ = 0 ppm) and $CDCl_3$ as an internal standard for ^{13}C NMR (δ = 77.0 ppm). ^{1}H NMR data are reported as follows: chemical shift, multiplicity (s = singlet, d = doublet, t = triplet, q = quartet, quint = quintet, sext = sextet, sept = septet, br = broad, m = multiplet), coupling constants (Hz), integration. ^{19}F NMR spectra were measured on a Varian Mercury 200 (^{19}F, 188 MHz) spectrometer with hexafluorobenzene as an internal standard (δ = 0 ppm). Mass spectra were recorded on a SHIMADZU GCMS-QP2010 Plus (EI) and a Thermo Scientific Exactive (ESI, APCI) spectrometers. High performance liquid chromatography (HPLC) was performed with a SHIMADZU Prominence. Infrared (IR) spectra were determined on a SHIMADZU IR Affinity-1 spectrometer. Melting points were determined using a YANAKO MP-500D. Optical rotations were measured on a HORIBA SEPA-200. X-ray data were taken on a Rigaku XtaLAB mini diffractometer equipped with a CCD detector. TLC analyses were performed by means of Merck Kieselgel 60 F_{254} (0.25 mm) Plates. Visualization was accomplished with UV light (254 nm) and/or such as an aqueous alkaline $KMnO_4$ solution followed by heating.

Flush column chromatography was carried out using Kanto Chemical silica gel (spherical, 40–50 μm). Unless otherwise noted, commercially available reagents were used without purification.

DFT calculations were performed with Gaussian 09 packages. The DFT method was employed using the B3LYP hybrid functional. Structures were optimized with the 6-31G(d) basis set.

Experimental Procedure

General procedure for asymmetric synthesis of 2,4,6-tribromo-3-hydroxybenzamides 2

To a 20-mL round-bottom flask were sequentially added substrate **1** (0.10 mmol), quinidine-derived bifunctional catalyst **3a** (5.8 mg, 0.010 mmol), and EtOAc (10 mL). The mixture was stirred at –40 °C for 30 min. To the resulting solution was added *N*-bromoacetamide (**4a**, 41 mg, 0.30 mmol). The reaction mixture was then stirred for 24 h. The mixture was quenched with saturated aqueous $Na_2S_2O_3$ (7.0 mL), and then the aqueous phase was extracted with EtOAc (5.0 mL × 2). The combined organic layers were dried over Na_2SO_4 and concentrated in vacuo. Purification of the reaction mixture by flush silica gel column chromatography using hexane/EtOAc (v/v = 1/1) as an eluent afforded the corresponding 2,4,6-tribromo-3-hydroxybenzamides **2**.

Racemic compounds were prepared using DABCO as a catalyst.

General procedure for reactions from substrates 1k and 1l

To a 20-mL round-bottom flask were sequentially added substrate **1** (0.10 mmol), **3a** (5.8 mg, 0.010 mmol), and EtOAc (10 mL). The mixture was stirred at –40 °C for 30 min. To the resulting solution was added **4a** (28 mg, 0.20 mmol). The reaction mixture was then stirred for 24 h. The mixture was quenched with saturated aqueous $Na_2S_2O_3$ (7.0 mL), and then the aqueous phase was extracted with EtOAc (5.0 mL × 2). The combined organic layers were dried over Na_2SO_4 and concentrated in vacuo. Purification of the reaction mixture by flush silica gel column chromatography using hexane/EtOAc (v/v = 1/1) as an eluent afforded the corresponding product **2k** and **2l**.

Racemic compounds were prepared using DABCO as a catalyst.

General procedure for reactions from monobrominated substrates 1m and 1n

To a 20-mL round-bottom flask were sequentially added the monobrominated substrate **1** (30 mg, 0.10 mmol), **3a** (5.8 mg, 0.010 mmol), and EtOAc (10 mL). The mixture was stirred at –40 °C for 30 min. To the resulting solution was added **4a** (28 mg, 0.20 mmol). The reaction mixture was then stirred for 24 h. The mixture was quenched with saturated aqueous $Na_2S_2O_3$ (7.0 mL), and then the aqueous phase was extracted with EtOAc (5.0 mL × 2). The combined organic layers were dried over Na_2SO_4 and concentrated in vacuo. Purification of the reaction mixture by flush silica

gel column chromatography using hexane/EtOAc (v/v = 1/1) as an eluent afforded **2a**.

Racemic compounds were prepared using DABCO as a catalyst.

General procedure for preparation of bifunctional catalysts 3

Bifunctional organocatalysts **3a**–**d** were prepared by the literature procedure.[1] A cinchona alkaloid (5.0 mmol) and triphenylphosphine (1.6 g, 6.0 mmol) were dissolved in THF (25 mL), and the solution was cooled to 0 °C. Diethyl azodicarboxylate (1.0 g, 6.0 mmol) was subsequently added. To the resulting solution was added dropwise the solution of diphenyl phosphoryl azide (1.3 mL, 6.0 mmol) in THF (10 mL) at 0 °C. The mixture was allowed to warm to ambient temperature. After being stirred for 24 h, it was heated to 50 °C and stirred for 10 h. Triphenylphosphine (1.7 g, 6.5 mmol) was added again, and the mixture was stirred at 50 °C for additional 15 h. After the solution was cooled to ambient temperature, H_2O (0.50 mL) was added, and the solution was stirred for 24 h. The solvents were removed in vacuo, and the residue was dissolved in CH_2Cl_2/10% aqueous HCl (25 mL/25 mL). The aqueous phase was separated and washed with CH_2Cl_2 (25 mL × 4). It was subsequently made alkaline with aqueous NH_3, and the aqueous phase was extracted with CH_2Cl_2 (25 mL × 4). The combined organic layers were dried over Na_2SO_4, and concentrated in vacuo. Purification by flush silica gel column chromatography using EtOAc/CH_3OH (v/v = 9/1) then $CHCl_3$/CH_3OH (v/v = 8/2) as an eluent gave the corresponding 9-amino(9-deoxy)cinchona alkaloids. Next, to the solution of the obtained 9-amino(9-deoxy)cinchona alkaloid in THF (6.0 mL) was slowly added a solution of 3,5-bis(trifluoromethyl)phenyl isocyanate (1.0 equiv) in THF (4.0 mL) at ambient temperature. The mixture was stirred overnight, and the solvents were removed in vacuo. Purification by flush silica gel column chromatography using EtOAc/CH_3OH (v/v = 95/5–97.5/2.5) or EtOAc as an eluent gave the corresponding bifunctional organocatalyst **3a**–**d**. The characterization results are as below.

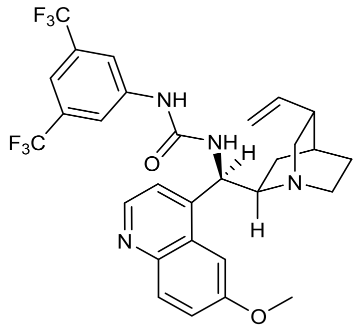

3a. White solid; 30% yield (for 2steps from quinidine). $[\alpha]_D^{18}$ +840.0 (*c* 2.00, CH_2Cl_2). ^{1}H NMR ($CDCl_3$) δ 8.76 (d, *J* = 4.5 Hz, 1H), 8.05 (d, *J* = 9.5 Hz, 1H), 7.78 (s, 2H), 7.60 (s, 1H), 7.41 (m, 3H), 6.29 (br s, 1H), 5.88 (ddd, *J* = 15.0, 10.0, 4.0 Hz, 1H), 5.33 (br s, 1H), 5.13 (m, 2H), 3.99 (s, 3H), 2.97 (d, *J* = 10.0 Hz, 3H), 2.86 (t, *J* = 8.0 Hz, 2H), 2.23 (m, 1H), 1.82 (br s, 3H), 1.68 (br s, 1H), 1.51 (m, 1H), 1.03 (m, 1H). ^{13}C NMR ($CDCl_3$) δ 158.4, 156.9, 156.2, 155.1, 147.7, 145.1, 140.9, 140.1, 132.3 (q, *J* = 33.2 Hz), 132.1, 128.3, 123.4 (q, *J* = 272.6 Hz), 118.5, 115.9, 115.4, 110.0, 101.7, 60.6, 55.8, 49.3, 47.2, 39.1, 32.2, 27.4, 26.6, 25.5. ^{19}F NMR ($CDCl_3$) δ 98.8. Mp. 133.0–133.5 °C. IR (KBr): 3321, 3080, 2941, 2875, 1705, 1676, 1624, 1570, 1511, 1475, 1434, 1389, 1279, 1245, 1229, 1179, 1132, 1096, 1036, 945, 917, 880, 852, 828, 703, 682 cm^{-1}. HRMS Calcd for $C_{29}H_{29}F_6N_4O_2$: $[M+H]^+$, 580.2223. Found: *m*/*z* 580.2209.

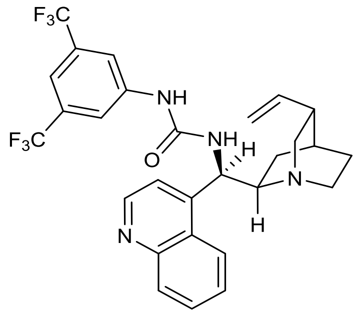

3b. White solid; 40% yield (for 2steps from cinchonine). $[\alpha]_D^{23}$ +194.9 (*c* 0.59, CH_2Cl_2). ^{1}H NMR ($CDCl_3$) δ 8.91 (d, *J* = 4.5 Hz, 1H), 8.36 (d, *J* = 7.5 Hz, 1H), 8.18 (dd, *J* = 8.3, 0.75 Hz, 1H), 7.79 (s, 2H), 7.76 (dd, *J* = 8.3, 1.3, 1H), 7.64 (t, *J* = 7.5 Hz, 1H), 7.48 (d, *J* = 4.5 Hz, 1H), 7.43 (s, 1H), 6.35 (br s, 1H), 5,87 (ddd, *J* = 18.1, 15.0, 6.0 Hz, 1H), 5.30 (br s, 1H), 5.13 (dd, *J* = 24.0, 7.5 Hz, 2H), 2.94 (m, 5H), 2.31 (m, 1H), 1.84 (br s, 1H), 1.65 (br s, 1H), 1.57 (m, 1H), 1.49 (m, 1H), 1.27 (m, 2H). ^{13}C NMR ($CDCl_3$) δ 154.9, 150.1, 148.7, 140.7, 139.6, 132.2 (q, *J* = 33.2 Hz), 130.6, 129.4, 127.0, 123.7 (q, *J* = 273.0 Hz), 123.1, 118.26, 118.23, 115.76, 115.73, 115.70, 115.3, 61.1, 49.0, 47.0, 39.0, 29.7, 27.3, 26.3, 25.0. ^{19}F NMR ($CDCl_3$) δ 98.8. Mp. 193.5–194.0 °C. IR (KBr): 3289, 3238, 3081, 2942, 2875, 2366, 1705, 1676, 1570, 1511, 1475, 1389, 1279, 1243,

1180, 1132, 945, 916, 881, 761, 683, 624 cm^{-1}. HRMS Calcd for $C_{28}H_{27}F_6N_4O$: $[M+H]^+$, 549.2084. Found: *m*/*z* 549.2077.

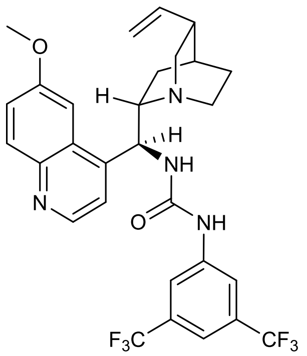

3c. White solid; 30% yield (for 2steps from quinine). $[\alpha]_D^{23}$ +20.4 (*c* 1.47, CH_2Cl_2). ^1H NMR ($CDCl_3$) δ 8.83 (d, *J* = 4.5 Hz, 1H), 8.06 (d, *J* = 9.5 Hz, 1H), 7.94 (br s, 1H), 7.74 (s, 1H), 7.68 (s, 1H), 7.42 (dd, *J* = 9.0, 3.0 Hz, 1H), 7.34 (d, *J* = 4.5 Hz, 1H), 7.32 (s, 1H), 6.13 (br s, 1H), 5.64 (ddd, *J* = 17.0, 10.3, 6.8 Hz, 2H), 5.01 (d, *J* = 10.0 Hz, 1H), 4.84 (d, *J* = 17.0 Hz, 1H), 4.02 (s, 3H), 3.54 (br s, 1H), 3.18 (br s, 1H), 2.95 (m, 1H), 2.71 (m, 1H), 2.24 (br s, 2H), 2.11 (br s, 1H), 1.66 (m, 5H). ^{13}C NMR ($CDCl_3$) δ 158.4, 154.6, 153.7, 147.3, 145.1, 140.5, 140.4, 131.932 (q, *J* = 33.2 Hz), 131.927, 130.2, 123.0 (q, *J* = 273.0 Hz), 118.4, 115.6, 115.1, 112.5, 109.7, 103.9, 60.1, 55.8, 55.4, 43.6, 41.4, 40.7, 38.6, 27.4, 26.9. ^{19}F NMR ($CDCl_3$) δ 98.6. Mp. 134.0–135.0 °C. IR (KBr): 3327, 3083, 2944, 2869, 2360, 1700, 1623, 1570, 1512, 1476, 1388, 1279, 1245, 1230, 1179, 1132, 1034, 881, 852, 682 cm^{-1}. HRMS Calcd for $C_{29}H_{29}F_6N_4O_2$: $[M+H]^+$, 580.2223. Found: *m*/*z* 580.2181.

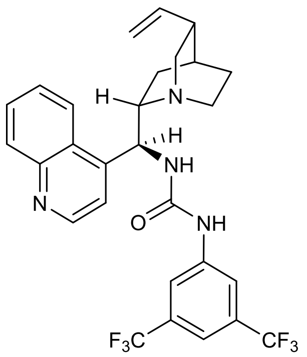

3d. White solid; 40% yield (for 2steps from cinchonidine). $[\alpha]_D^{23}$ –16.3 (*c* 3.67, CH_2Cl_2). ^1H NMR ($CDCl_3$) δ 8.93 (d, *J* = 3.0 Hz, 1H), 8.44 (d, *J* = 8.5 Hz, 1H), 8.17 (dd, *J* = 7.5, 1.3 Hz, 1H), 7.76 (m, 3H), 7.66 (m, 1H), 7.48 (d, *J* = 5.0 Hz, 1H), 7.38 (s, 1H), 6.49 (br s, 1H), 5.61 (ddd, *J* = 17.3, 10.3, 7,5 Hz, 1H), 5.44 (br s, 1H), 4.90 (m, 2H),

3.17 (br s, 1H), 2.99 (dd, *J* = 13.5, 10.0 Hz, 2H), 2.61 (m, 1H), 2.41 (m, 2H), 2.23 (m, 1H), 1.63 (m, 2H), 1.56 (m, 1H), 1.36 (m, 1H), 0.93 (dd, *J* = 13.5, 6.0 Hz, 1H). ^{13}C NMR ($CDCl_3$) δ 154.8, 149.9, 148.6, 148.5, 141.5, 140.8, 140.7, 132.0 (q, *J* = 33.2 Hz), 130.3, 129.6, 127.2, 123.28, 123.11 (q, *J* = 273.0 Hz), 118.2, 115.6, 114.8, 113.0, 61.9, 55.5, 40.9, 39.1, 35.0, 27.6, 27.0, 26.0. ^{19}F NMR ($CDCl_3$) δ 98.7. Mp. 140.0–141.0 °C. IR (KBr): 3309, 3081, 2947, 2869, 2360, 1700, 1623, 1570, 1511, 1473, 1389, 1346, 1279, 1243, 1180, 1132, 882, 760, 704, 683 cm^{-1}. HRMS Calcd for $C_{28}H_{27}F_6N_4O$: $[M+H]^+$, 549.2084. Found: *m*/*z* 549.2076.

General procedure for preparation of substrates 1a–1e

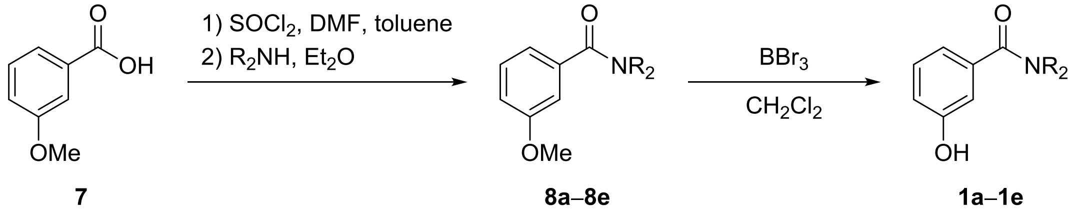

Substrate **1a**–**1e** were prepared by the literature procedere.[2] To a solution of 3-methoxybenzoic acid (**7**, 1.5 g, 10 mmol) in toluene (15 mL) was added thionyl chloride (0.87 mL, 12 mmol) and a catalytic ammount of DMF (0.080 mL, 1.0 mmol). After the resulting mixture was stirred for 30 min, the solvents were removed, and the crude product was dried in vacuo. The product was placed in a 50-mL reaction vessel. To the flask was added CH_2Cl_2 (15 mL) and *N*,*N*-dialkyllamine (20 mmol), and the solution was stirred for 4 h. The mixture was quenched with 1.0 M aqueous HCl (10 mL), and the organic layers were washed with 1.0 M aqueous HCl, brine, and 1.0 M aqueous NaOH, and subsequently dried over Na_2SO_4. Purification by flush silica gel column chromatography using hexane/EtOAc (v/v = 3/1) as an eluent gave *N*,*N*-diisopropyl-3-methoxybenzamide **8** as a white solid in 41–99% yield.

To a solution of **8** (5.0 mmol) in CH_2Cl_2 (15 mL) was added dropwise the solution of BBr_3 (20 mL, ca. 1.0 M in CH_2Cl_2 solution, 20 mmol) at –78 °C. Then the reaction mixture was steadily warmed to ambient temperature. The resulting solution was carefully quenched with H_2O, extracted with Et_2O, washed with brine, dried over Na_2SO_4, and concentrated in vacuo. Recrystallization from hot EtOH/hexane gave the corresponding 3-hydroxybenzamides **1a**–**1e** as a white solid in 58–98% yield.

3-Hydroxy-*N*,*N*-diisopropylbenzamide (1a).

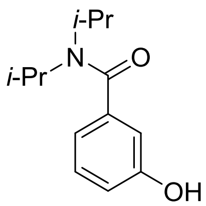

White solid; 95% yield for preparation of **8a** (**5**), 76% yield for preparation of **1a**.
^{1}H NMR ($CDCl_3$) δ 8.79 (s, 1H), 7.09 (dd, *J* = 8.0, 8.0 Hz, 1H), 6.73 (br s, 1H), 6.69–6.66 (m, 2H), 3.91 (sept, *J* = 6.5 Hz, 1H), 3.51 (sept, *J* = 6.5 Hz, 1H), 1.55 (d, *J* = 6.5 Hz, 6H), 1.11 (d, *J* = 6.5 Hz, 6H). ^{13}C NMR ($CDCl_3$) δ 172.2, 157.0, 138.2, 129.4, 116.8, 116.0, 113.7, 51.2, 46.0, 20.6. Mp. 124.0–125.0 °C. TLC: R_f 0.30 (hexane/EtOAc = 1:1). IR (KBr): 3247, 3001, 2971, 2937, 1611, 1580, 1457, 1352, 1231, 1152, 1039, 877, 781,

710 cm^{-1}. HRMS Calcd for $C_{13}H_{20}NO_2$: $[M+H]^+$, 222.1489. Found: *m*/*z* 222.1485.

3-Hydroxy-*N*,*N*-dimethylbenzamide (1b).

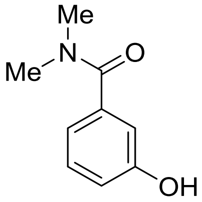

White solid; 44% yield for preparation of **8b**, 78% yield for preparation of **1b**.
^{1}H NMR ($CDCl_3$) δ 8.21 (br s, 1H), 7.18 (dd, *J* = 8.0, 7.5 Hz, 1H), 6.99 (dd, *J* = 1.5, 1.0 Hz, 1H), 6.83 (ddd, *J* = 8.0, 2.0, 1.0 Hz, 1H), 6.82 (ddd, *J* = 7.5, 2.0, 1.5 Hz, 1H), 3.12 (s, 3H), 2.98 (s, 3H). ^{13}C NMR ($CDCl_3$) δ 172.2, 156.9, 136.4, 129.4, 117.9, 117.3, 114.7, 39.7, 35.4. Mp. 125.0–126.0 °C. TLC: R_f 0.24 (hexane/EtOAc = 1:2). IR (KBr): 3105, 2944, 2363, 1619, 1591, 1521, 1448, 1464, 1352, 1286, 1233, 1197, 1076, 886, 754 cm^{-1}. HRMS Calcd for $C_9H_{12}NO_2$: $[M+H]^+$, 166.0863. Found: *m*/*z* 166.0861.

3-Hydroxy-*N*,*N*-diisobutylbenzamide (1c).

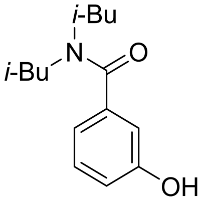

White solid; 66% yield for preparation of **8c**, 58% yield for preparation of **1c**.
^{1}H NMR ($CDCl_3$) δ 8.04 (br s, 1H), 7.14 (dd, *J* = 8.0, 7.5 Hz, 1H), 6.88 (dd, *J* = 2.0, 1.5 Hz, 1H), 6.75 (dd, *J* = 7.5, 1.5 Hz, 1H), 6.74 (dd, *J* = 8.0, 2.0 Hz, 1H), 3.36 (d, *J* = 7.0 Hz, 2H), 3.12 (d, *J* = 7.0 Hz, 2H), 2.12 (m, 1H), 1.84 (m, 1H), 0.98 (d, *J* = 7.0 Hz, 6H), 0.73 (d, *J* = 7.0 Hz, 6H). ^{13}C NMR ($CDCl_3$) δ 173.2, 156.7, 137.3, 129.3, 117.9, 117.0, 115.0, 56.5, 51.2, 26.7, 26.1, 20.1, 19.7. Mp. 108.0–108.5 °C. TLC: R_f 0.32 (hexane/EtOAc = 1:2). IR (KBr): 3150, 2964, 2871, 1595, 1580, 1465, 1449, 1385, 1301, 1261, 1110, 889, 752 cm^{-1}. HRMS Calcd for $C_{15}H_{24}NO_2$: $[M+H]^+$, 250.1802. Found: *m*/*z* 250.1797.

***N*,*N*-Dicyclohexyl-3-hydroxybenzamide (1d)**.

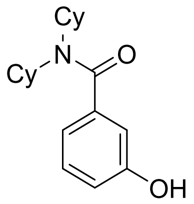

White solid; 75% yield for preparation of **8d**, 85% yield for preparation of **1d**.

^{1}H NMR ($CDCl_3$) δ 8.40 (br s, 1H), 7.08 (dd, *J* = 2.5, 2.5 Hz, 1H), 6.74 (m, 1H), 6.68–6.65 (m, 2H), 3.42 (br s, 1H), 3.03 (br s, 1H), 2.63 (br s, 2H), 1.83 (br s, 2H), 1.69–1.60 (m, 7H), 1.53–1.49 (m, 3H), 1.27 (br s, 3H), 1.02 (br s, 3H). ^{13}C NMR ($CDCl_3$) δ 170.4, 156.9, 138.4, 129.3, 116.7, 116.1, 114.0, 60.0, 56.3, 31.1, 29.9, 26.6, 25.4. Mp. 232.0–232.9 °C. TLC: R_f 0.45 (hexane/EtOAc = 1:1). IR (KBr): 3069, 2938, 2861, 1577, 1469, 1452, 1377, 1321, 1233, 1179, 1125, 999, 781 cm^{-1}. HRMS Calcd for $C_{19}H_{28}NO_2$: $[M+H]^+$, 302.2115. Found: *m*/*z* 302.2107.

(3-Hydroxyphenyl)(piperidin-1-yl)methanone (1e).

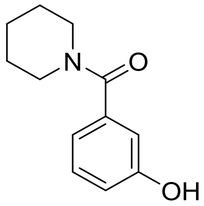

White solid; 99% yield for preparation of **8e**, 97% yield for preparation of **1e**.

^{1}H NMR ($CDCl_3$) δ 7.93 (s, 1H), 7.18 (dd, *J* = 8.0, 7.5 Hz, 1H), 6.98 (dd, *J* = 2.5, 1.5 Hz, 1H), 6.81 (ddd, *J* = 8.0, 2.5, 1.0 Hz, 1H), 6.80 (ddd, *J* = 7.5, 1.5, 1.0 Hz, 1H), 3.71 (br s, 2H), 3.35 (m, 2H), 1.67 (br s, 4H), 1.50 (br s, 2H). ^{13}C NMR ($CDCl_3$) δ 170.0, 157.0, 136.7, 129.4, 117.5, 117.2, 114.6, 48.9, 43.3, 26.5, 25.6, 24.5. Mp. 174.0–175.0 °C. TLC: R_f 0.32 (hexane/EtOAc = 1:2). IR (KBr): 3139, 2964, 2928, 2865, 1603, 1507, 1460, 1348, 1294, 1224, 1156, 1123, 1030, 947, 880, 745, 691 cm^{-1}. HRMS Calcd for $C_{12}H_{16}NO_2$: $[M+H]^+$, 206.1176. Found: *m*/*z* 206.1173.

General procedure for preparation of substrates 1f–1l

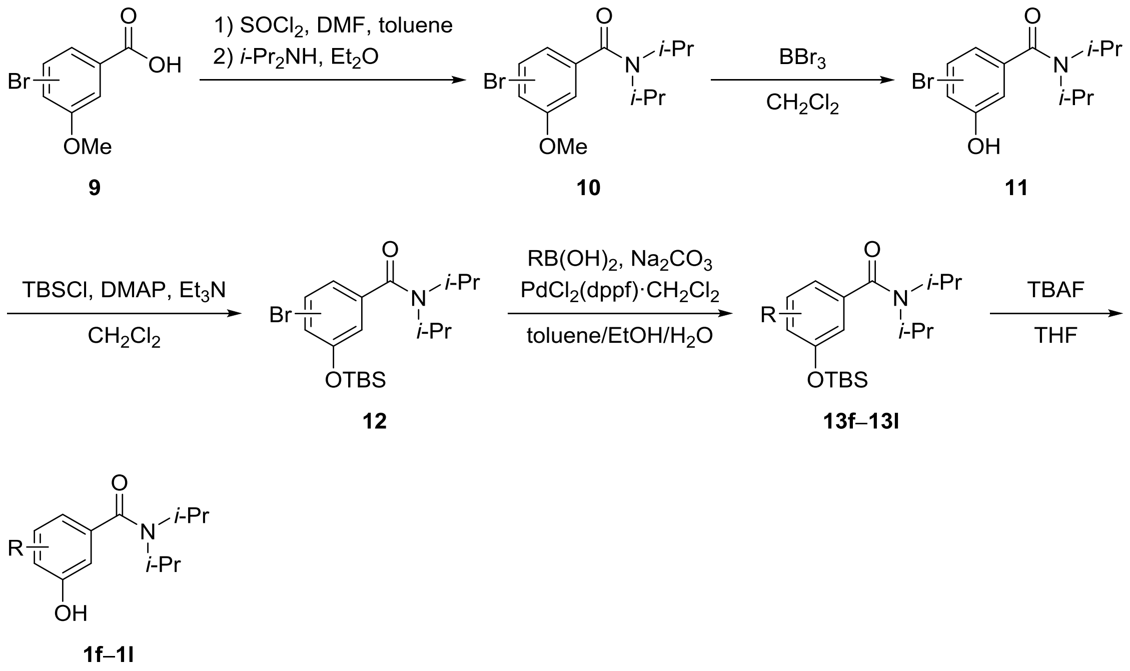

<u>General procedure for preparation of **12**</u>

From 4/5-bromoanisidic acids **9**, 3-hydroxy-4/5-bromobenzamides **11** were prepared by the same procedure as that for the substrates **1a**–**e**. Furthermore, substrates **1f**–**l** were synthesized by the literature procedure.[1] To a 50-mL round-bottom flask, TBSCl (0.45 g, 3.0 mmol) and DMAP (30 mg, 0.25 mmol) were placed, and they were cooled to 0 °C. Subsequently, the solution of the 3-hydroxy-4/5-bromobenzamide (2.5 mmol) in CH_2Cl_2 (20 mL) was added, and the reaction mixture was stirred at ambient temperature. After 20 h, the reaction was quenched with H_2O (20 mL). The aqueous phase was extracted with CH_2Cl_2 (10 mL × 3). Then the combined organic layers were washed with brine and dried over Na_2SO_4. Purification by flush silica gel column chromatography using hexane/EtOAc (v/v = 3/1) as an eluent gave the corresponding products **12** quantitatively.

<u>General procedure for preparation of **13**</u>

In a 30-mL round-bottom flask, $PdCl_2$(dppf)·CH_2Cl_2 (50 mg, 0.061 mmol), Na_2CO_3 (0.75 mg, 7.1 mmol), and H_2O (4.0 mL) were mixed. Then the solution of **12** (1.5 mmol) and a boronic acid (2.0 mmol) in toluene/EtOH (21 mL, v/v = 7/1) was added. The mixture was stirred at 120 °C for 4 h, and then the reaction was quenched with H_2O (10 mL). The aqueous phase was extracted with EtOAc (7.0 mL × 3). The combined organic layers were washed with brine and dried over Na_2SO_4. Purification by flush

silica gel column chromatography using hexane/EtOAc (v/v = 3/1) as an eluent gave the corresponding products **13** in 35–97% yield.

General procedure for preparation of **1f**–**1l**

In a 50-mL round-bottom flask, **13** (1.0 mmol) was placed and dissolved in THF (10 mL). To the solution was added dropwise the solution of TBAF (1.2 mL, c.a. 1.0 M in THF 1.2 mmol), and the mixture was stirred for 1 h. The reaction mixture was quenched with 1.0 M aqueous HCl, and the aqueous phase was extracted with EtOAc (7.0 mL × 3). Then the combined organic layers were washed with brine, and dried over Na_2SO_4. Purification by flush silica gel column chromatography using hexane/EtOAc (v/v = 1/1) as an eluent gave the 4/5-substituted benzamides **1f**–**1l** in 51–99% yield.

General procedure for preparation of **9**

The monobrominated anisidic acids **9** were prepared by the literature procedures.[3,4]

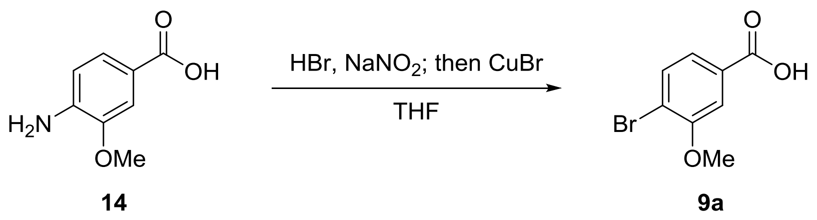

In a 50-mL round-bottom flask, 4-aminoanisidic acid (**14**, 0.84 g, 5.0 mmol) was dissolved in MeCN (10 mL). To the solution was added slowly 47% aqueous HBr (10 mL) at 0 °C, and then $NaNO_2$ (0.38 g, 5.5 mmol) was added to give a brown suspension. After the suspension was stirred for 30 min, CuBr (0.86 g, 6.0 mmol) was added. Subsequently, the reaction mixture was warmed to 50 °C and stirred for 1 h. The reaction mixture was then cooled to 0 °C, and cold H_2O (20 mL) was poured into the flask to form white precipitates. The solid was filtrated, washed with cold H_2O, and dried in vacuo. The reaction proceeded quantitatively to afford **9a**, and the crude product was used without further purification.

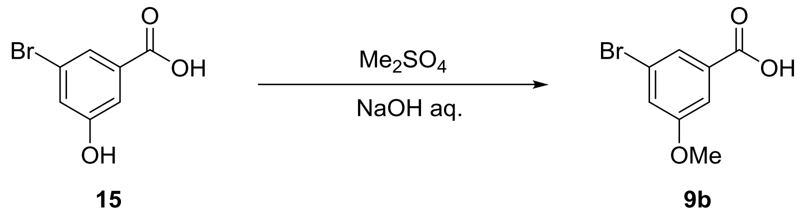

3-Bromo-5-hydroxybenzoic acid (**15**, 2.2 g, 10 mmol) was placed in a 50-mL round-

bottom flask, and dissolved in 10% aqueous NaOH (3.0 mL) to give a yellow solution. To the mixture was added dropwise Me_2SO_4 (1.7 mL, 18 mmol). Then the reaction mixture was warmed to 70 °C. After being stirred for 12 h, the reaction mixture was cooled to ambient temperature and quenched with 20% aqueous H_2SO_4 to give a white solid. The solid was filtrated and dried in vacuo to afford **9b** in 92%, and the crude product was used without further purification.

5-Hydroxy-*N,N*-diisopropyl-[1,1'-biphenyl]-3-carboxamide (1f).

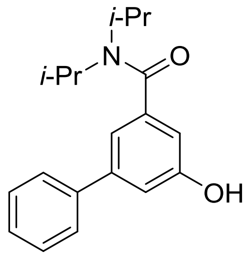

White solid; 88% yield (for the last step).

^{1}H NMR ($CDCl_3$) δ 8.45 (br s, 1H), 7.45 (dd, *J* = 7.0, 1.5 Hz, 2H), 7.36 (m, 2H), 7.32 (m, 1H), 6.94 (dd, *J* = 1.5, 1.0 Hz, 1H), 6.90 (dd, *J* = 2.0, 1.5 Hz, 1H), 6.71 (dd, *J* = 2.0, 1.0 Hz, 1H), 3.80 (br s, 1H), 3.53 (br s, 1H), 1.57 (br s, 6H), 1.14 (br s, 6H). ^{13}C NMR ($CDCl_3$) δ 172.0, 157.2, 142.7, 140.4, 138.7, 128.6, 127.4, 127.1, 115.8, 115.3, 112.8, 51.3, 46.1, 20.6. Mp. 184.5–185.0 °C. TLC: R_f 0.38 (hexane/EtOAc = 1:1). IR (KBr): 3300, 2976, 2936, 1607, 1588, 1471, 1384, 1350, 1304, 1214, 1152, 1041, 865 cm^{-1}. HRMS Calcd for $C_{19}H_{24}NO_2$: $[M+H]^+$, 298.1802. Found: *m/z* 298.1796.

5-Hydroxy-*N,N*-diisopropyl-4'-methoxy-[1,1'-biphenyl]-3-carboxamide (1g).

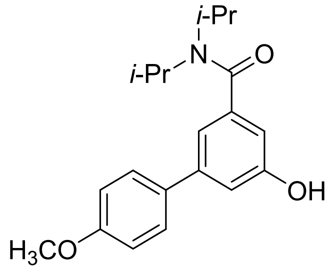

White solid; 91% yield (for the last step).

^{1}H NMR ($CDCl_3$) δ 8.03 (br s, 1H), 7.39 (ddd, *J* = 9.0, 2.5, 2.0 Hz, 2H), 6.91–6.86 (m, 4H), 6.67 (dd, *J* = 2.0, 1.5 Hz, 1H), 3.97 (br s, 1H), 3.82 (s, 3H), 3.53 (br s, 1H), 1.57 (br s, 6H), 1.13 (br s, 6H). ^{13}C NMR ($CDCl_3$) δ 171.9, 159.2, 157.0, 142.3, 138.9, 132.9, 128.1, 115.1, 115.0, 114.0, 112.1, 55.3, 51.2, 46.0, 20.6. Mp. 215.0–216.0 °C. TLC: R_f 0.35 (hexane/EtOAc = 1:1). IR (KBr): 3159, 2971, 1609, 1594, 1520, 1465, 1439,

1363, 1250, 1181, 1041, 829 cm^{-1}. HRMS Calcd for $C_{20}H_{26}NO_3$: $[M+H]^+$, 328.1907. Found: *m*/*z* 328.1899.

5-Hydroxy-*N,N*-diisopropyl-4'-(trifluoromethyl)-[1,1'-biphenyl]-3-carboxamide (1h).

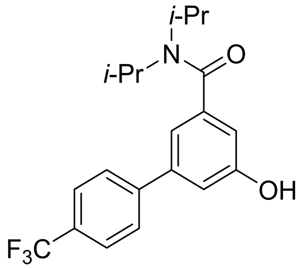

White solid; 96% yield (for the last step).
^{1}H NMR ($CDCl_3$) δ 8.69 (br s, 1H), 7.56 (m, 2H), 7.49 (m, 2H), 6.91 (s, 1H), 6.84 (s, 1H), 6.73 (s, 1H), 3.94 (br s, 1H), 3.55 (br s, 1H), 1.57 (br s, 6H), 1.13 (br s, 6H). ^{13}C NMR ($CDCl_3$) δ 171.9, 143.8, 141.2, 138.9, 129.4 (q, *J* = 33.0 Hz), 127.2, 125.5 (d, *J* = 3.9 Hz), 124.1 (q, *J* = 270.6 Hz), 115.8 (d, *J* = 5.8 Hz), 115.1, 113.4 (d, *J* = 17.6 Hz), 51.4, 46.2, 20.6. ^{19}F NMR ($CDCl_3$) δ 99.2. Mp. 204.0–205.0 °C. TLC: R_f 0.41 (hexane/EtOAc = 1:1). IR (KBr): 3106, 2986, 1610, 1591, 1477, 1438, 1373, 1326, 1221, 1163, 1118, 1064, 836 cm^{-1}. HRMS Calcd for $C_{20}H_{23}F_3NO_2$: $[M+H]^+$, 366.1675. Found: *m*/*z* 366.1668.

3-Hydroxy-*N,N*-diisopropyl-5-(naphthalen-2-yl)benzamide (1i).

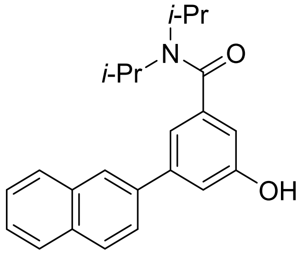

White solid; 84% yield (for the last step).
^{1}H NMR ($CDCl_3$) δ 8.27 (br s, 1H), 7.86 (s, 1H), 7.80 (dd, *J* = 7.0, 1.5 Hz, 1H), 7.79 (d, *J* = 8.5 Hz, 1H), 7.69 (d, *J* = 7.0 Hz, 1H), 7.58 (dd, *J* = 8.5, 2.0 Hz, 1H), 7.45 (ddd, *J* = 7.0, 7.0, 2.0 Hz, 1H), 7.42 (ddd, *J* = 7.0, 7.0, 1.5 Hz, 1H), 7.08 (dd, *J* = 1.5, 1.5 Hz, 1H), 7.01 (dd, *J* = 2.0, 1.5 Hz, 1H), 6.74 (dd, *J* = 2.0, 1.5 Hz, 1H), 4.01 (br s, 1H), 3.56 (br s, 1H), 1.59 (br s, 6H), 1.15 (br s, 6H). ^{13}C NMR ($CDCl_3$) δ 172.0, 157.1, 142.7, 138.9, 137.7, 133.5, 132.6, 128.3, 128.2, 127.5, 126.2, 125.9, 125.8, 125.3, 116.0, 115.7, 112.9, 51.3, 46.1, 20.6. Mp. 232.0–233.0 °C. TLC: R_f 0.41 (hexane/EtOAc = 1:1). IR

(KBr): 3254, 2980, 1616, 1586, 1479, 1445, 1414, 1345, 1215, 1153, 1044, 858, 810 cm^{-1}. HRMS Calcd for $C_{23}H_{26}NO_2$: $[M+H]^+$, 348.1958. Found: *m*/*z* 348.1951.

3-Cyclopropyl-5-hydroxy-*N*,*N*-diisopropylbenzamide (1j).

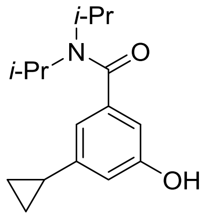

White solid; 61% yield (for the last step).

^{1}H NMR ($CDCl_3$) δ 7.94 (br s, 1H), 6.49 (dd, *J* = 2.0, 1.5 Hz, 1H), 6.44 (dd, *J* = 1.5, 1.0 H, 1H), 6.40 (dd, *J* = 2.0, 1.0 Hz, 1H), 3.89 (br s, 1H), 3.49 (br s, 1H), 1.77 (m, 1H), 1.53 (br s, 6H), 1.11 (br s, 6H), 0.90 (m, 2H), 0.62 (m, 2H). ^{13}C NMR ($CDCl_3$) δ 172.1, 156.8, 146.0, 138.4, 114.1, 113.7, 110.7, 51.1, 45.9, 20.6, 15.3, 9.3. Mp. 101.0–102.0 °C. TLC: R_f 0.33 (hexane/EtOAc = 1:1). IR (KBr): 3242, 3002, 2971, 1610, 1592, 1458, 1372, 1347, 1280, 1208, 1158, 1041, 997, 866, 848, 766 cm^{-1}. HRMS Calcd for $C_{16}H_{24}NO_2$: $[M+H]^+$, 262.1802. Found: *m*/*z* 262.1800.

2-Hydroxy-*N*,*N*-diisopropyl-[1,1'-biphenyl]-4-carboxamide (1k).

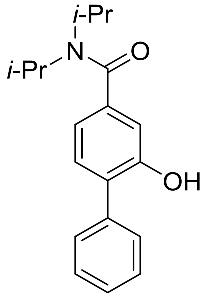

White solid; 57% yield (for the last step).

^{1}H NMR ($CDCl_3$) δ 7.43–7.42 (m, 4H), 7.34 (m, 1H), 7.18 (dd, *J* = 7.5, 1.5 Hz, 1H), 7.15 (br s, 1H), 6.91 (d, *J* = 1.5 Hz, 1H), 6.83 (m, 1H), 4.01 (br s, 1H), 3.53 (br s, 1H), 1.55 (br s, 6H), 1.17 (br s, 6H). ^{13}C NMR ($CDCl_3$) δ 171.3, 153.3, 138.3, 137.3, 130.3, 129.1, 128.6, 127.4, 117.1, 114.1, 109.7, 51.1, 48.0, 20.6. Mp. 92.0–93.0 °C. TLC: R_f 0.42 (hexane/EtOAc = 1:1). IR (KBr): 3179, 2970, 1601, 1457, 1408, 1371, 1346, 1275, 1208, 1158, 1038, 877, 808, 759 cm^{-1}. HRMS Calcd for $C_{19}H_{24}NO_2$: $[M+H]^+$, 298.1802. Found: *m*/*z* 298.1795.

4-Cyclopropyl-3-hydroxy-*N,N*-diisopropylbenzamide (1l).

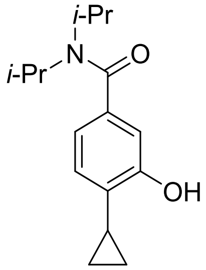

White solid; 78% yield (for the last step).
^{1}H NMR ($CDCl_3$) δ 7.09 (br s, 1H), 6.88 (d, *J* = 7.5 Hz, 1H), 6.76 (d, *J* = 1.5 Hz, 1H), 6.69 (dd, *J* = 7.5, 1.5 Hz, 1H), 3.91 (br s, 1H), 3.50 (br s, 1H), 1.87 (m, 1H), 1.53 (br s, 6H), 1.24 (br s, 6H), 0.91 (m, 2H), 0.59 (m, 2H). ^{13}C NMR ($CDCl_3$) δ 171.5, 155.5, 136.8, 129.5, 127.0, 116.8, 112.8, 51.0, 45.8, 20.6, 9.25, 6.24. Mp. 88.5–89.0 °C. TLC: R_f 0.48 (hexane/EtOAc = 1:1). IR (KBr): 3304, 3083, 2969, 1607, 1523, 1455, 1416, 1373, 1347, 1269, 1230, 1209, 1159, 1109, 1046, 1034, 957, 908, 891, 814 cm^{-1}. HRMS Calcd for $C_{16}H_{24}NO_2$: $[M+H]^+$, 262.1802. Found: *m*/*z* 262.1800.

Preparation of substrates 1m and 1n

Monobromoinated substrates **1m** and **1l** were prepared by the literature procedure.[1]

2-Bromo-3-hydroxy-*N,N*-diisopropylbenzamide (1m).

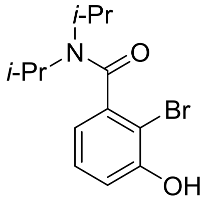

Whtie solid; 56% yield (overall).
^{1}H NMR ($CDCl_3$) δ 8.72 (br s, 1H), 7.21 (d, *J* = 8.5 Hz, 1H), 6.52 (dd, *J* = 8.5, 3.0 Hz, 1H), 6.50 (d, *J* = 3.0 Hz, 1H), 3.69 (qq, *J* = 7.0, 7.0 Hz, 1H), 3.54 (qq, *J* = 7.0, 7.0 Hz, 1H), 1.58 (d, *J* = 7.0 Hz, 3H), 1.56 (d, *J* = 7.0 Hz, 3H), 1.24 (d, *J* = 7.0 Hz, 3H), 1.06 (d, *J* = 7.0 Hz, 3H). ^{13}C NMR ($CDCl_3$) δ 167.6, 152.6, 138.5, 133.4, 118.4, 114.7, 107.4, 51.6, 46.2, 20.6, 20.4, 19.9. Mp. 236.0–237.0 °C. TLC: R_f 0.39 (hexane/EtOAc = 1:1). IR (KBr): 3160, 1616, 1569, 1466, 1444, 1374, 1350, 1289, 1235, 1208, 1164, 1044, 875, 823 cm^{-1}. HRMS Calcd for $C_{13}H_{19}BrNO_2$: $[M+H]^+$, 300.0594. Found: *m*/*z* 300.0583.

2-Bromo-5-hydroxy-*N*,*N*-diisopropylbenzamide (1n).

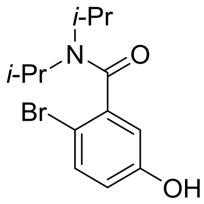

White solid; 48% yield (overall).

^{1}H NMR ($CDCl_3$) δ 8.72 (br s, 1H), 7.23 (dd, *J* = 8.0, 2.5 Hz, 1H), 6.98 (dd, *J* = 8.0, 1.5 Hz, 1H), 6.75 (dd, *J* = 2.5, 1.5 Hz, 1H), 3.63 (qq, *J* = 6.5, 6.5 Hz, 1H), 3.52 (qq, *J* = 6.5, 6.5 Hz, 1H), 1.58 (d, *J* = 6.5 Hz, 3H), 1.56 (d, *J* = 6.5 Hz, 3H), 1.21 (d, *J* = 6.5 Hz, 3H), 1.08 (d, *J* = 6.5 Hz, 3H). ^{13}C NMR ($CDCl_3$) δ 167,6, 152.6, 140.6, 129.3, 118.2, 115.4, 107.2, 51.2, 46.0, 20.74, 20.66, 20.60, 20.1. Mp. 258.0–259.0 °C. TLC: R_f 0.43 (hexane/EtOAc = 1:1). IR (KBr): 3225, 2971, 1608, 1567, 1447, 1372, 1351, 1292, 1199, 1121, 1042, 822, 797 cm^{-1}. HRMS Calcd for $C_{13}H_{19}BrNO_2$: $[M+H]^+$, 300.0594. Found: *m*/*z* 300.0588.

Preparation of substrates 5 (8a)

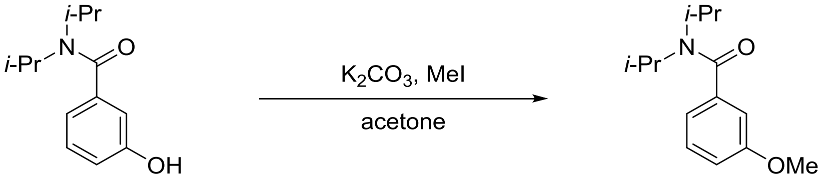

1a **5**

To a 30-mL round-bottom flask were sequentially added **1a** (1.0 mmol), K_2CO_3 (5.0 mmol), acetone (4.2 mL) and CH_3I (10 mmol). The mixture was refluxed at 70 ℃ for 24 h. The reaction mixture was passed through a short silica gel pad to remove K_2CO_3 and concentrated in vacuo. Purification by silica gel column chromatography using hexane/EtOAc (v/v = 3/1) as an eluent gave the corresponding substrate **5** (**8a**). (For another synthetic route to **5** (**8a**), see "***General procedure for preparation of substrates 1a–1e***".)

***N*,*N*-Diisopropyl-3-methoxybenzamide (5 (8a))**: CAS RN [103258-40-8].

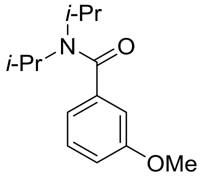

White solid; 69% yield.

^{1}H NMR ($CDCl_3$) δ 7.27 (dd, *J* = 8.0, 7.5 Hz, 1H), 6.90–6.83 (m, 3H), 3.84 (br s, 1H), 3.81 (s, 3H), 3.49 (br s, 1H), 1.53 (br s, 6H), 1.13 (br s, 6H). ^{13}C NMR ($CDCl_3$) δ 170.5, 159.4, 140.0, 129.4, 117.4, 114.3, 110.7, 55.1, 50.7, 45.6, 20.5.

Table S1. Screening of Catalysts[a]

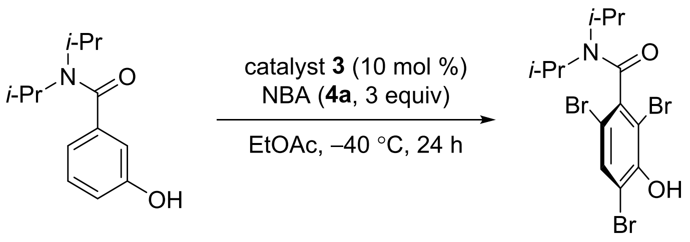

1a **2a**

Entry	catalyst	yield (%)[b]	ee (%)
1	**3a**	84	87
2	**3b**	56	84
3	**3c**	89	–81
4	**3d**	76	–80
5	**3e**	37	41
6	**3f**	51	77
7	**3g**	74	60
8	**3h**	64	83
9	**3i**	86	71
10	**3j**	70	–30
11	**3k**	17	36
12	**3l**	82	16
13	**3m**	97	20
14	**3n**	43	<1

[a] Reactions were run using **1a** (0.1 mmol), the catalyst (0.01 mmol), and the brominating reagent (0.3 mmol) in the solvent (10 mL). [b] Isolated yields. [c] Ar = 3,5-$(CF_3)_2C_6H_3$.

(Table S1)

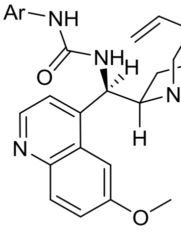

3a

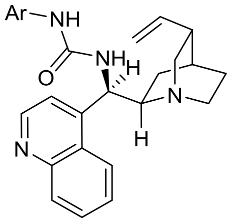

3b

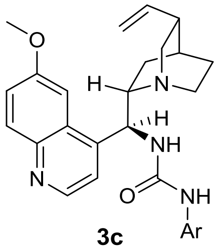

3c

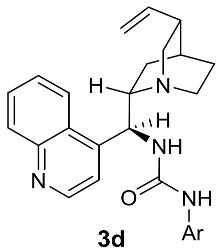

3d

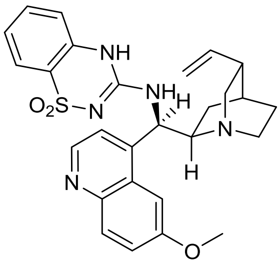

3e

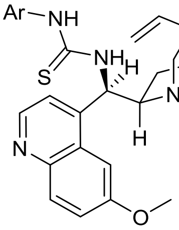

3f

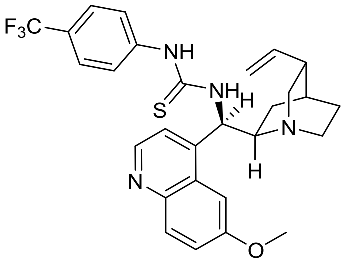

3g

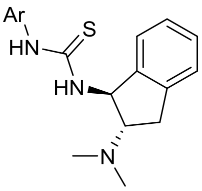

3h

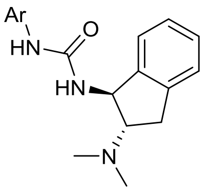

3i

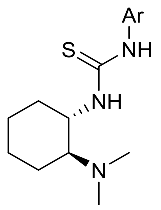

3j

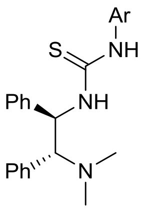

3k

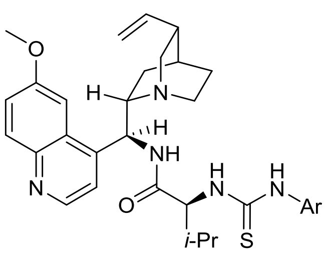

3l

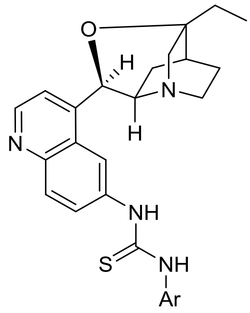

3m

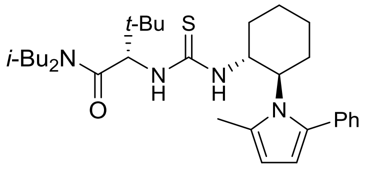

3n

Ar = 3,5-$(CF_3)_2C_6H_3$

Scheme S1. Reaction with 1 Equivalent of **4a**.

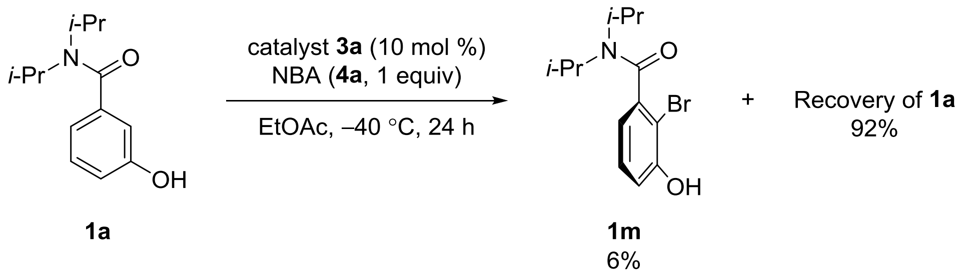

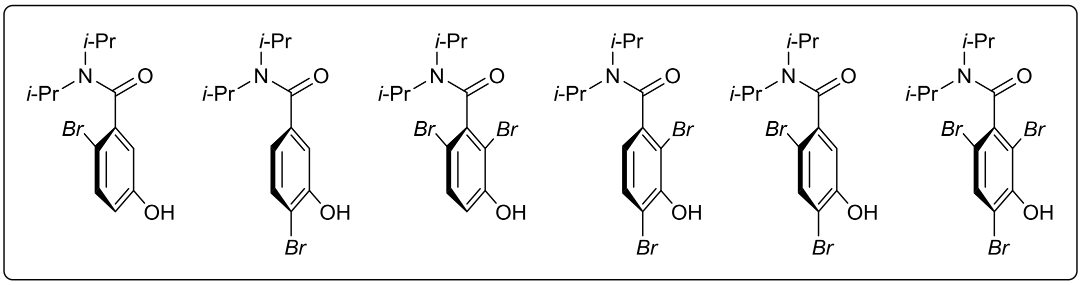

not detected

Characterization Data of Products

2,4,6-Tribromo-3-hydroxy-*N*,*N*-diisopropylbenzamide (2a).

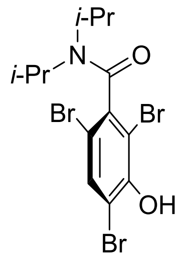

Yield: 84%, 87% *ee*, white solid. $[\alpha]_D^{18}$ –11.4 (*c* 1.05, CH_2Cl_2). ^{1}H NMR ($CDCl_3$) δ 7.67 (s, 1H), 6.28 (s, 1H), 3.53 (m, 2H), 1.59 (d, *J* = 3.0 Hz, 3H), 1.58 (d, *J* = 3.0 Hz, 3H), 1.24 (d, *J* = 7.0 Hz, 3H), 1.22 (d, *J* = 7.0 Hz, 3H). ^{13}C NMR ($CDCl_3$) δ 165.2, 149.6, 139.9, 135.2, 110.3, 109.8, 108.6, 51.9, 46.6, 21.0, 20.9, 19.99, 19.97. Mp. 258.0–258.5 °C. TLC: R_f 0.44 (hexane/EtOAc = 1:1). IR (KBr): 2982, 2598, 1605, 1539, 1474, 1448, 1370, 1329, 1208, 1159, 1132, 1045, 700 cm^{-1}. HRMS Calcd for $C_{13}H_{17}Br_3NO_2$: [M+H]$^+$, 457.8783. Found: *m*/*z* 457.8778. HPLC (Daicel Chiralpak ID-H, hexane/*i*-PrOH = 90/10, flow rate = 2.0 mL/min, λ = 254 nm, 40 °C): t_{minor} = 5.1 min, t_{major} = 9.2 min.

2,4,6-Tribromo-3-hydroxy-*N*,*N*-dimethylbenzamide (2b).

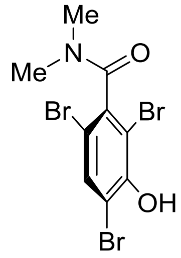

Yield: 83%, 6% *ee*, white solid. $[\alpha]_D^{18}$ –1.7 (*c* 0.98, CH_2Cl_2). ^{1}H NMR ($CDCl_3$) δ 7.65 (s, 1H), 6.65 (br s, 1H), 3.16 (s, 3H), 2.86 (s, 3H). ^{13}C NMR ($CDCl_3$) δ 166.7, 149.8, 139.0, 135.0, 111.3, 109.7, 108.7, 37.4, 34.6. Mp. 145.5–146.0 °C. TLC: R_f 0.29 (hexane/EtOAc = 1:2). IR (KBr): 3031, 2980, 2487, 1605, 1541, 1470, 1434, 1368, 1200, 1158, 1124, 864, 700 cm^{-1}. HRMS Calcd for $C_9H_9Br_3NO_2$: [M+H]$^+$, 401.8157. Found: *m*/*z* 401.8145. HPLC (Daicel Chiralpak ID-H, hexane/*i*-PrOH = 90/10, flow rate = 2.0 mL/min, λ = 254 nm, 40 °C): t_{minor} = 7.3 min, t_{major} = 10.0 min.

2,4,6-Tribromo-3-hydroxy-*N*,*N*-diisobutylbenzamide (2c).

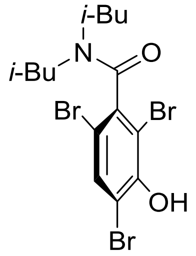

Yield: 80%, 8% *ee*, white solid. $[\alpha]_D^{18}$ –3.3 (*c* 1.01, CH_2Cl_2). ^1H NMR ($CDCl_3$) δ 7.67 (s, 1H), 6.70 (br s, 1H), 3.41 (dd, *J* = 13.5, 7.0 Hz, 1H), 3.34 (dd, *J* = 13.5, 6.5 Hz, 1H), 2.97 (br s, 1H), 2.96 (br s, 1H), 2.19 (m, 1H), 1.87 (m, 1H), 1.03 (br s, 3H), 1.01 (br s, 3H), 0.84 (d, *J* = 6.5 Hz, 3H), 0.83 (d, *J* = 7.0 Hz, 3H). ^{13}C NMR ($CDCl_3$) δ 166.9, 149.6, 139.2, 135.3, 110.7, 110.4, 109.2, 56.6, 53.2, 26.9, 26.7, 20.99, 20.97, 20.60, 20.57. Mp. 114.0–114.5 °C. TLC: R_f 0.40 (hexane/EtOAc = 1:1). IR (KBr): 3362, 2989, 1611, 1466, 1431, 1389, 1362, 1271, 1258, 1205, 1174, 1131, 760 cm^{-1}. HRMS Calcd for $C_{15}H_{21}Br_3NO_2$: $[M+H]^+$, 485.9096. Found: *m*/*z* 485.9085. HPLC (Daicel Chiralpak ID-H, hexane/*i*-PrOH = 90/10, flow rate = 2.0 mL/min, λ = 254 nm, 40 °C): t_{minor} = 3.9 min, t_{major} = 6.3 min.

(*R*)- 2,4,6-Tribromo-*N*,*N*-dicyclohexyl-3-hydroxybenzamide (2d).

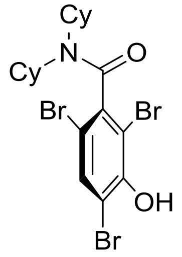

Yield: 99%, 79% *ee*, white solid. $[\alpha]_D^{18}$ –19.1 (*c* 2.99, CH_2Cl_2). ^1H NMR ($CDCl_3$) δ 7.93 (br s, 1H), 7.58 (s, 1H), 3.10 (m, 1H), 3.07 (m, 1H), 2.72–2.67 (m, 2H), 2.02 (d, *J* = 12.0 Hz, 1H), 1.97 (d, *J* = 12.5 Hz, 1H), 1.85–1.83 (m, 2H), 1.73–1.67 (m, 4H), 1.62 (m, 1H), 1.56 (d, *J* = 9.0 Hz, 1H), 1.48–1.43 (m, 2H), 1.29–1.20 (m, 3H), 1.10–1.04 (m, 3H). ^{13}C NMR ($CDCl_3$) δ 166.0, 150.0, 139.5, 135.3, 111.9, 109.9, 109.7, 61.1, 59.8, 31.11, 31.08, 29.2, 29.1, 26.4, 25.54, 25.46, 25.2, 25.1. Mp. 221.5–222.0 °C. TLC: R_f 0.52 (hexane/EtOAc = 2:1). IR (KBr): 3094, 2935, 2855, 1719, 1616, 1534, 1441, 1363, 1314, 1280, 1180, 999, 896, 759 cm^{-1}. HRMS Calcd for $C_{19}H_{25}Br_3NO_2$: $[M+H]^+$, 537.9409. Found: *m*/*z* 537.9400. HPLC (Daicel Chiralpak ID-H, hexane/*i*-PrOH = 90/10, flow rate = 2.0 mL/min, λ = 254 nm, 40 °C): t_{minor} = 4.9 min, t_{major} = 8.4 min.

Piperidin-1-yl(2,4,6-tribromo-3-hydroxyphenyl)methanone (2e).

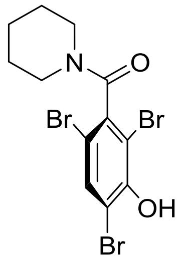

Yield: 93%, 57% *ee*, white solid. $[\alpha]_D^{18}$ –9.8 (*c* 1.42, CH_2Cl_2). ^1H NMR ($CDCl_3$) δ 7.62 (s, 1H), 7.14 (br s, 1H), 3.75 (d, J = 4.5 Hz, 2H), 3.17 (dd, J = 5.0, 4.5 Hz, 2H), 1.68 (br s, 4H), 1.60 (m, 2H). ^{13}C NMR ($CDCl_3$) δ 165.0, 149.9, 138.7, 135.0, 111.7, 109.8, 109.2, 47.4, 42.5, 26.1, 25.2, 24.3. Mp. 169.5–170.0 °C. TLC: R_f 0.25 (hexane/EtOAc = 1:1). IR (KBr): 3078, 2945, 1615, 1533, 1477, 1451, 1402, 1296, 1221, 1134, 1022, 910, 861, 734 cm^{-1}. HRMS Calcd for $C_{12}H_{13}Br_3NO_2$: $[M+H]^+$, 439.8470. Found: *m*/*z* 439.8458. HPLC (Daicel Chiralpak ID-H, hexane/*i*-PrOH = 90/10, flow rate = 2.0 mL/min, λ = 254 nm, 40 °C): t_{minor} = 8.2 min, t_{major} = 10.6 min.

2,4,6-Tribromo-5-hydroxy-*N*,*N*-diisopropyl-[1,1'-biphenyl]-3-carboxamide (2f).

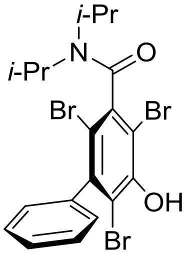

Yield: 86%, 90% *ee*, white solid. $[\alpha]_D^{18}$ –25.6 (*c* 1.53, CH_2Cl_2). ^1H NMR ($CDCl_3$) δ 7.47–7.42 (m, 3H), 7.18–7.17 (m, 2H), 6.37 (br s, 1H), 3.69 (qq, J = 6.5, 6.5 Hz, 1H), 3.55 (qq, J = 7.0, 7.0 Hz, 1H), 1.61 (d, J = 6.5 Hz, 3H), 1.57 (d, J = 7.0 Hz, 3H), 1.27 (d, J = 6.5 Hz, 3H), 1.25 (d, J = 7.0 Hz, 3H). ^{13}C NMR ($CDCl_3$) δ 165.3, 149.5, 143.8, 140.6, 140.4, 129.1, 128.7, 128.5, 128.4, 128.3, 111.8, 111.4, 106.6, 51.8, 46.5, 21.1, 20.8, 20.1, 19.9. Mp. 224.5–225.0 °C. TLC: R_f 0.45 (hexane/EtOAc = 1:1). IR (KBr): 3482, 3070, 2973, 1616, 1544, 1476, 1444, 1377, 1305, 1209, 1160, 1048, 789, 755, 701 cm^{-1}. HRMS Calcd for $C_{19}H_{21}Br_3NO_2$: $[M+H]^+$, 533.9096. Found: *m*/*z* 533.9080. HPLC (Daicel Chiralpak ID-H, hexane/*i*-PrOH = 90/10, flow rate = 2.0 mL/min, λ = 254 nm, 40 °C): t_{minor} = 5.7 min, t_{major} = 9.0 min.

2,4,6-Tribromo-5-hydroxy-*N*,*N*-diisopropyl-4'-methoxy-[1,1'-biphenyl]-3-carboxamide (2g).

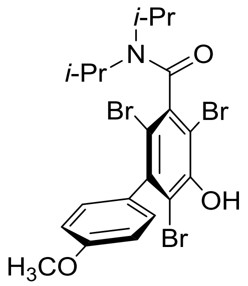

Yield: 88%, 46% *ee*, white solid. $[\alpha]_D^{18}$ –10.1 (*c* 1.20, CH_2Cl_2). ^{1}H NMR ($CDCl_3$) δ 7.12–7.10 (m, 2H), 7.00–6.98 (m, 2H), 6.27 (br s, 1H), 3.86 (s, 3H), 3.68 (qq, *J* = 7.0, 6.5 Hz, 1H), 3.55 (qq, *J* = 7.0, 7.0 Hz, 1H), 1.61 (d, *J* = 7.0 Hz, 3H), 1.57 (d, *J* = 7.0 Hz, 3H), 1.27 (d, *J* = 7.0 Hz, 3H), 1.25 (d, *J* = 6.5 Hz, 3H). ^{13}C NMR ($CDCl_3$) δ 165.9, 159.4, 149.4, 143.6, 140.3, 133.4, 133.0, 130.0, 113.8, 113.6, 112.4, 112.0, 106.4, 55.2, 51.8, 46.5, 21.1, 20.8, 20.1, 19.9. Mp. 263.0–264.0 °C. TLC: R_f 0.43 (hexane/EtOAc = 1:1). IR (KBr): 3072, 2972, 1623, 1609, 1511, 1474, 1370, 1249, 1180, 1046, 832 cm^{-1}. HRMS Calcd for $C_{20}H_{23}Br_3NO_3$: $[M+H]^+$, 563.9202. Found: *m*/*z* 563.9186. HPLC (Daicel Chiralpak ID-H, hexane/*i*-PrOH = 90/10, flow rate = 2.0 mL/min, λ = 254 nm, 40 °C): t_{minor} = 8.3 min, t_{major} = 12.3 min.

2,4,6-Tribromo-5-hydroxy-*N*,*N*-diisopropyl-4'-(trifluoromethyl)-[1,1'-biphenyl]-3-carboxamide (2h).

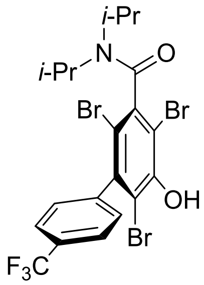

Yield: 99%, 24% *ee*, white solid. $[\alpha]_D^{18}$ –8.7 (*c* 0.89, CH_2Cl_2). ^{1}H NMR ($CDCl_3$) δ 7.34 (d, *J* = 9.0 Hz, 2H), 7.31 (m, 2H), 6.62 (br s, 1H), 3.67 (qq, *J* = 7.0, 7.0 Hz, 1H), 3.56 (qq, *J* = 6.5, 6.5 Hz, 1H), 1.61 (d, *J* = 7.0 Hz, 3H), 1.56 (d, *J* = 7.0 Hz, 3H), 1.27 (d, *J* = 6.5 Hz, 3H), 1.25 (d, *J* = 6.5 Hz, 3H). ^{13}C NMR ($CDCl_3$) δ 165.2, 149.9, 143.9, 142.4, 140.5, 136.6 (q, *J* = 32.5 Hz), 129.7, 129.4, 125.7 (d, *J* = 3.8 Hz), 125.4 (d, *J* = 3.9 Hz), 123.9 (q, *J* = 271.0 Hz), 111.7, 110.9, 107.8, 51.9, 46.6, 21.0, 20.8, 20.1, 19.9. ^{19}F NMR ($CDCl_3$) δ 99.1. Mp. 240.0–241.0 °C. TLC: R_f 0.45 (hexane/EtOAc = 1:1). IR

(KBr): 3300, 2975, 1619, 1539, 1476, 1371, 1322, 1207, 1167, 1126, 1067, 1045, 1022, 953, 850, 689 cm^{-1}. HRMS Calcd for $C_{20}H_{20}Br_3F_3NO_2$: $[M+H]^+$, 601.8970. Found: *m*/*z* 601.8952. HPLC (Daicel Chiralpak ID-H, hexane/*i*-PrOH = 90/10, flow rate = 2.0 mL/min, λ = 254 nm, 40 °C): t_{minor} = 3.7 min, t_{major} = 5.3 min.

2,4,6-Tribromo-3-hydroxy-*N*,*N*-diisopropyl-5-(naphthalen-2-yl)benzamide (2i).

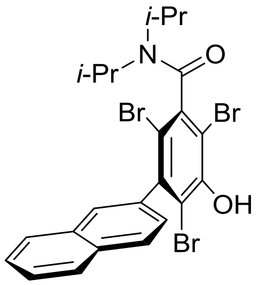

Yield: 86%, 68% *ee*, white solid. $[\alpha]_D^{18}$ –20.1 (*c* 1.00, CH_2Cl_2). ^{1}H NMR ($CDCl_3$) δ 7.98 (br s, 1H), 7.93 (d, *J* = 8.5 Hz, 1H), 7.89 (d, *J* = 3.5 Hz, 1H), 7.88 (d, *J* = 3.0 Hz, 1H), 7.63 (dd, *J* = 8.5, 1.5 Hz, 1H), 7.56 (d, *J* = 1.5 Hz, 1H), 7.54 (d, *J* = 3.5 Hz, 1H), 7.53 (d, *J* = 3.0 Hz, 1H), 3.72 (qq, *J* = 6.5, 2.0 Hz, 1H), 3.58 (qq, *J* = 7.0, 2.5 Hz, 1H), 1.63 (d, *J* = 2.0 Hz, 3H), 1.62 (d, *J* = 2.5 Hz, 3H), 1.30 (d, *J* = 6.5 Hz, 3H), 1.27 (d, *J* = 7.0 Hz, 3H). ^{13}C NMR ($CDCl_3$) δ 165.5, 149.3, 139.4, 133.8, 133.2, 133.0, 132.9, 130.1, 128.3, 128.2, 127.7, 126.7, 126.6, 126.5, 109.8, 109.1, 52.8, 46.5, 21.1, 20.9, 20.12, 20.07. Mp. 248.0–248.5 °C. TLC: R_f 0.46 (hexane/EtOAc = 1:1). IR (KBr): 3508, 3060, 2976, 1642, 1609, 1472, 1443, 1370, 1332, 1281, 1164, 1128, 1053, 858, 820, 750, 689 cm^{-1}. HRMS Calcd for $C_{23}H_{23}Br_3NO_2$: $[M+H]^+$, 583.9252. Found: *m*/*z* 583.9260. HPLC (Daicel Chiralpak ID-H, hexane/*i*-PrOH = 80/20, flow rate = 2.0 mL/min, λ = 254 nm, 40 °C): t_{minor} = 5.0 min, t_{major} = 11.7 min.

2,4,6-Tribromo-3-cyclopropyl-5-hydroxy-*N*,*N*-diisopropylbenzamide (2j).

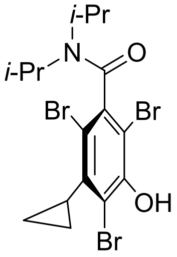

Yield: 69%, 76% *ee*, white solid. $[\alpha]_D^{18}$ –23.1 (*c* 1.54, CH_2Cl_2). ^{1}H NMR ($CDCl_3$) δ 6.38 (br s, 1H), 3.55 (qq, *J* = 6.5, 1.0 Hz, 1H), 3.54 (qq, *J* = 6.5, 1.0 Hz, 1H), 1.74 (m, 1H), 1.60 (d, *J* = 1.0 Hz, 3H), 1.58 (d, *J* = 1.0 Hz, 3H), 1.29–1.26 (m, 2H), 1.24 (d, *J* = 6.5 Hz, 3H), 1.21 (d, *J* = 6.5 Hz, 3H), 0.80 (m, 1H), 0.70 (m, 1H). ^{13}C NMR ($CDCl_3$) δ

165.6, 149.3, 141.4, 140.3, 115.4, 115.0, 105.5, 51.7, 56.5, 21.0, 20.8, 20.1, 19.9, 19.1, 11.8, 11.6. Mp. 138.0–139.0 °C. TLC: R_f 0.37 (hexane/EtOAc = 2:1). IR (KBr): 2978, 2936, 1609, 1534, 1475, 1446, 1381, 1370, 1338, 1208, 1156, 1134, 1045, 853, 821 cm^{-1}. HRMS Calcd for $C_{16}H_{21}Br_3NO_2$: $[M+H]^+$, 497.9096. Found: *m*/*z* 497.9090. HPLC (Daicel Chiralpak ID-H, hexane/*i*-PrOH = 80/20, flow rate = 2.0 mL/min, λ = 254 nm, 40 °C): t_{minor} = 4.8 min, t_{major} = 8.4 min.

3,5-Dibromo-2-hydroxy-*N,N*-diisopropyl-[1,1'-biphenyl]-4-carboxamide (2k).

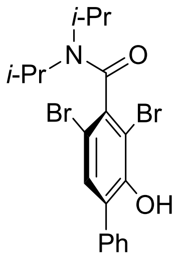

Yield: 73%, 69% *ee*, white solid. $[\alpha]_D^{18}$ –18.5 (*c* 1.32, CH_2Cl_2). ^1H NMR ($CDCl_3$) δ 7.52–7.50 (m, 2H), 7.48–7.45 (m, 3H), 7.40 (m, 1H), 5.89 (br s, 1H), 3.70 (qq, *J* = 6.5, 2.5 Hz, 1H), 3.57 (qq, *J* = 6.5, 2.5 Hz, 1H), 1.62 (d, *J* = 2.5 Hz, 3H), 1.61 (d, *J* = 2.5 Hz, 3H), 1.29 (d, *J* = 6.5 Hz, 3H), 1.26 (d, *J* = 6.5 H, 3H). ^{13}C NMR ($CDCl_3$) δ 165.5, 149.1, 139.4, 135.6, 133.7, 130.1, 129.0, 128.7, 128.4, 109.7, 109.0, 51.8, 46.4, 21.1, 20.9, 20.13, 20.07. Mp. 176.0–177.0 °C. TLC: R_f 0.43 (hexane/EtOAc = 1:1). IR (KBr): 3488, 3061, 2972, 1607, 1479, 1447, 1382, 1370, 1341, 1157, 1056, 1042, 882, 824, 766, 704 cm^{-1}. HRMS Calcd for $C_{19}H_{22}Br_2NO_2$: $[M+H]^+$, 455.9991. Found: *m*/*z* 455.9975. HPLC (Daicel Chiralpak ID-H, hexane/*i*-PrOH = 90/10, flow rate = 2.0 mL/min, λ = 254 nm, 40 °C): t_{minor} = 6.8 min, t_{major} = 11.7 min.

2,6-Dibromo-4-cyclopropyl-3-hydroxy-*N,N*-diisopropylbenzamide (2l).

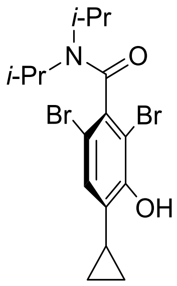

Yield: 99%, 63% *ee,* white solid. $[\alpha]_D^{18}$ –16.6 (*c* 1.04, CH_2Cl_2). ^1H NMR ($CDCl_3$) δ 6.97 (s, 1H), 6.11 (br s, 1H), 3.59 (qq, *J* = 7.0, 3.5 Hz, 1H), 3.52 (qq, *J* = 6.5, 3.0 Hz, 1H), 2.08 (m, 1H), 1.59 (d, *J* = 3.5 Hz, 3H), 1.57 (d, *J* = 3.0 Hz, 3H), 1.23 (d, *J* = 7.0 Hz, 3H),

1.20 (d, J = 6.5 Hz, 3H), 1.02–0.98 (m, 2H), 0.69–0.66 (m, 2H). ^{13}C NMR ($CDCl_3$) δ 165.8, 151.0, 137.3, 132.2, 129.3, 109.4, 108.2, 51.7, 46.4, 21.0, 20.9, 20.1, 20.0, 10.2, 7.7, 7.6. Mp. 134.5–135.5 °C. TLC: R_f 0.60 (hexane/EtOAc = 1:1). IR (KBr): 3000, 2972, 1603, 1483, 1446, 1370, 1341, 1276, 1204, 1164, 1137, 1045, 919, 821, 704 cm^{-1}. HRMS Calcd for $C_{16}H_{22}Br_2NO_2$: $[M+H]^+$, 419.9991. Found: *m*/*z* 419.9985. HPLC (Daicel Chiralpak ID-H, hexane/*i*-PrOH = 80/20, flow rate = 2.0 mL/min, λ = 254 nm, 40 °C): t_{minor} = 4.8 min, t_{major} = 8.3 min.

References

1. Vakulya, B.; Varga, S.; Csámpai, A.; Soós, T. *Org. Lett.* **2005**, *7*, 1967.
2. Barrett, K. T.; Miller, S. J. *J. Am. Chem. Soc.* **2013**, *135*, 2963.
3. Tachdjian, C.; Karanewsky, D. S.; Tang, X.-Q.; Li, X.; Zhang, F.; Servant, G.; Chen, Q.; Darmohusodo, V.; Fine, R.; Fotsing, J. R.; Hammaker, J. R.; Kang, X.; Kimmich, R. D. A.; Klebansky, B.; Liu, H.; Petrovic, G.; Rinnova, M.; Adamski-Werner, S.; Yamamoto, J.; Zhang, H.; Zlotonik, A.; Zoller, M.;Zoller, K. MODULATION OF CHEMOSENSORY RECEPTORS AND LIFANDS ASSOCIATED THEREWITH, U.S. Patent. 2011/0224155 A1, September, 15, 2011.
4. Tamura, Y.; Fukata, F.; Sasho, M.; Tsugoshi, T.; Kita, Y. *J. Org. Chem.* **1985**, *50*, 2273.

2,4,6-Tribromo-3-hydroxy-*N*,*N*-diisopropylbenzamide (2a)

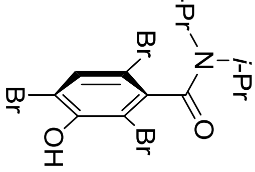

^{1}H NMR

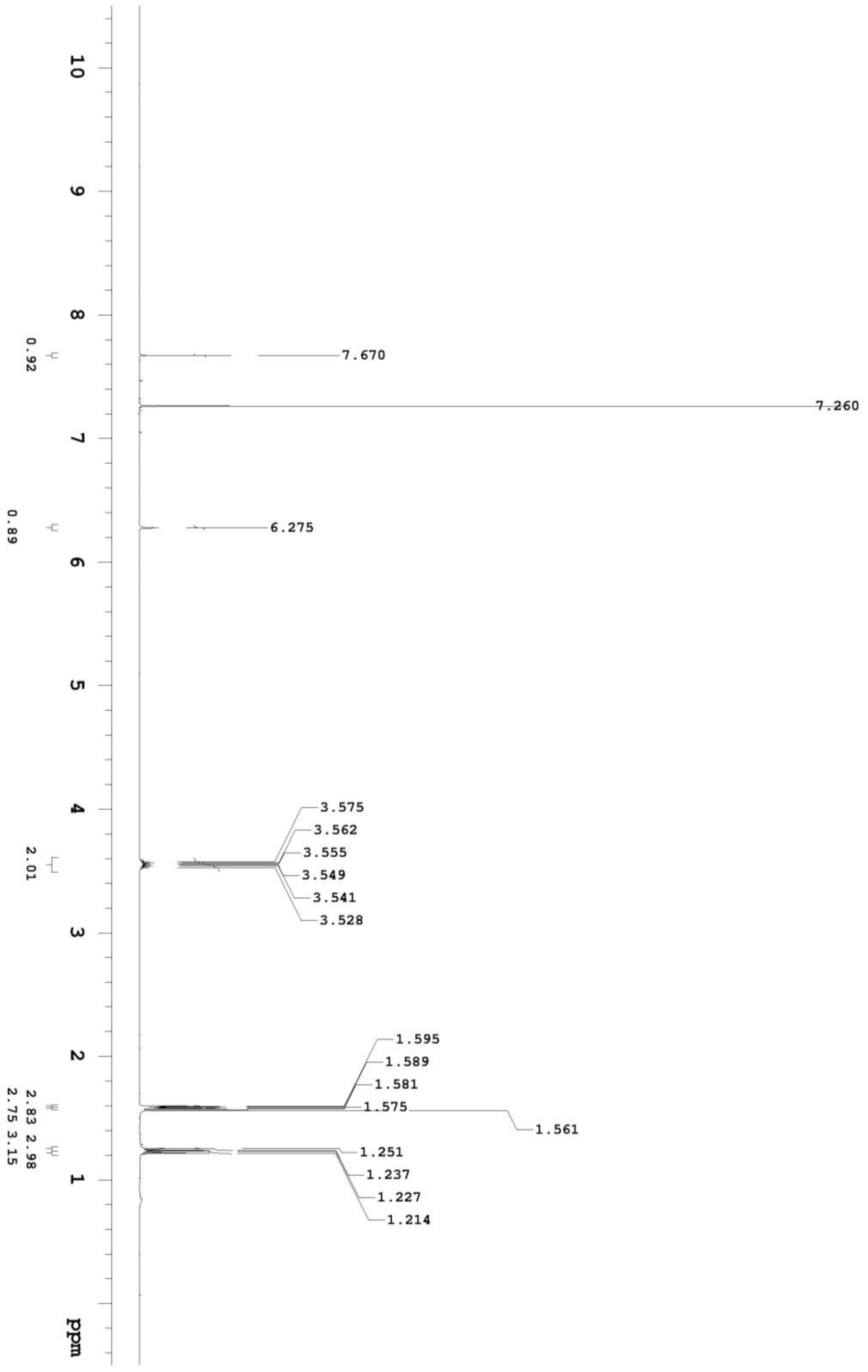

2,4,6-Tribromo-3-hydroxy-*N*,*N*-diisopropylbenzamide (2a)

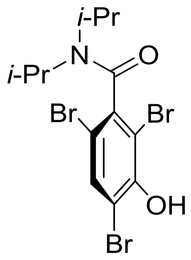

^{13}C NMR

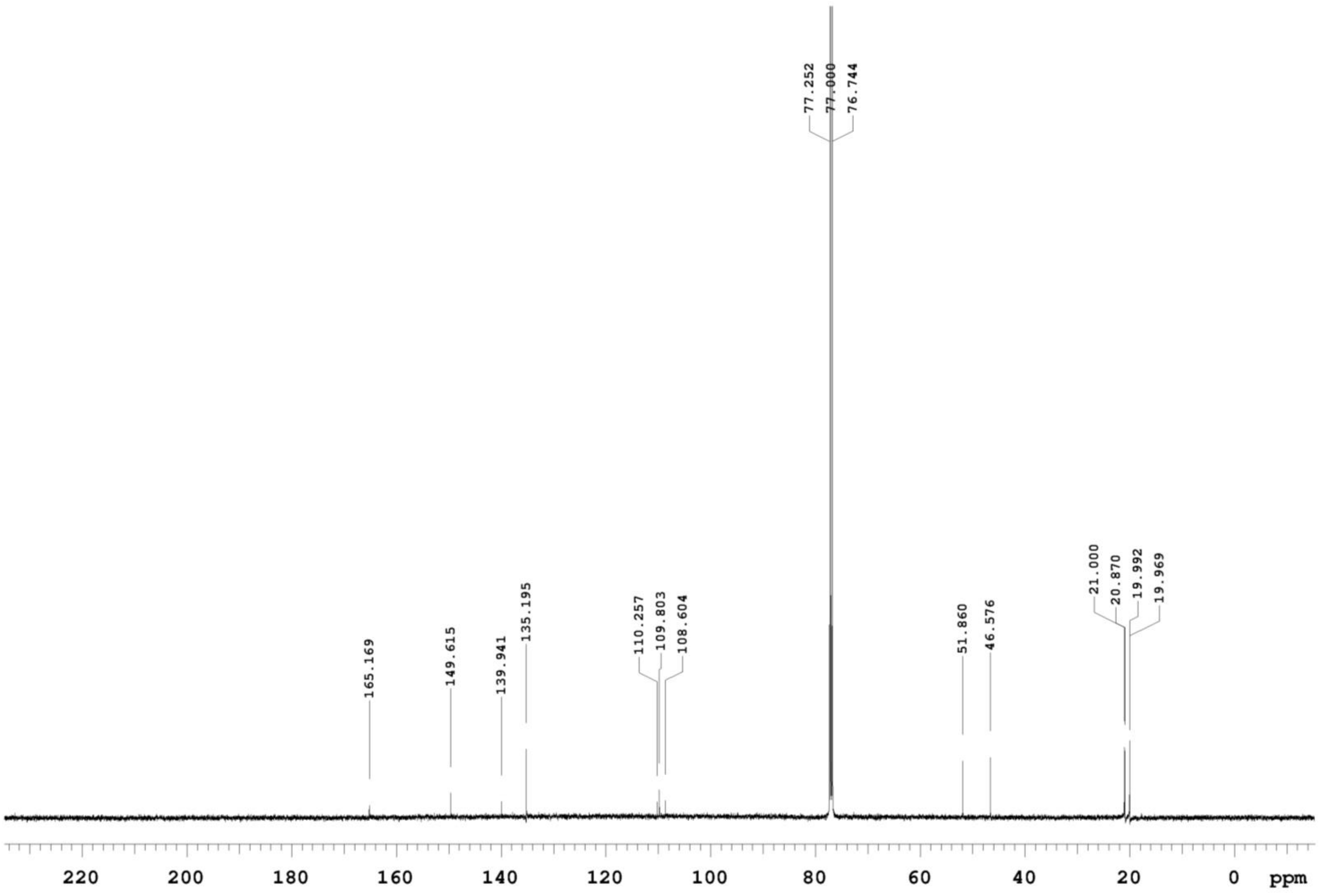

2,4,6-Tribromo-3-hydroxy-*N*,*N*-dimethylbenzamide (2b)

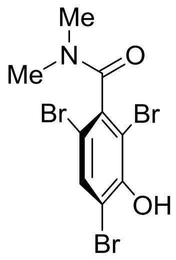

^{1}H NMR

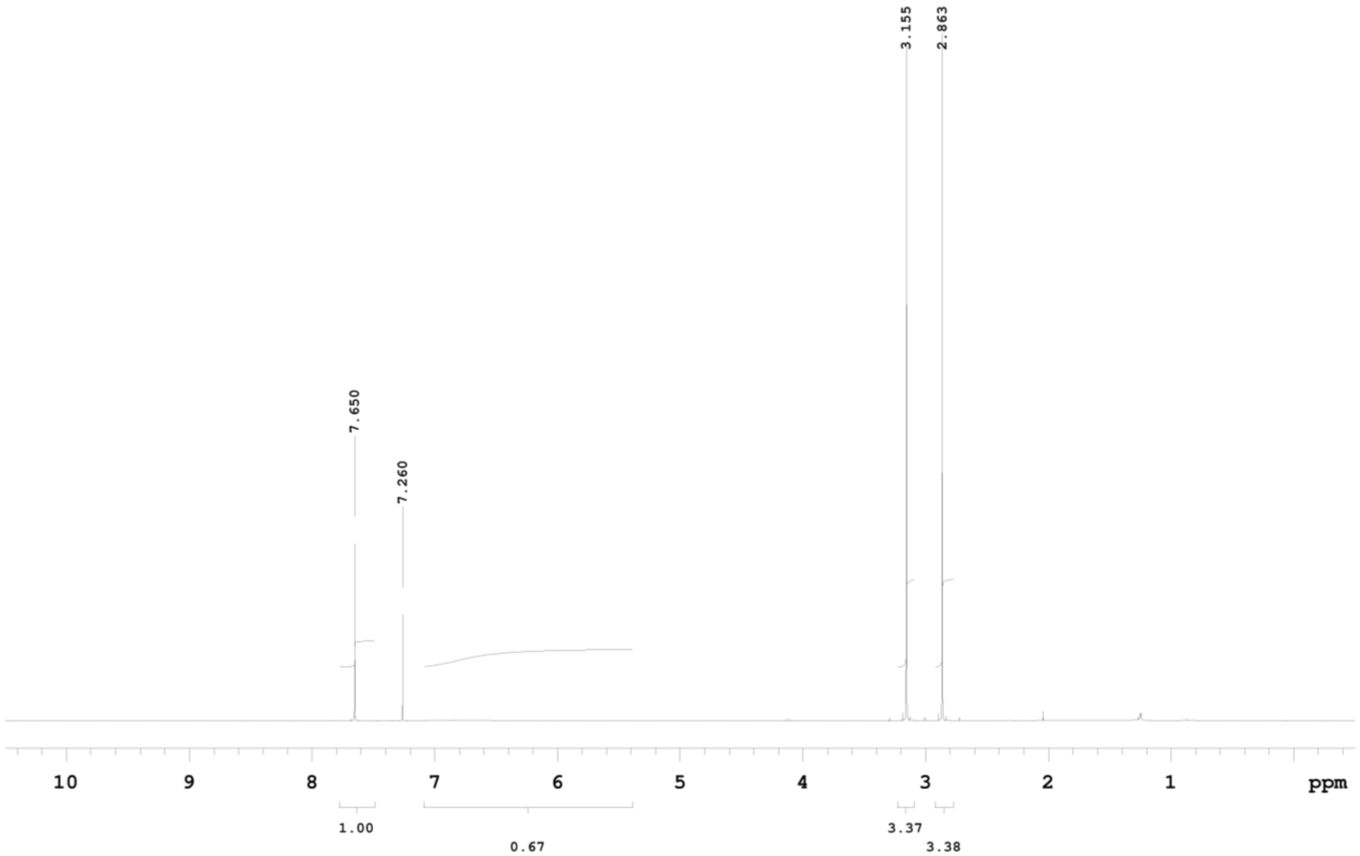

2,4,6-Tribromo-3-hydroxy-*N*,*N*-dimethylbenzamide (2b)

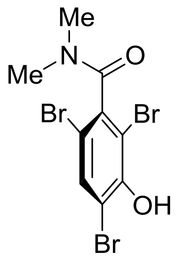

^{13}C NMR

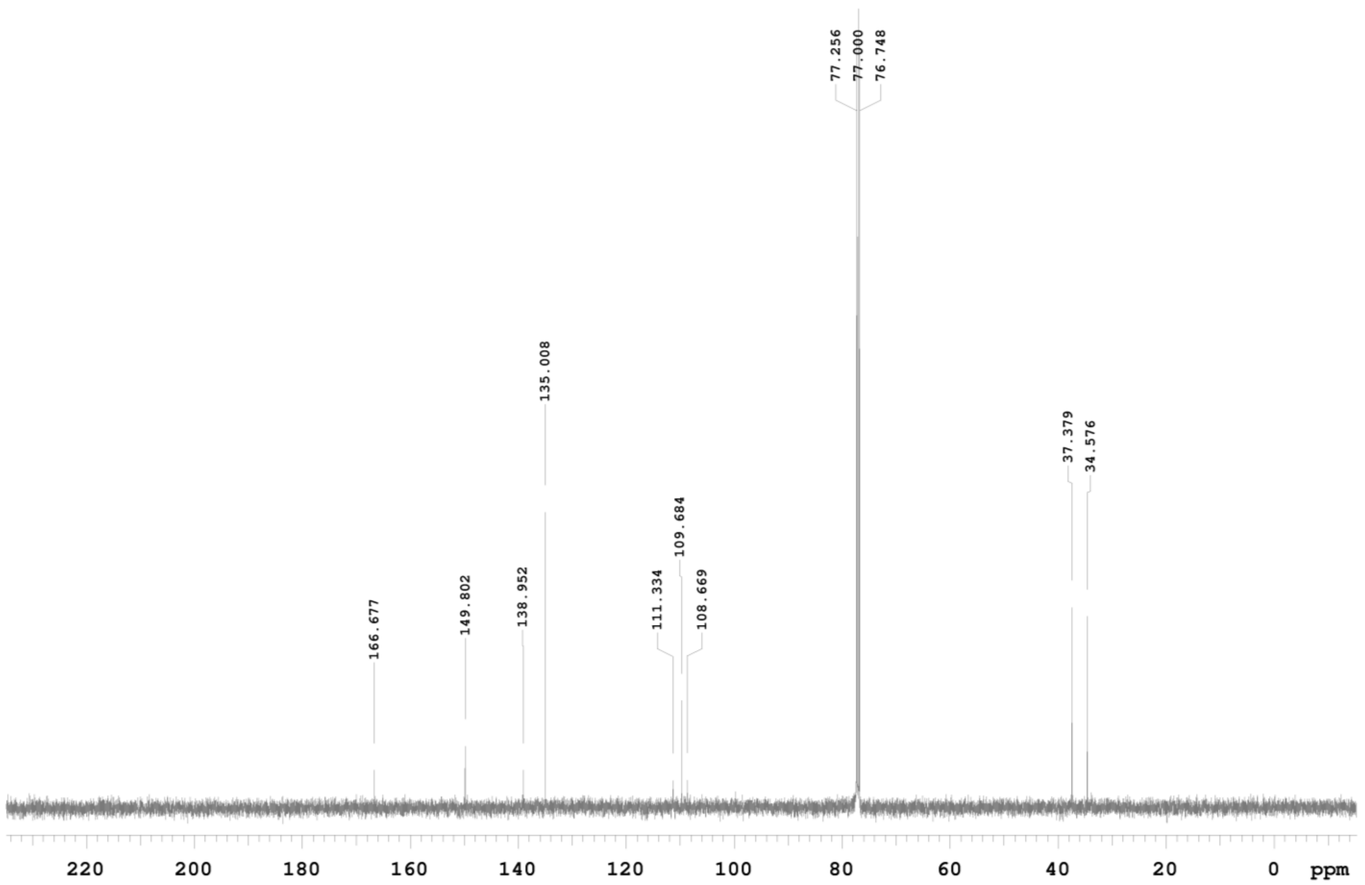

2,4,6-Tribromo-3-hydroxy-*N*,*N*-diisobutylbenzamide (2c)

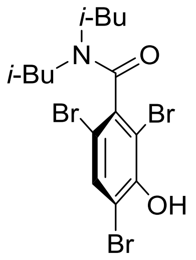

^{1}H NMR

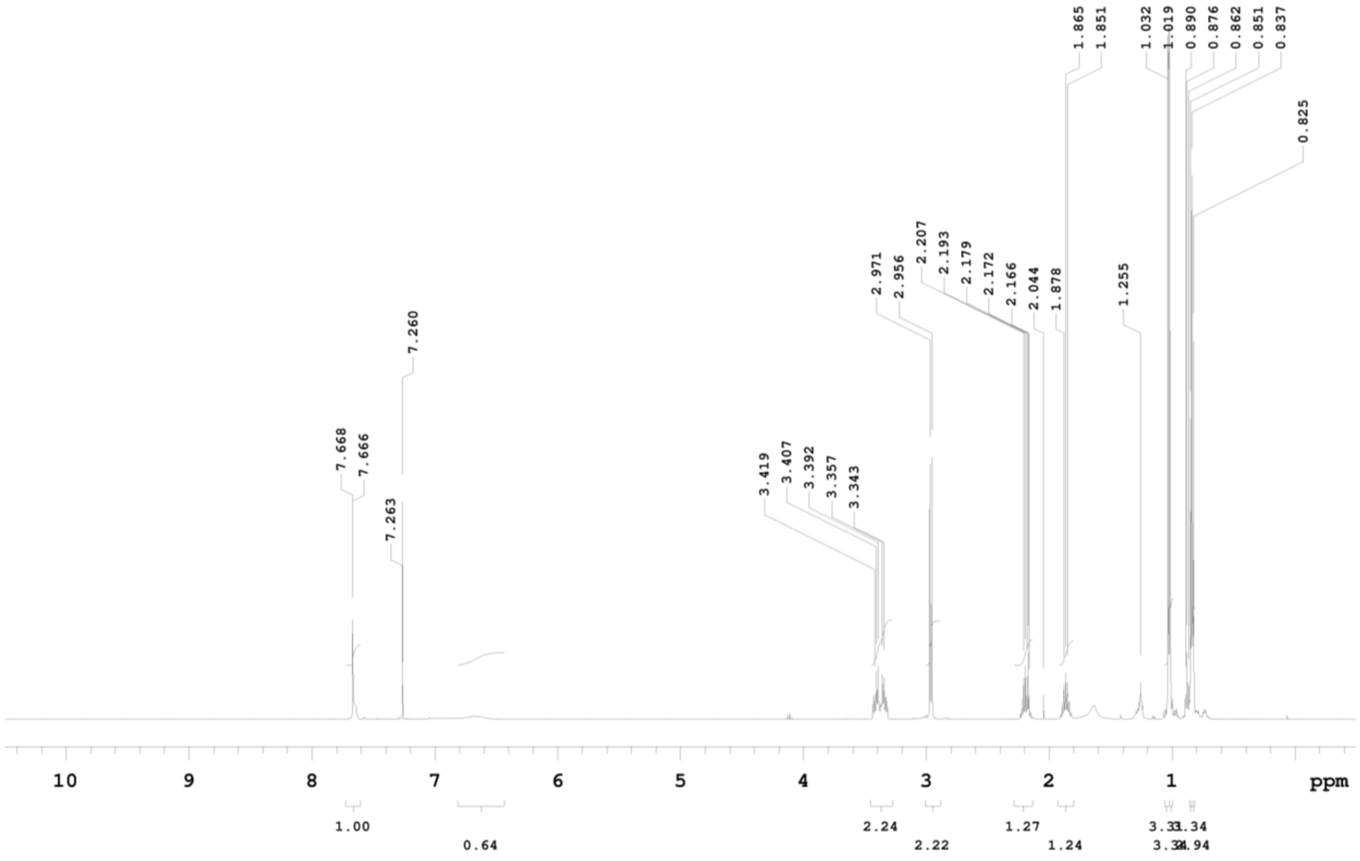

2,4,6-Tribromo-3-hydroxy-*N*,*N*-diisobutylbenzamide (2c)

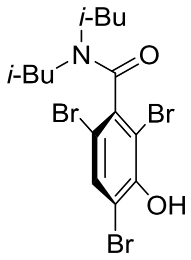

^{13}C NMR

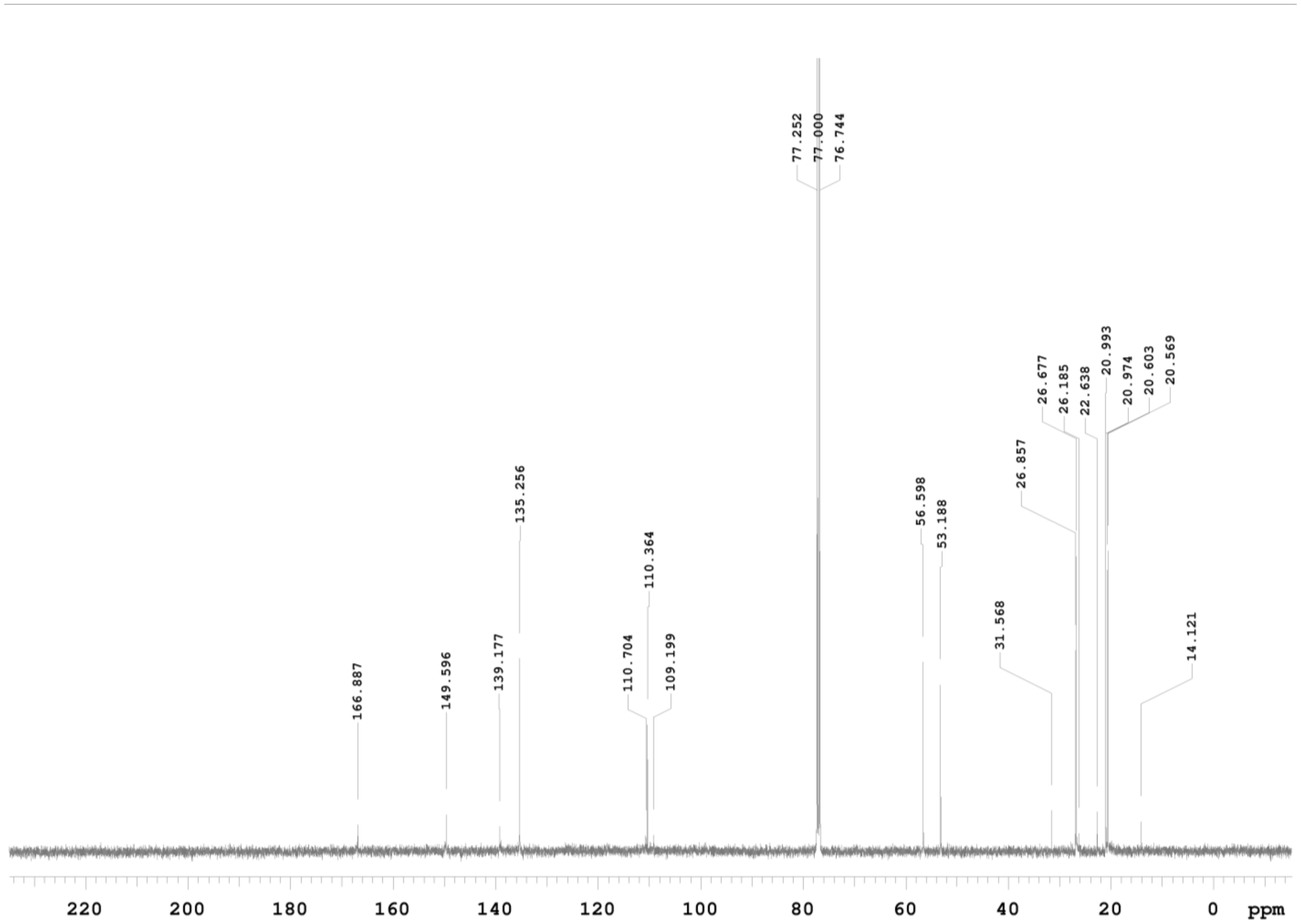

(*R*)-2,4,6-Tribromo-*N*,*N*-dicyclohexyl-3-hydroxybenzamide (2d)

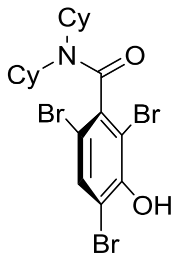

^{1}H NMR

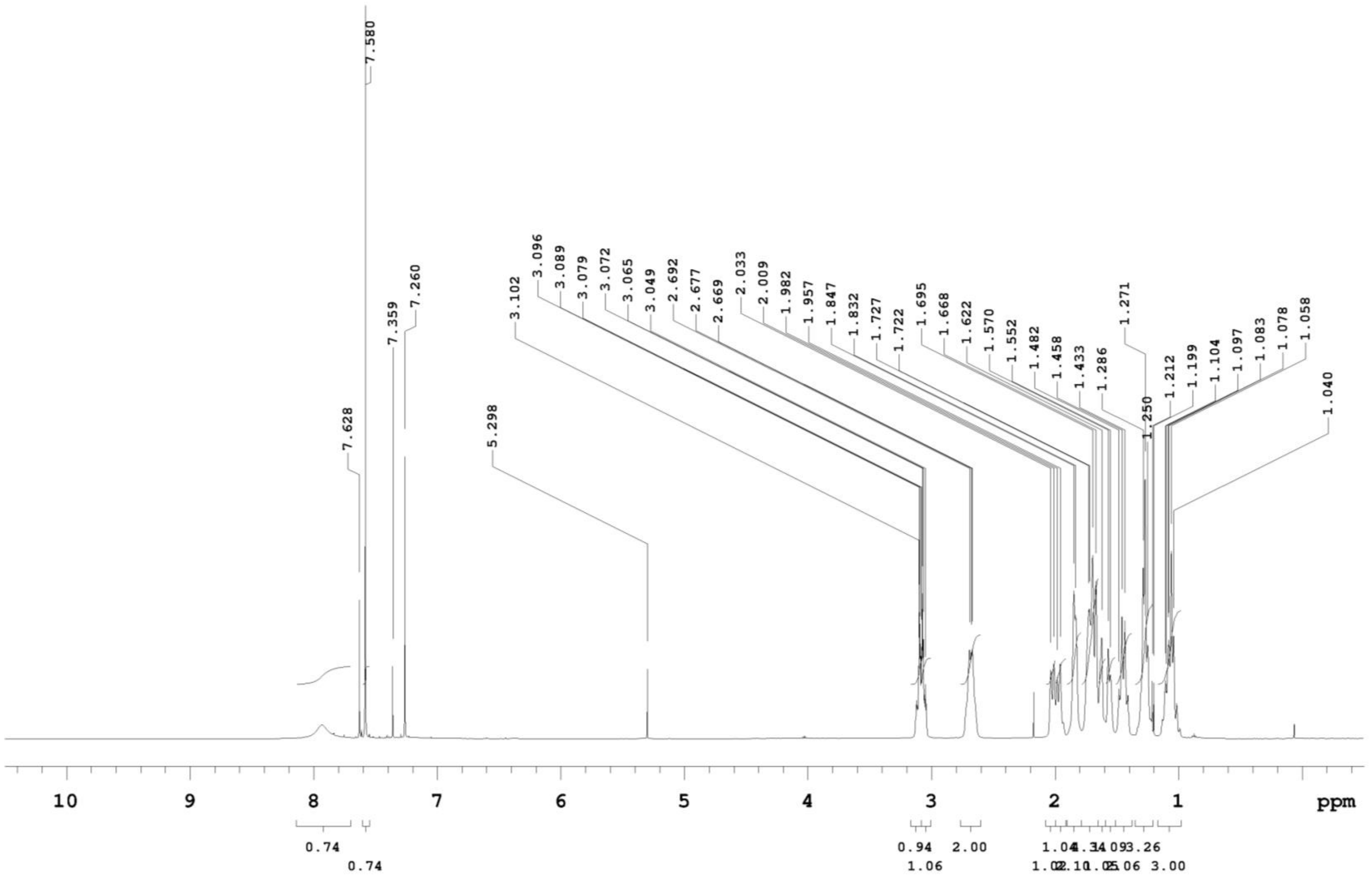

(*R*)-2,4,6-Tribromo-*N*,*N*-dicyclohexyl-3-hydroxybenzamide (2d)

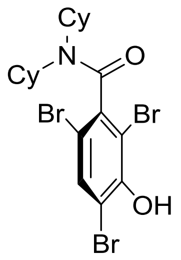

^{13}C NMR

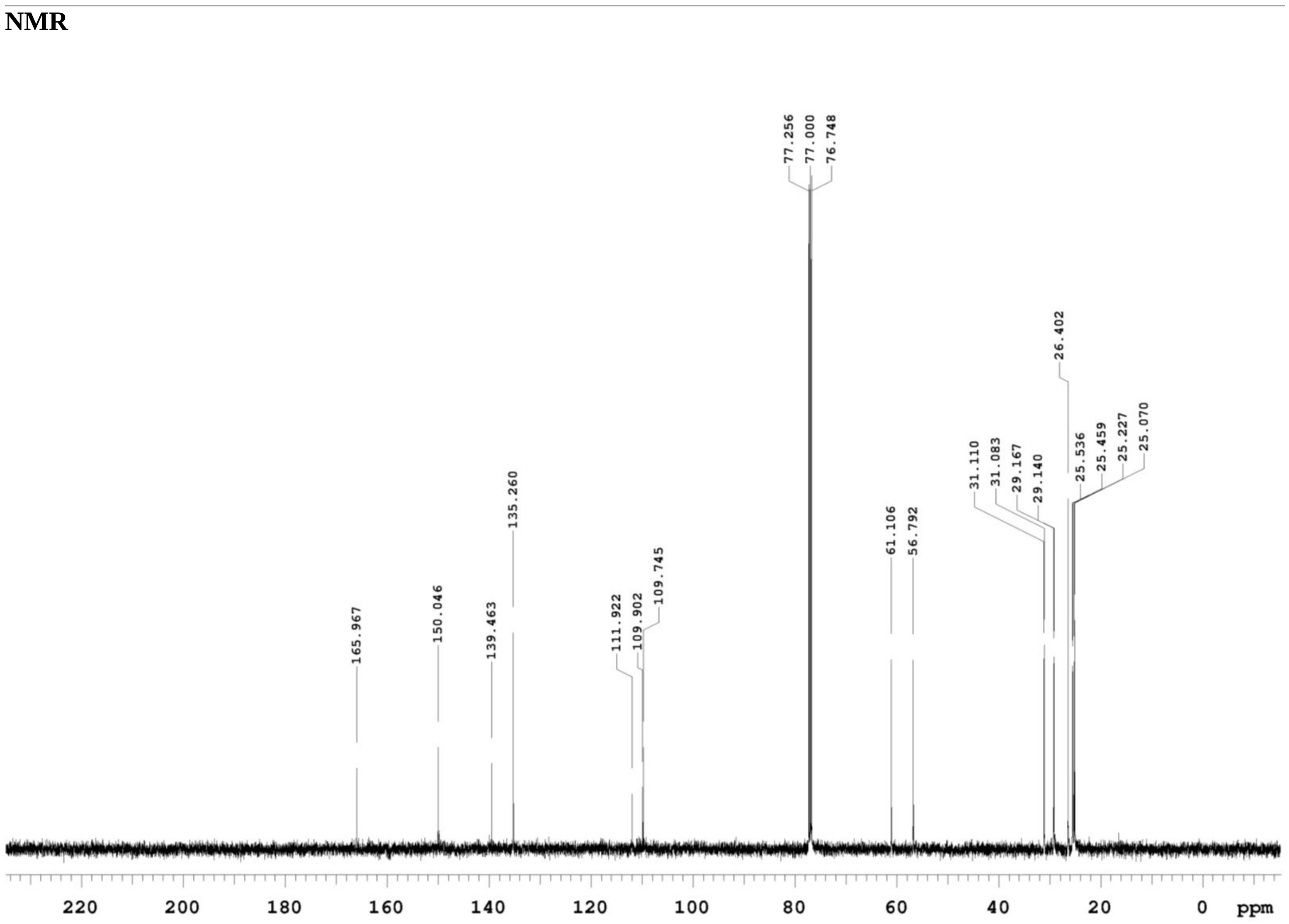

Piperidin-1-yl(2,4,6-tribromo-3-hydroxyphenyl)methanone (2e)

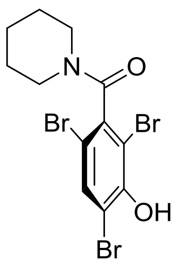

^{1}H NMR

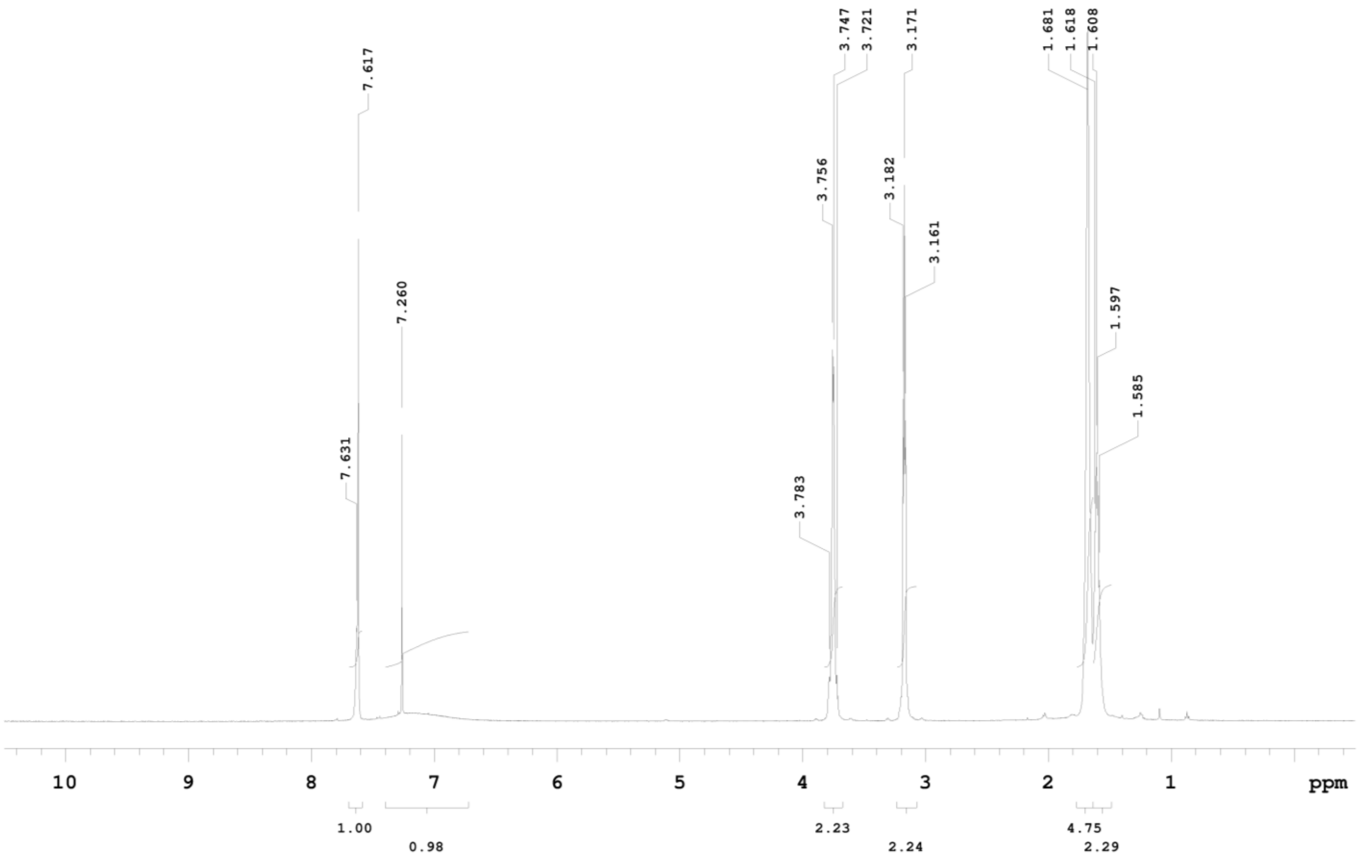

Piperidin-1-yl(2,4,6-tribromo-3-hydroxyphenyl)methanone (2e)

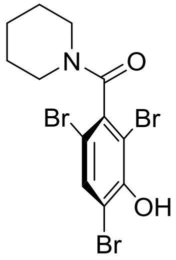

^{13}C NMR

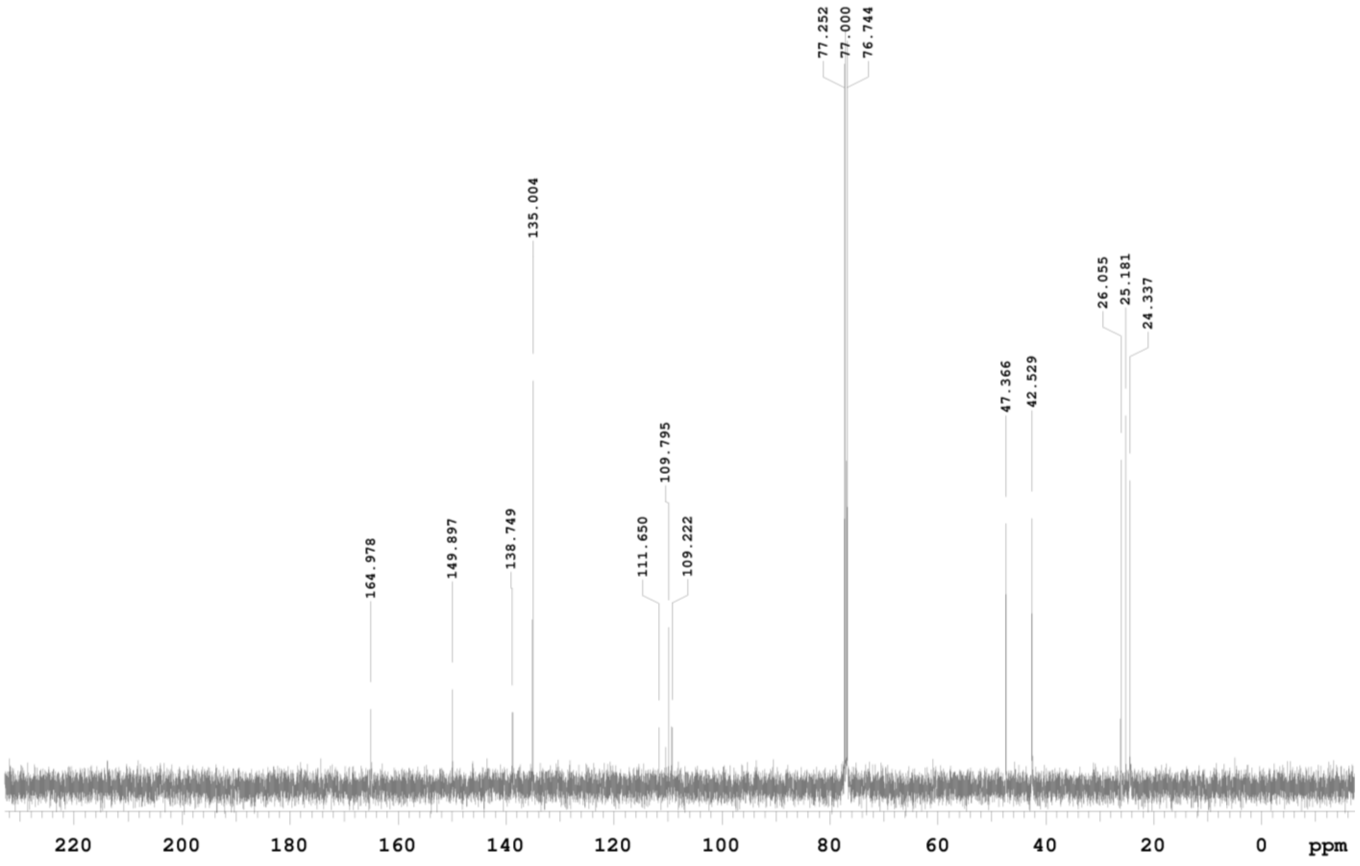

2,4,6-Tribromo-5-hydroxy-*N*,*N*-diisopropyl-[1,1'-biphenyl]-3-carboxamide (2f)

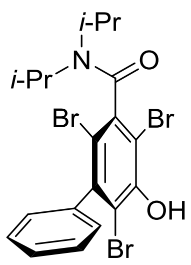

^{1}H NMR

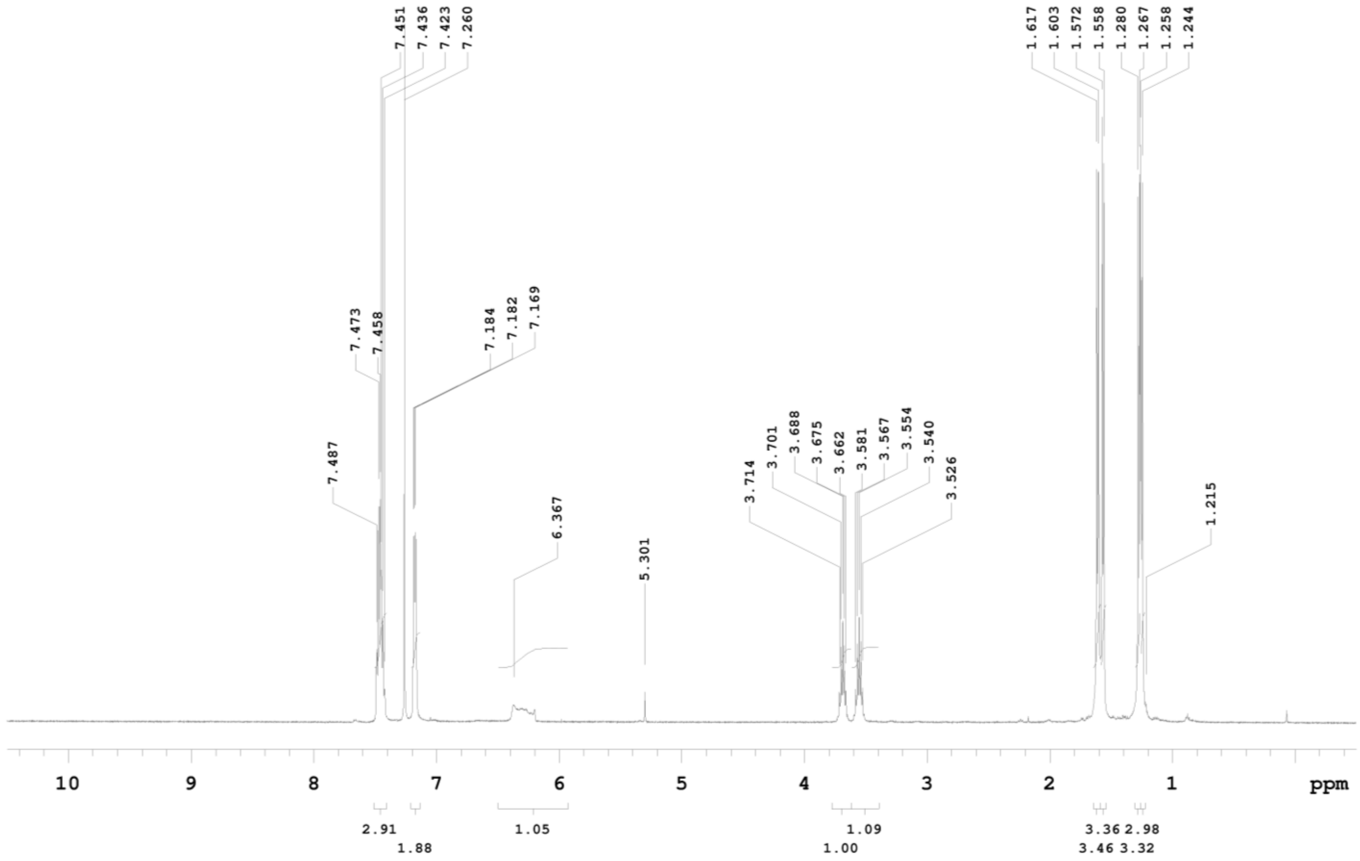

2,4,6-Tribromo-5-hydroxy-*N*,*N*-diisopropyl-[1,1'-biphenyl]-3-carboxamide (2f)

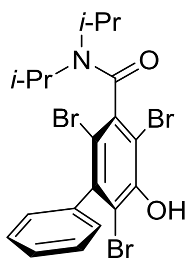

^{13}C NMR

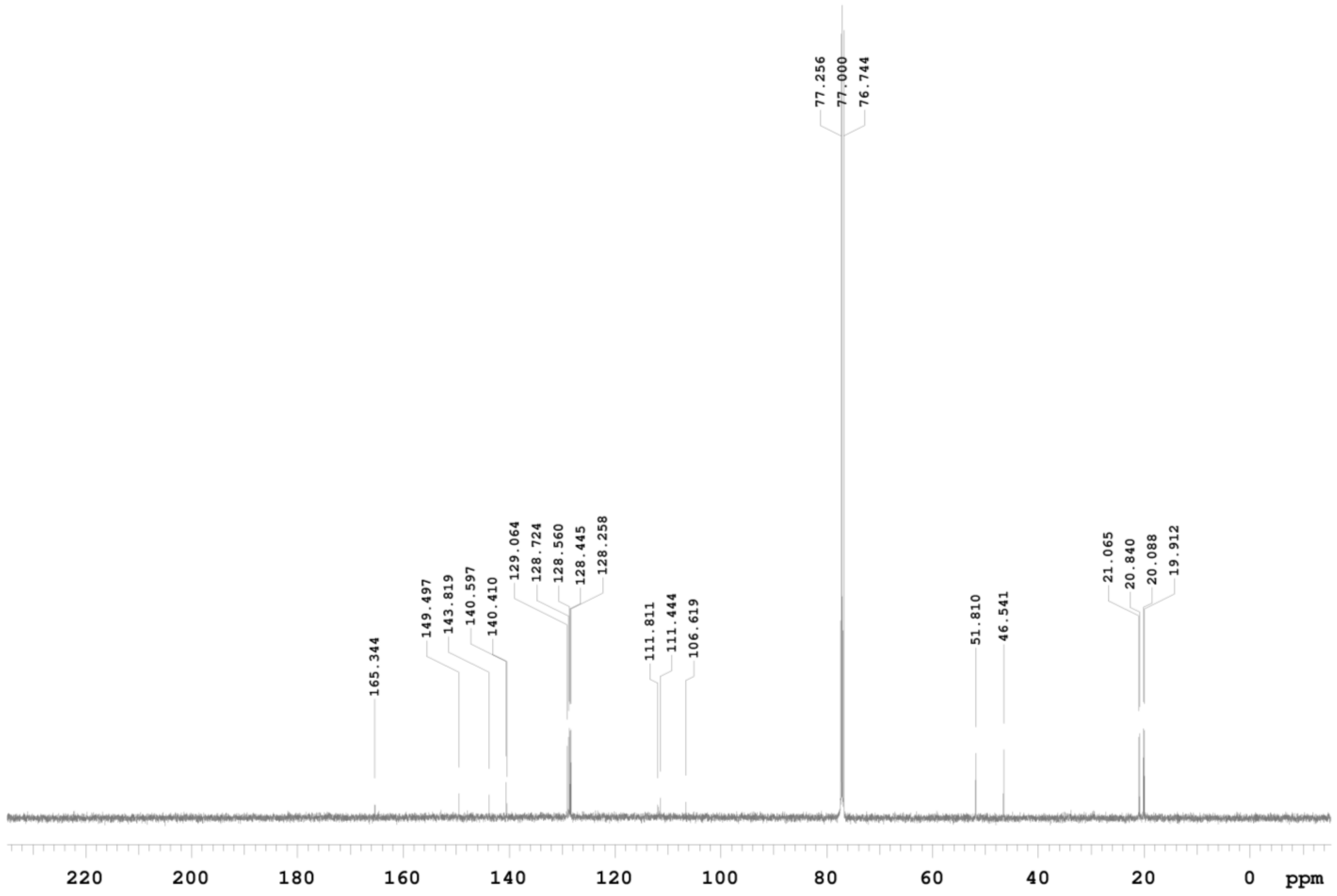

2,4,6-Tribromo-5-hydroxy-*N*,*N*-diisopropyl-4'-methoxy-[1,1'-biphenyl]-3-carboxamide (2g)

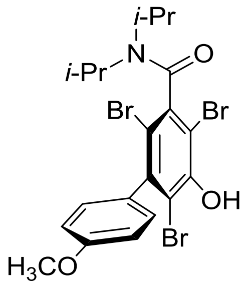

^{1}H NMR

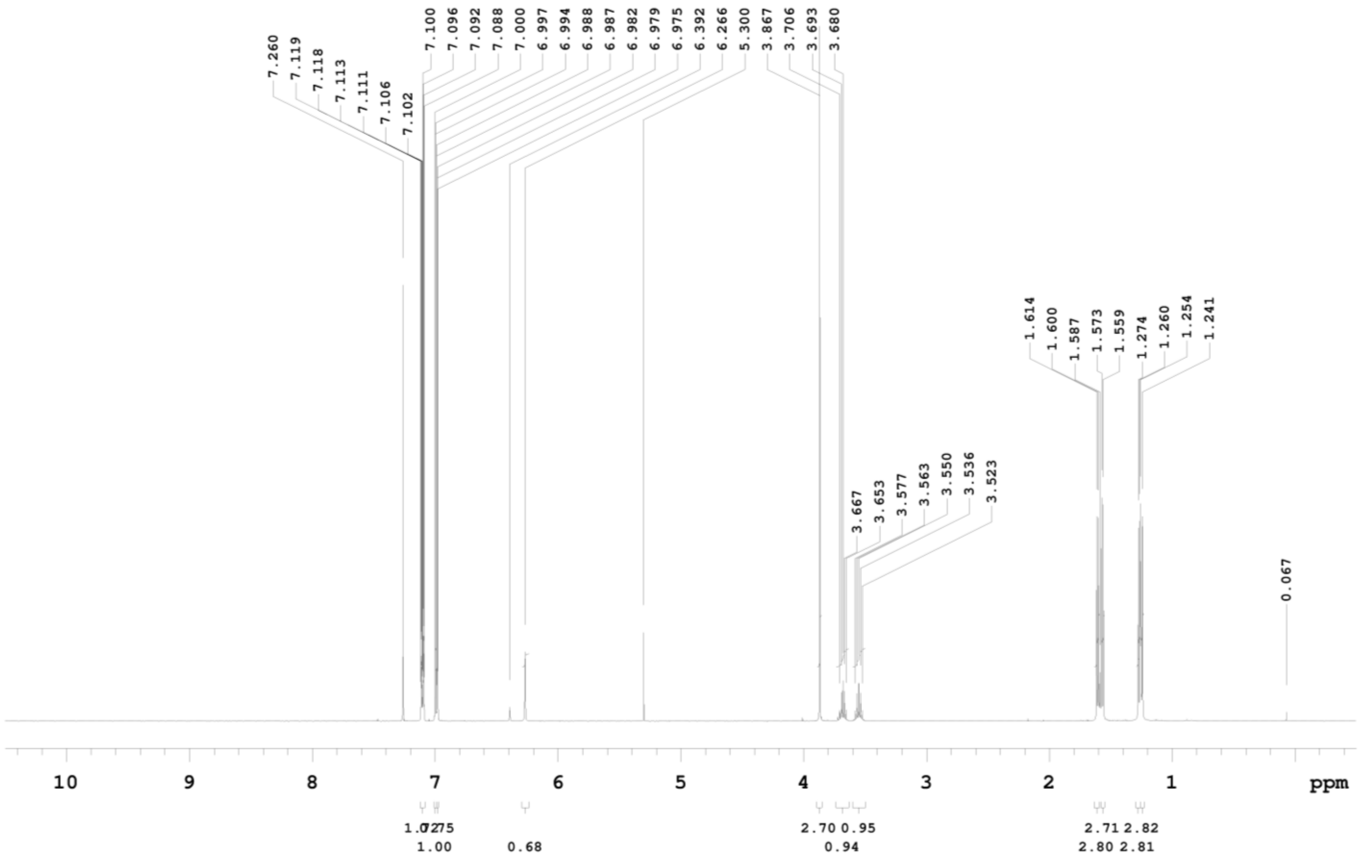

2,4,6-Tribromo-5-hydroxy-*N*,*N*-diisopropyl-4'-methoxy-[1,1'-biphenyl]-3-carboxamide (2g)

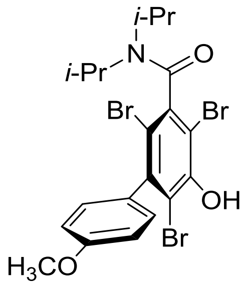

^{13}C NMR

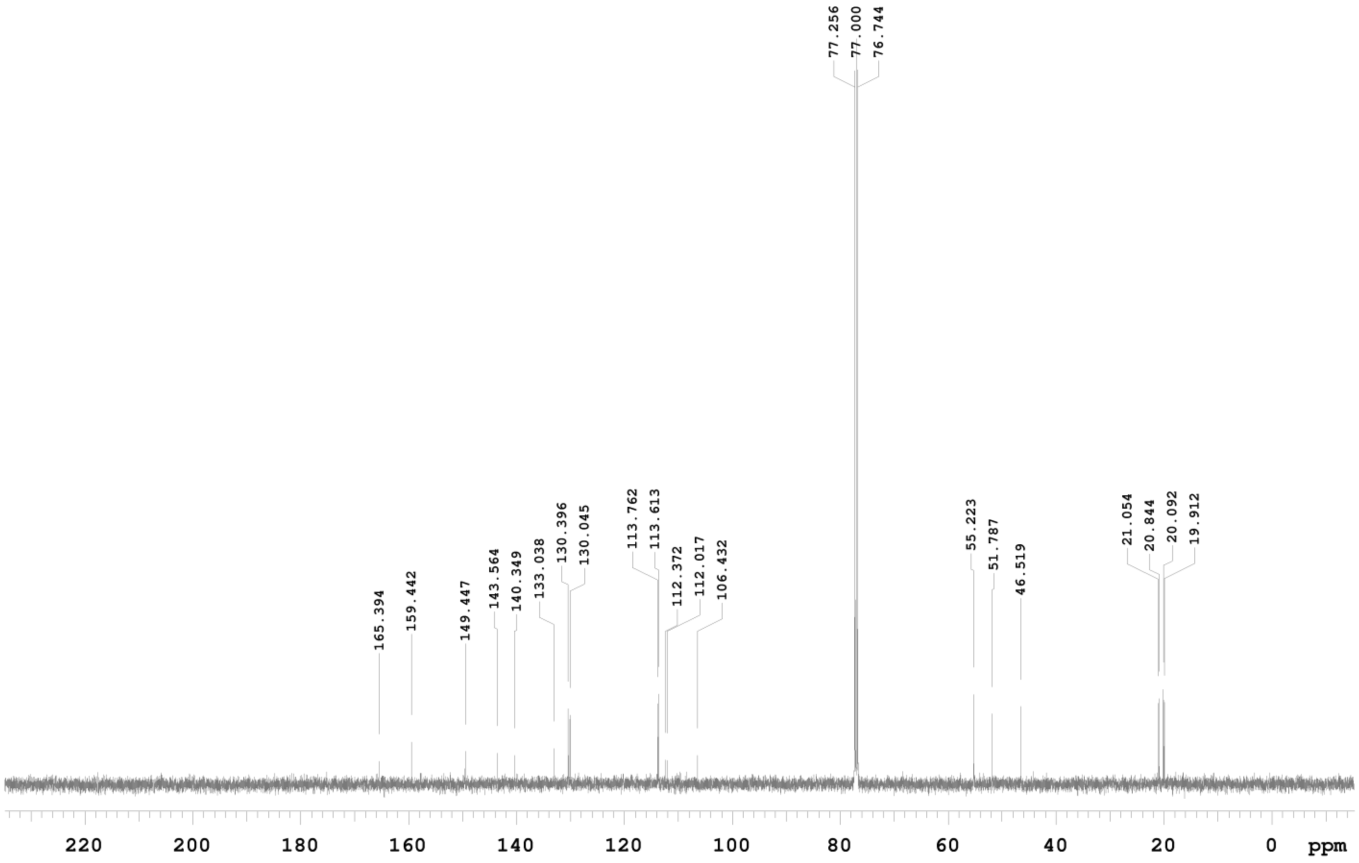

2,4,6-Tribromo-5-hydroxy-*N*,*N*-diisopropyl-4'-(trifluoromethyl)-[1,1'-biphenyl]-3-carboxamide (2h)

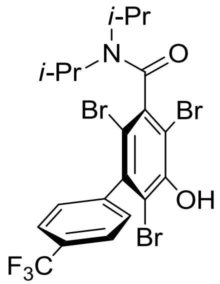

^{1}H NMR

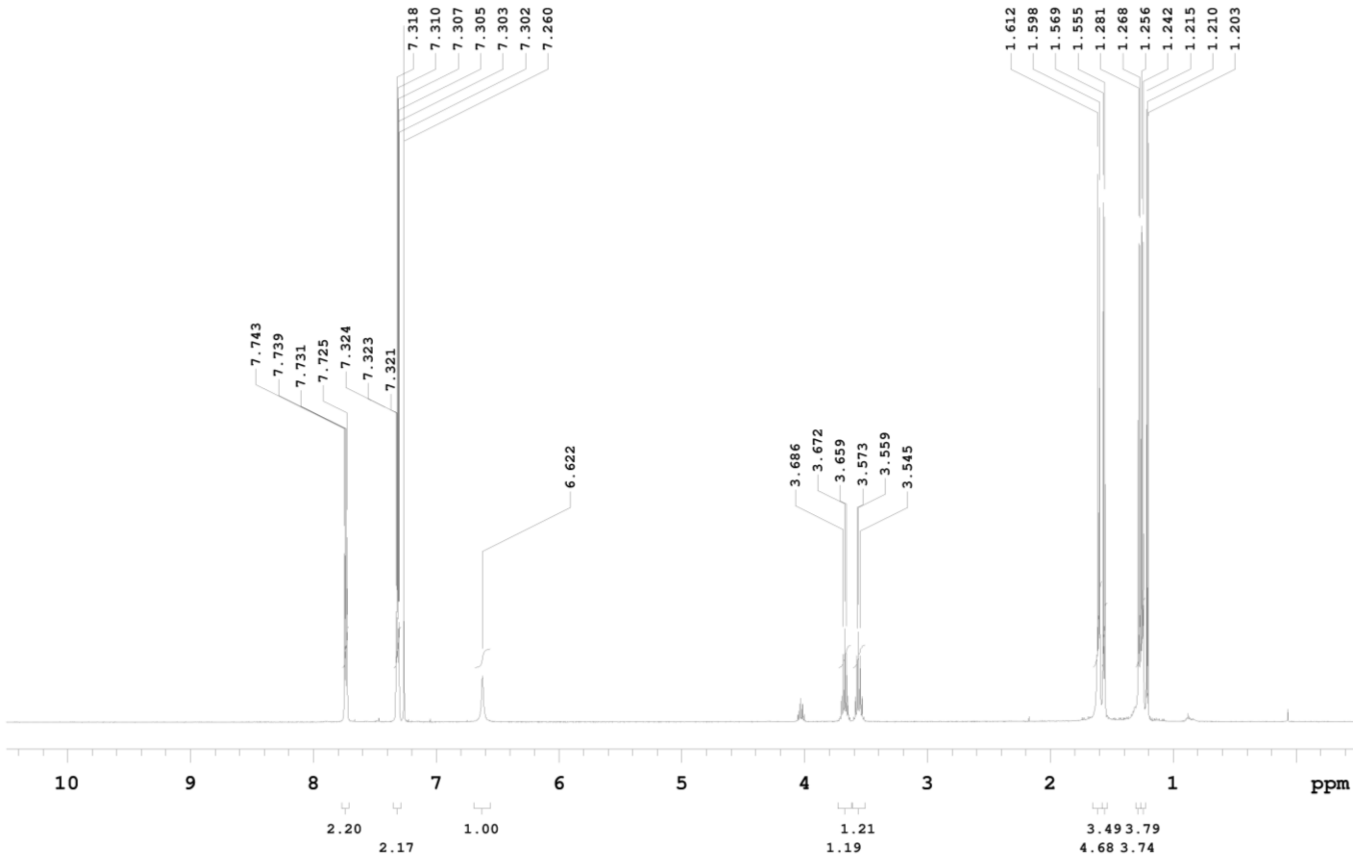

2,4,6-Tribromo-5-hydroxy-*N*,*N*-diisopropyl-4'-(trifluoromethyl)-[1,1'-biphenyl]-3-carboxamide (2h)

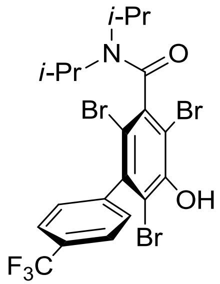

^{13}C NMR

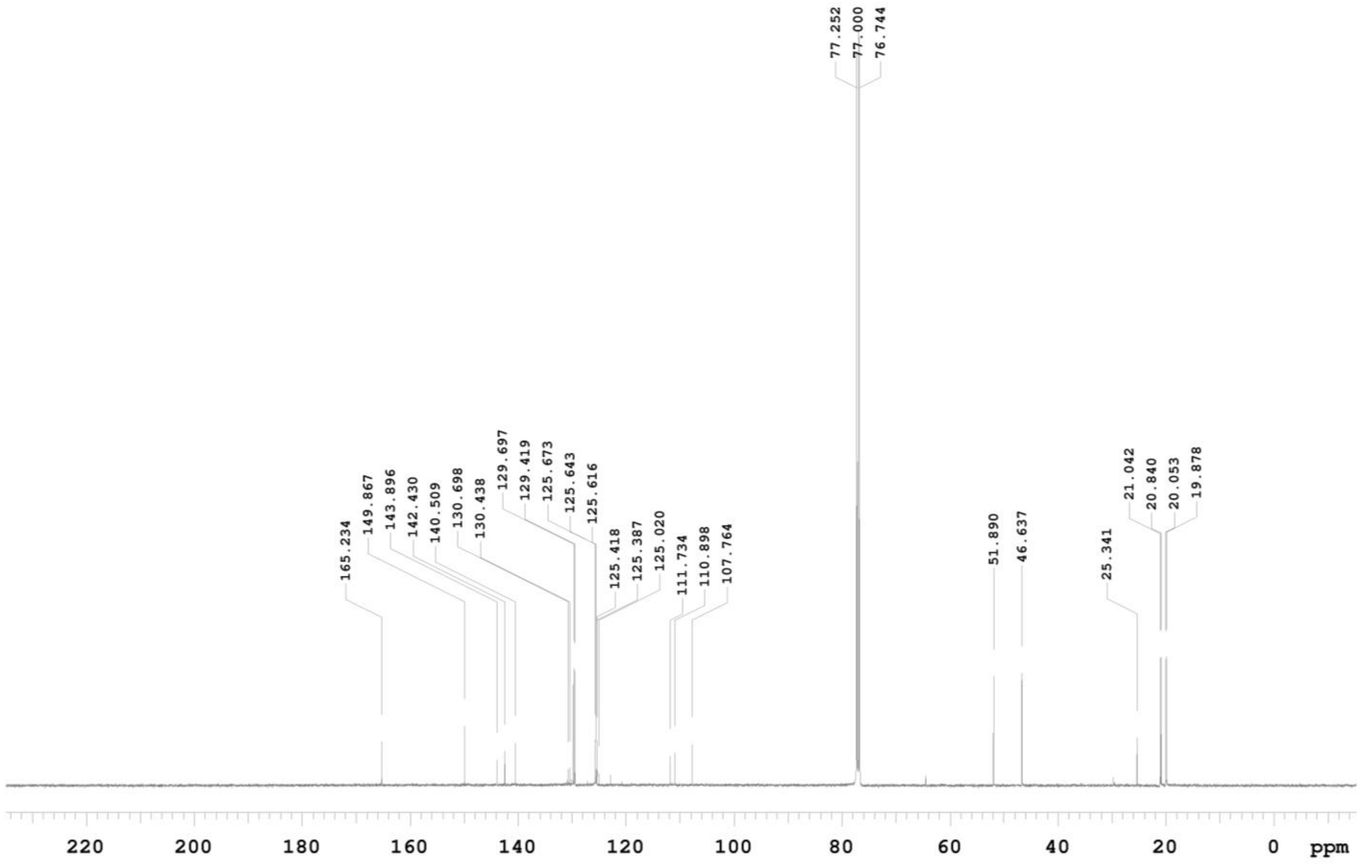

2,4,6-Tribromo-3-hydroxy-*N*,*N*-diisopropyl-5-(naphthalen-2-yl)benzamide (2i)

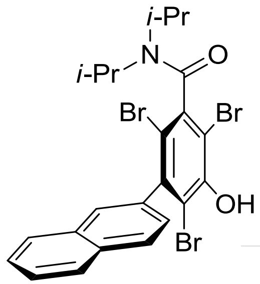

^{1}H NMR

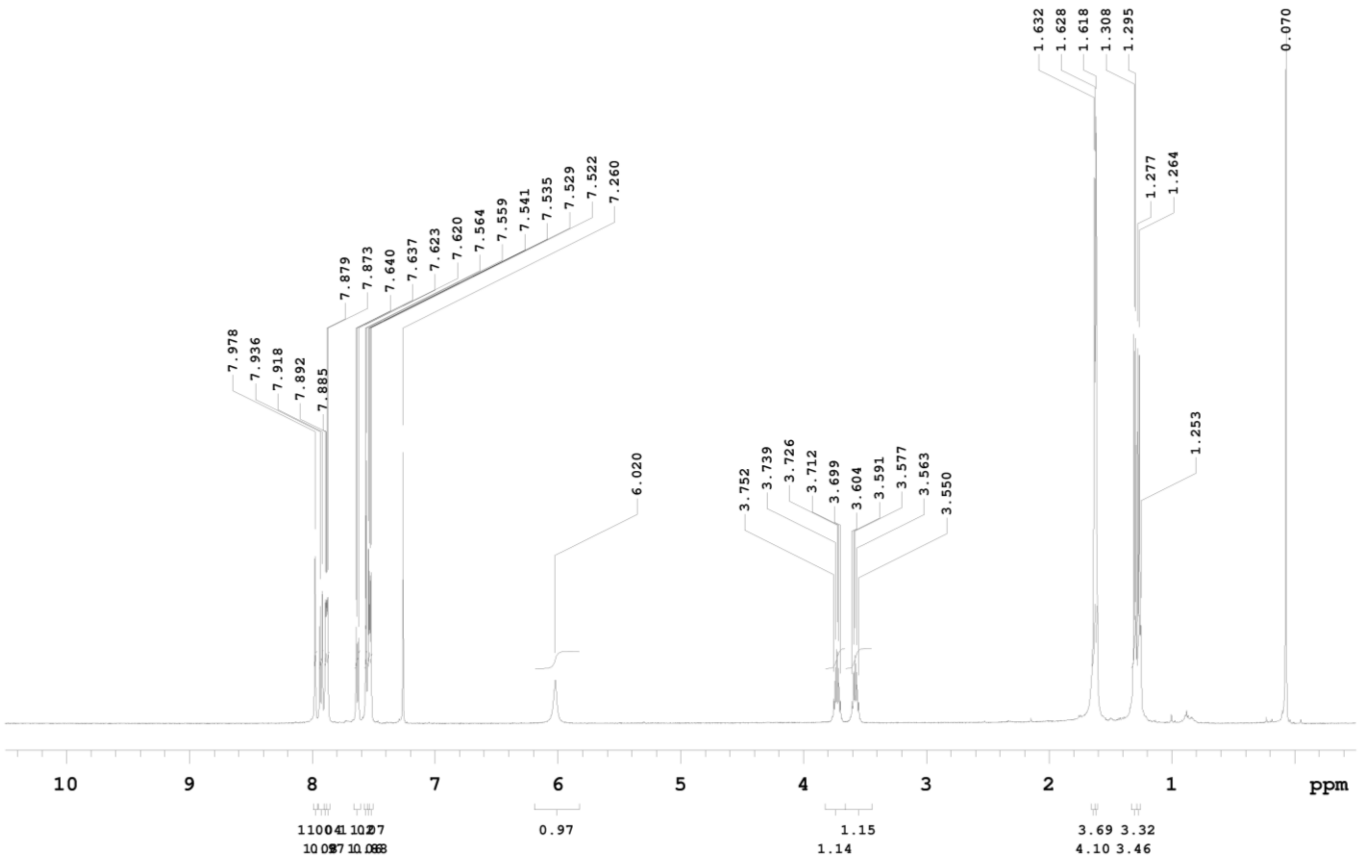

2,4,6-Tribromo-3-hydroxy-*N*,*N*-diisopropyl-5-(naphthalen-2-yl)benzamide (2i)

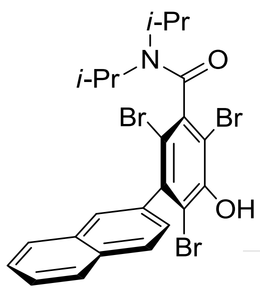

^{13}C NMR

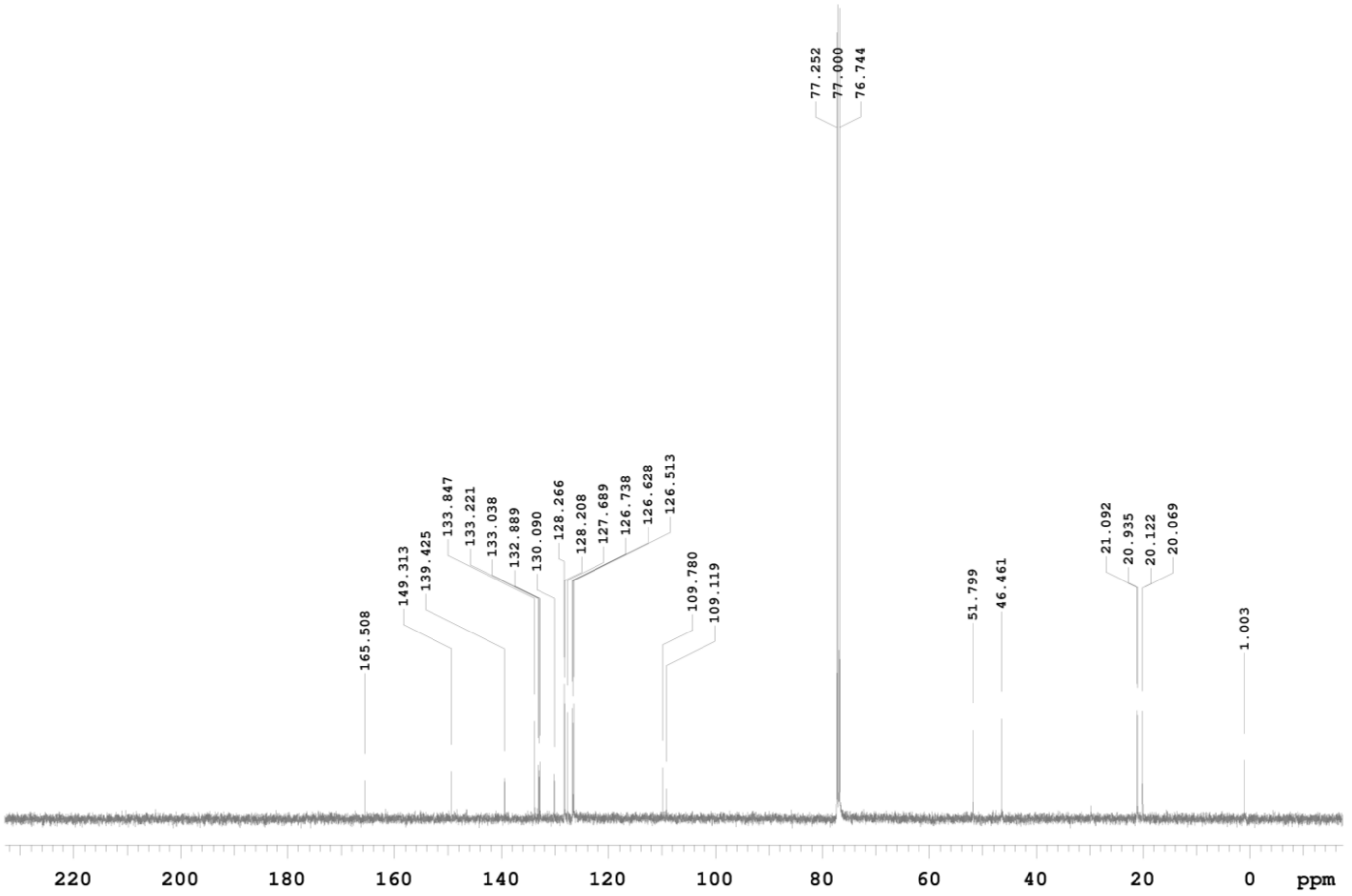

2,4,6-Tribromo-3-cyclopropyl-5-hydroxy-*N*,*N*-diisopropylbenzamide (2j)

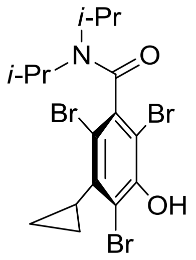

^{1}H NMR

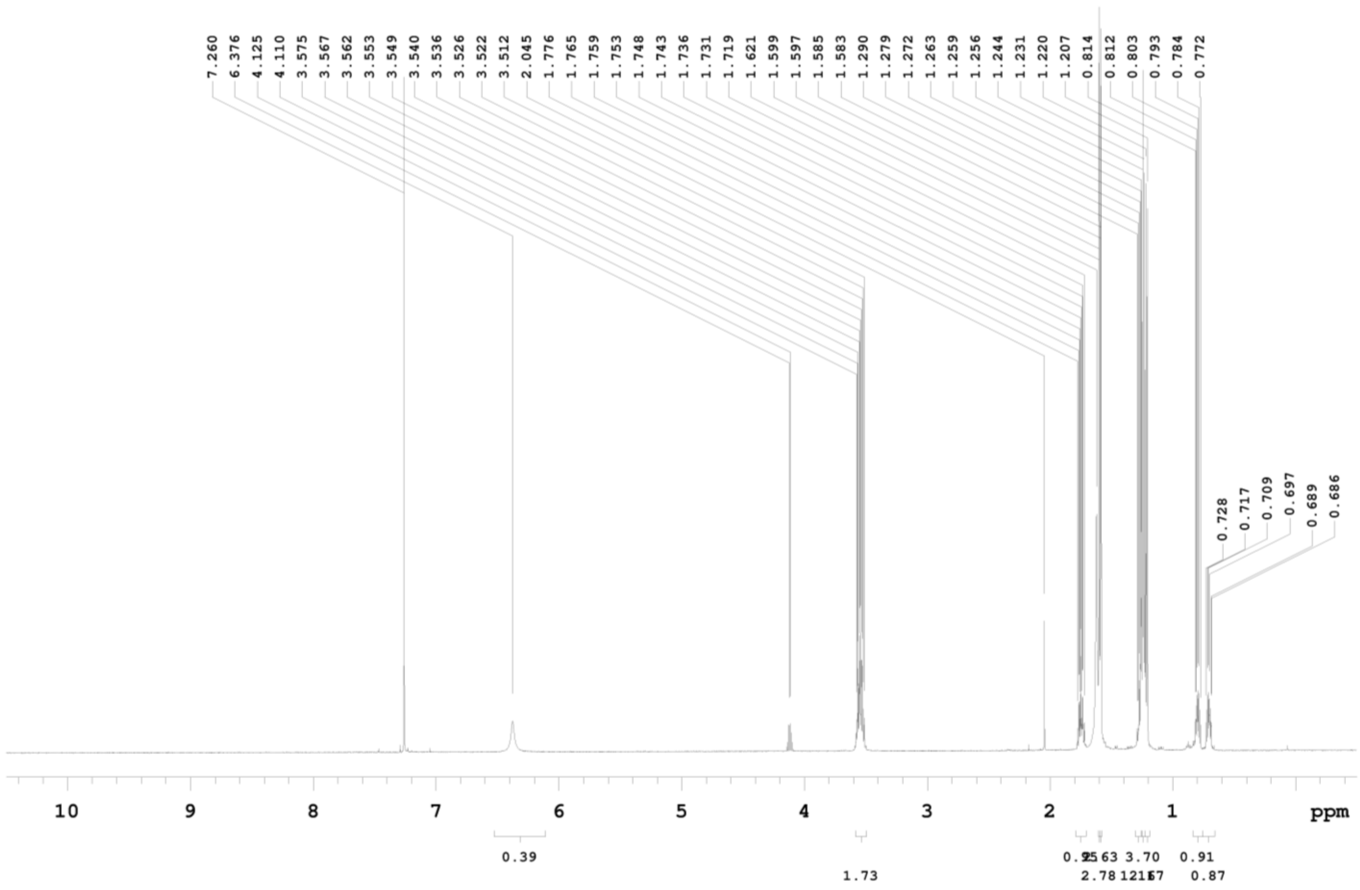

2,4,6-Tribromo-3-cyclopropyl-5-hydroxy-*N*,*N*-diisopropylbenzamide (2j)

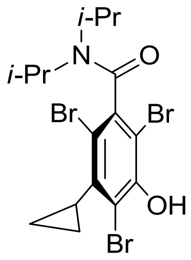

^{13}C NMR

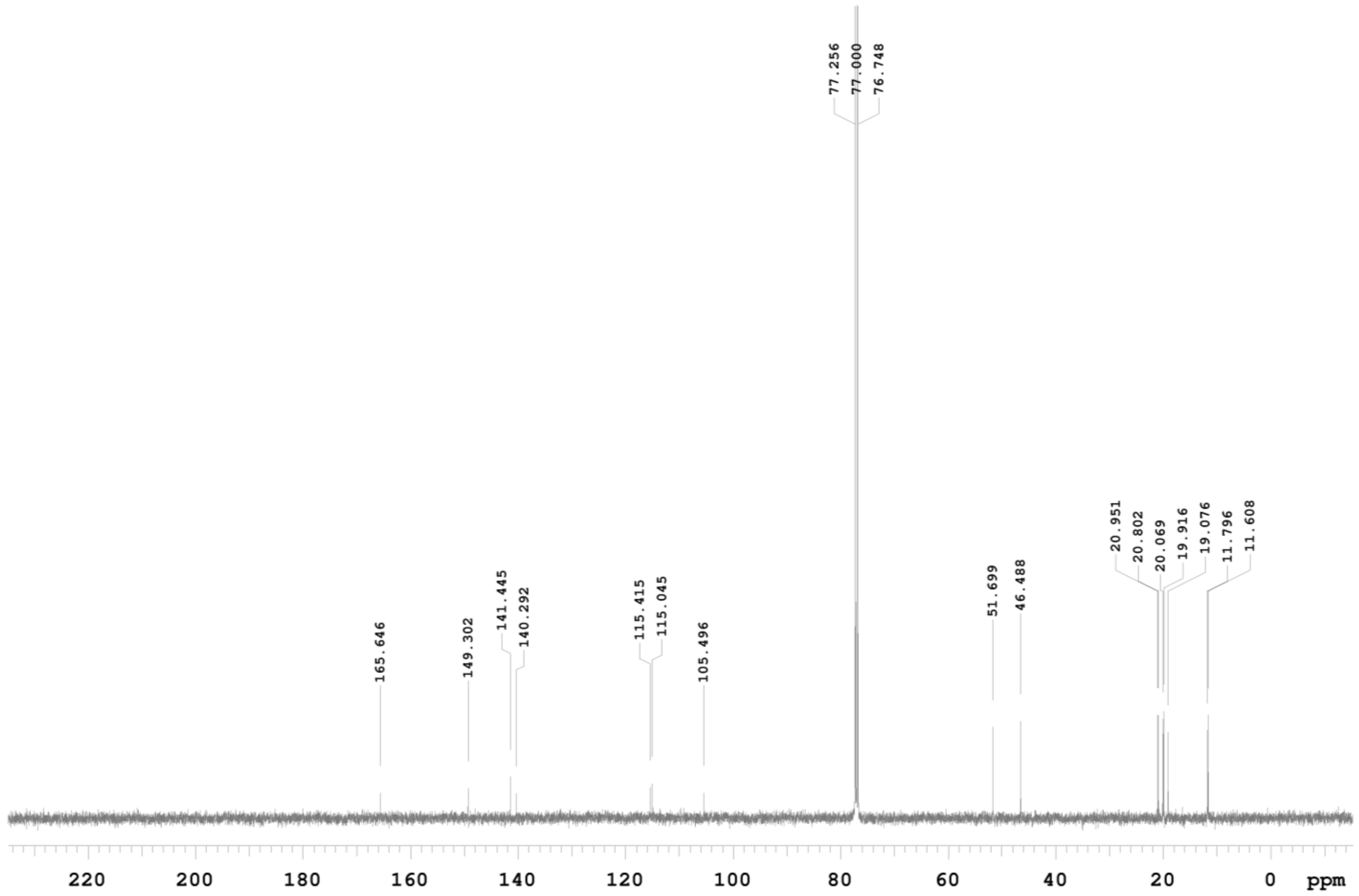

3,5-Dibromo-2-hydroxy-*N*,*N*-diisopropyl-[1,1'-biphenyl]-4-carboxamide (2k)

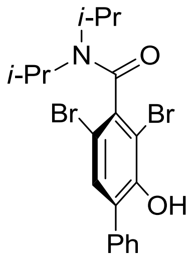

^{1}H NMR

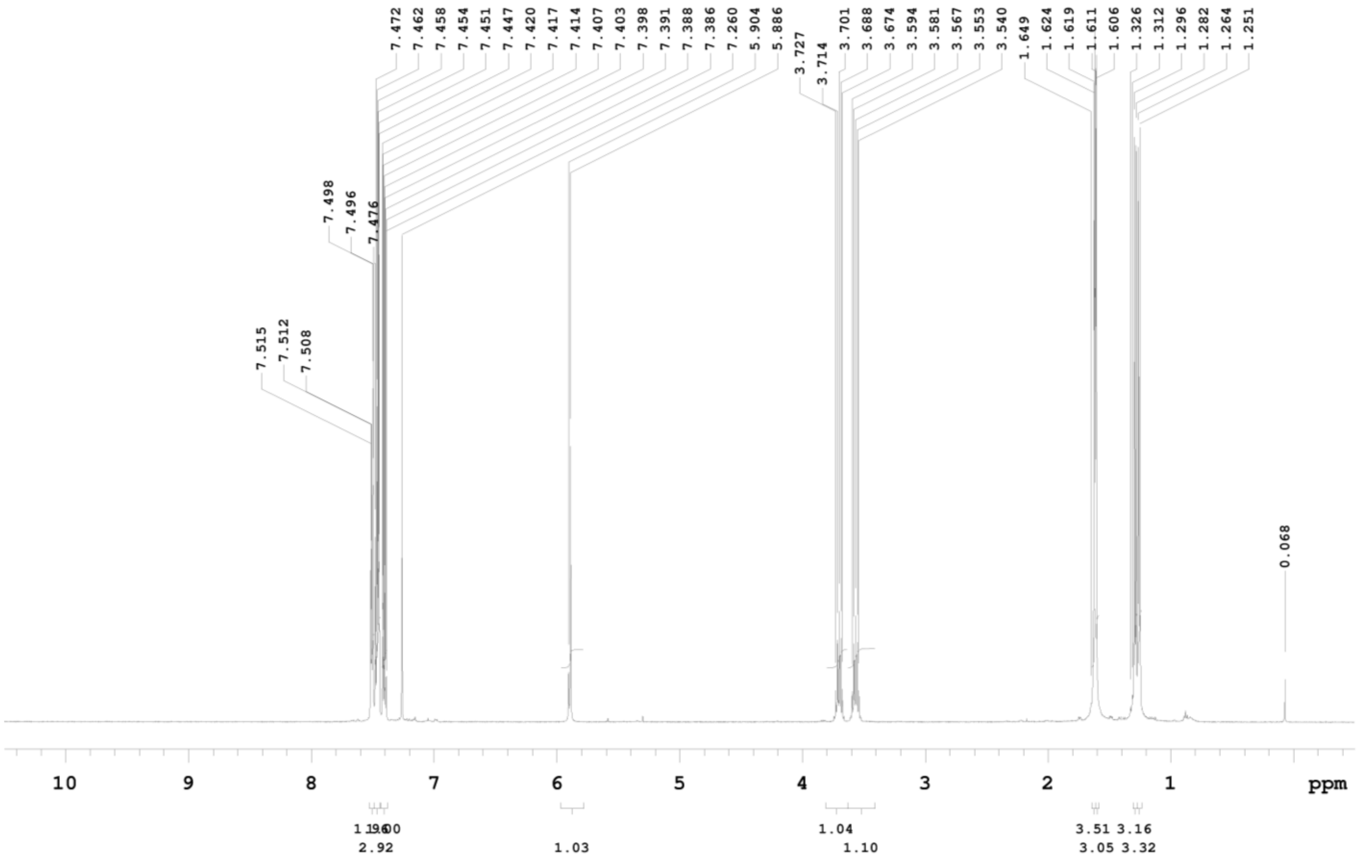

3,5-Dibromo-2-hydroxy-*N*,*N*-diisopropyl-[1,1'-biphenyl]-4-carboxamide (2k)

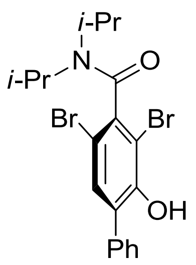

^{13}C NMR

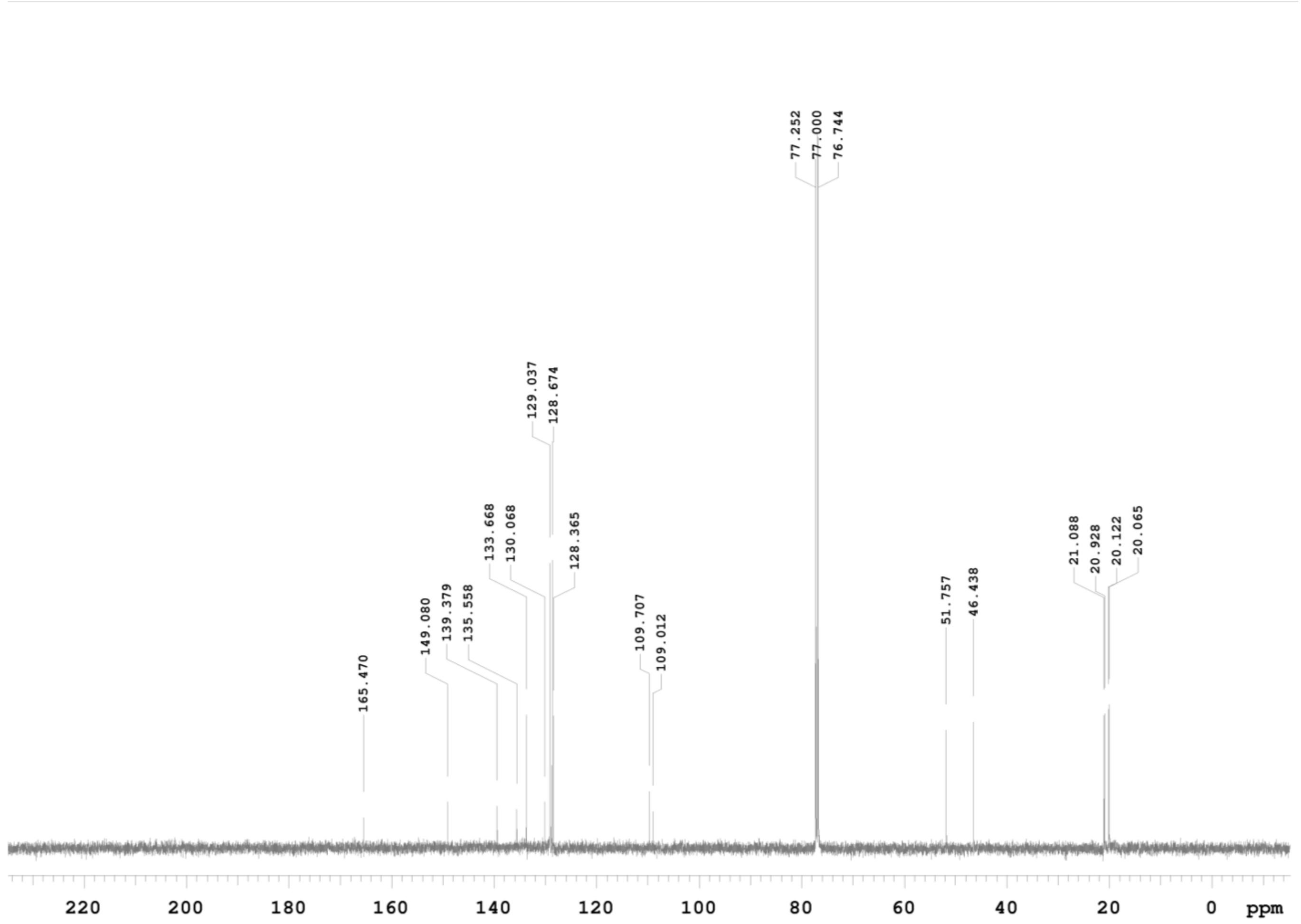

2,6-Dibromo-4-cyclopropyl-3-hydroxy-*N*,*N*-diisopropylbenzamide (2l)

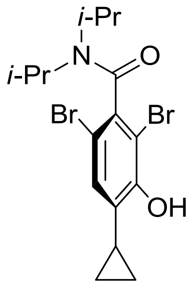

^{1}H NMR

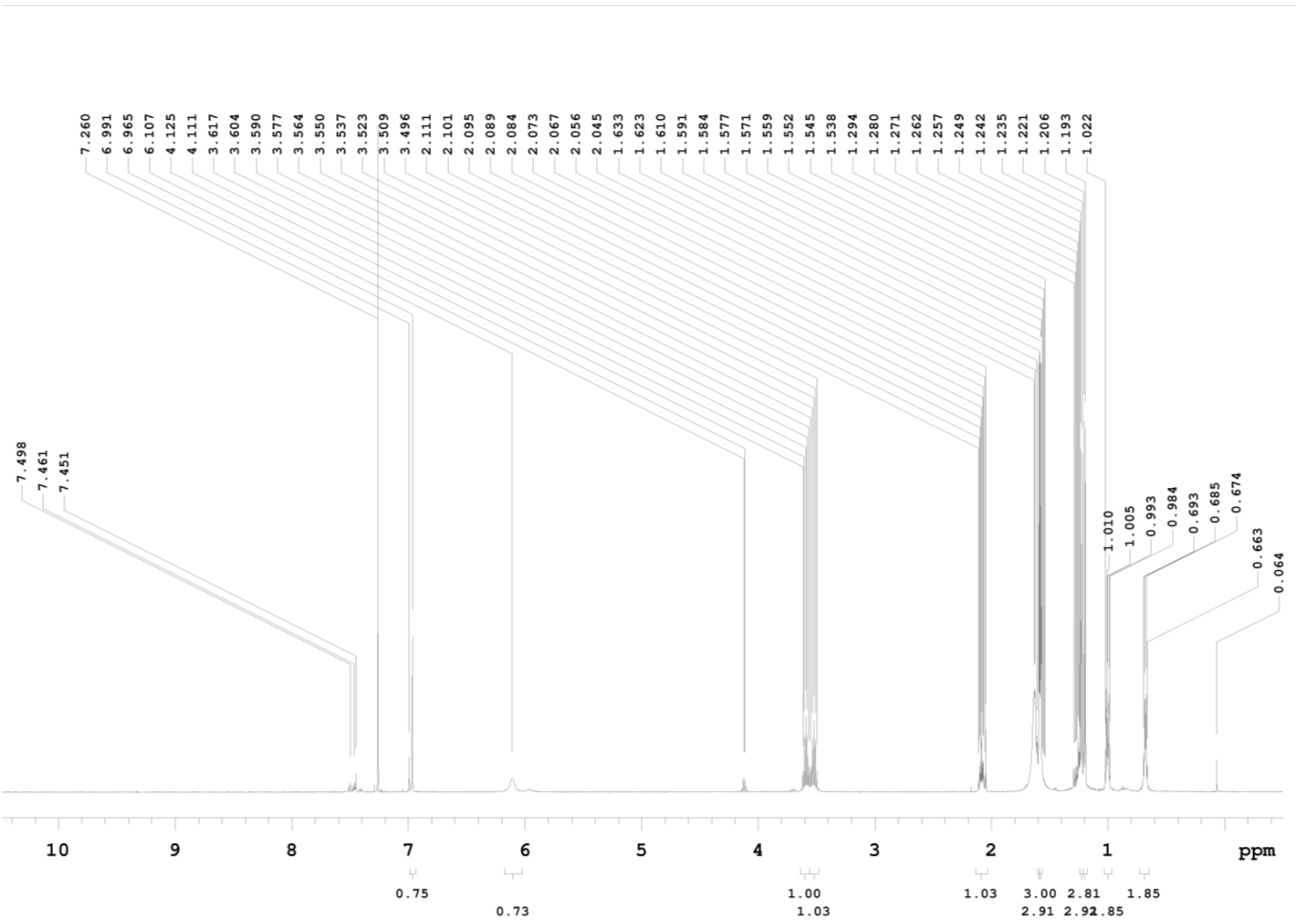

2,6-Dibromo-4-cyclopropyl-3-hydroxy-*N*,*N*-diisopropylbenzamide (2l)

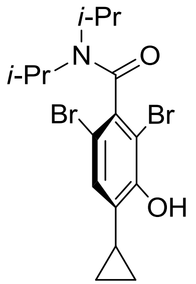

^{13}C NMR

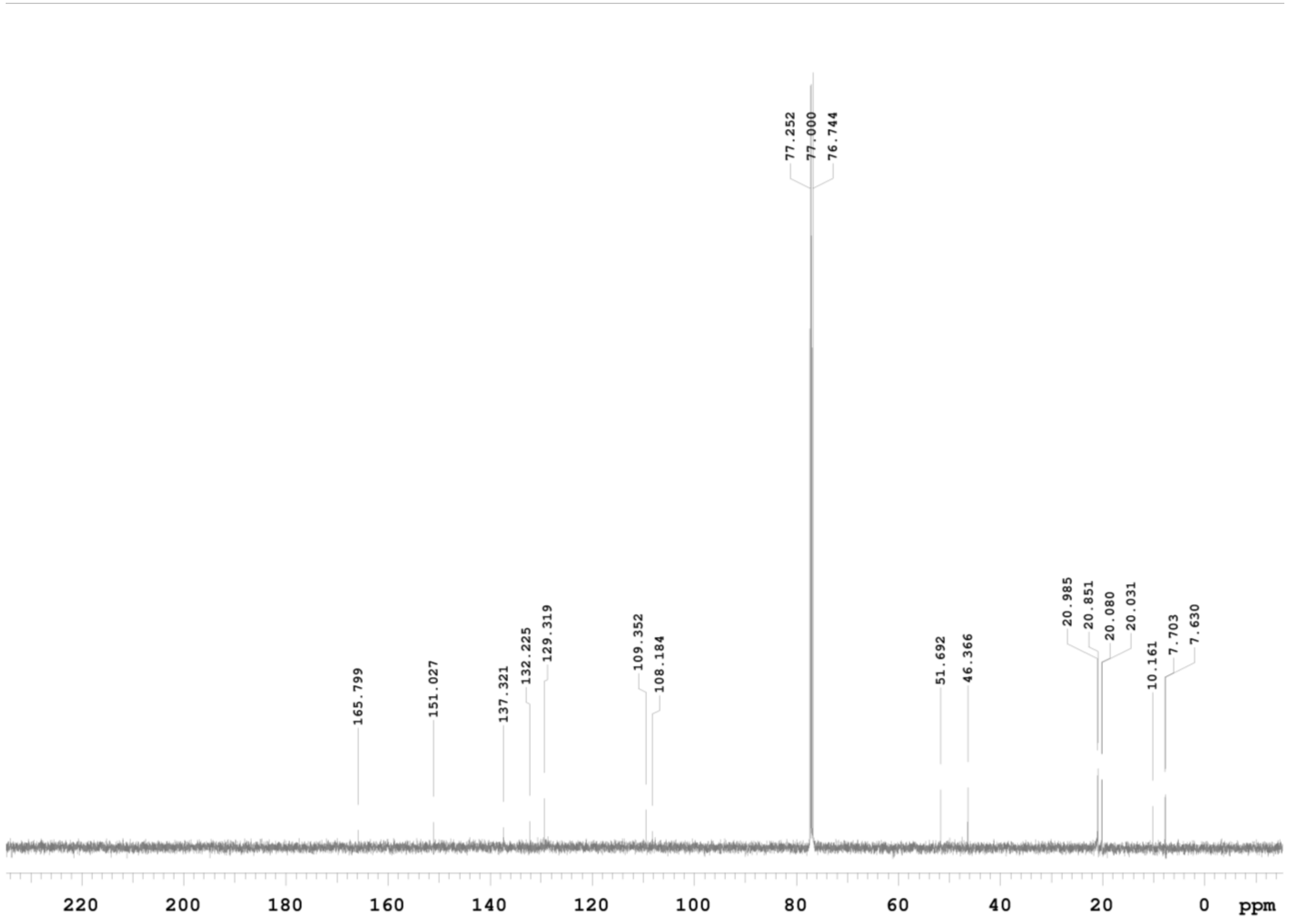

HPLC Chromatogram Profiles

2,4,6-Tribromo-3-hydroxy-*N*,*N*-diisopropylbenzamide (2a).

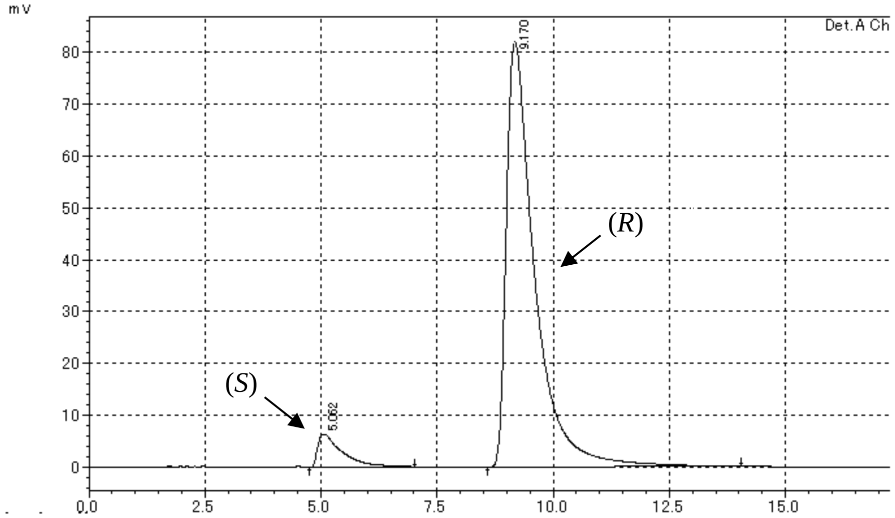

peak#	retention time (min)	area	height	area%
1	5.062	235346	6414	6.313
2	9.170	3492768	82060	93.687
total				100.000

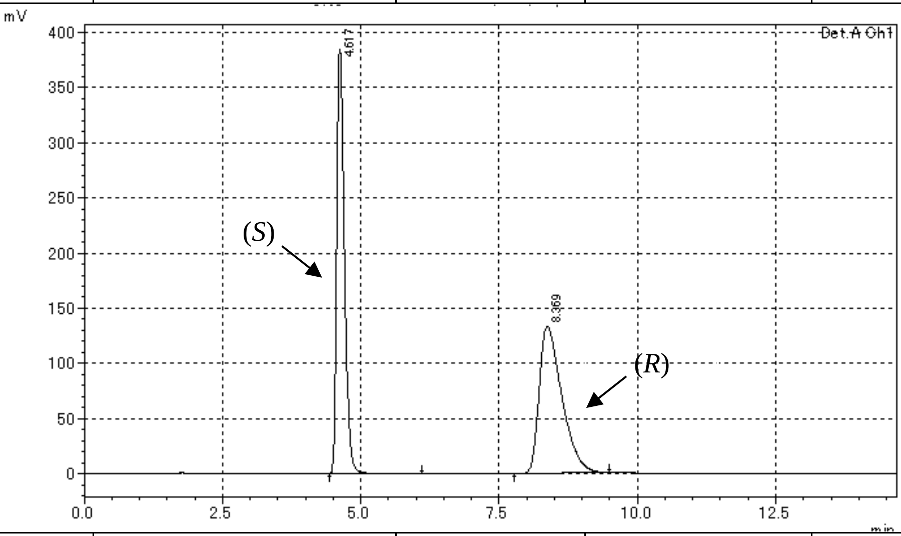

peak#	retention time (min)	area	height	area%
1	4.617	3739727	384899	49.537
2	8.369	3809588	133452	50.463
total				100.000

2,4,6-Tribromo-3-hydroxy-*N*,*N*-dimethylbenzamide (2b).

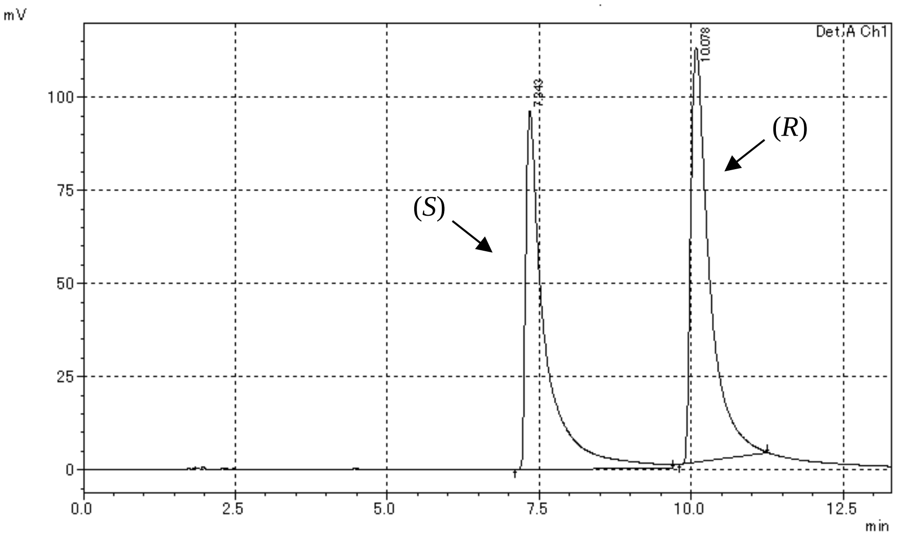

peak#	retention time (min)	area	height	area%
1	7.343	2120057	96216	46.970
2	10.078	2393627	111348	53.030
total				100.000

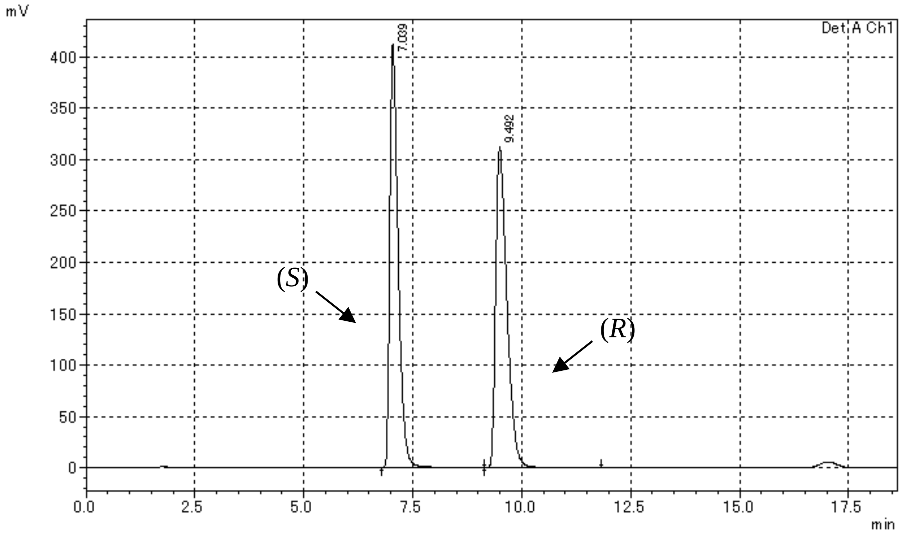

peak#	retention time (min)	area	height	area%
1	7.039	5553753	412463	49.828
2	9.492	5592137	311902	50.172
total				100.000

2,4,6-Tribromo-3-hydroxy-*N*,*N*-diisobutylbenzamide (2c).

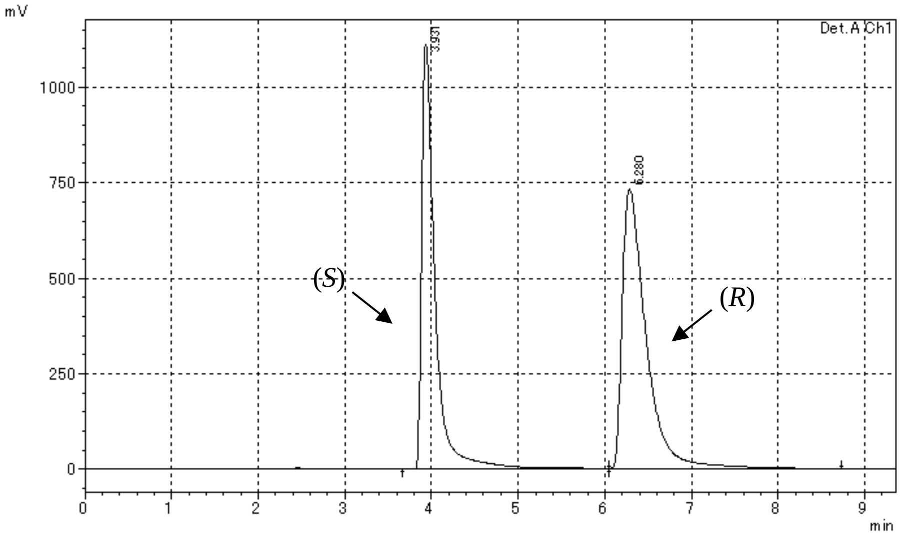

peak#	retention time (min)	area	height	area%
1	3.931	11998822	1112669	46.144
2	6.280	14004104	732252	53.856
total				100.000

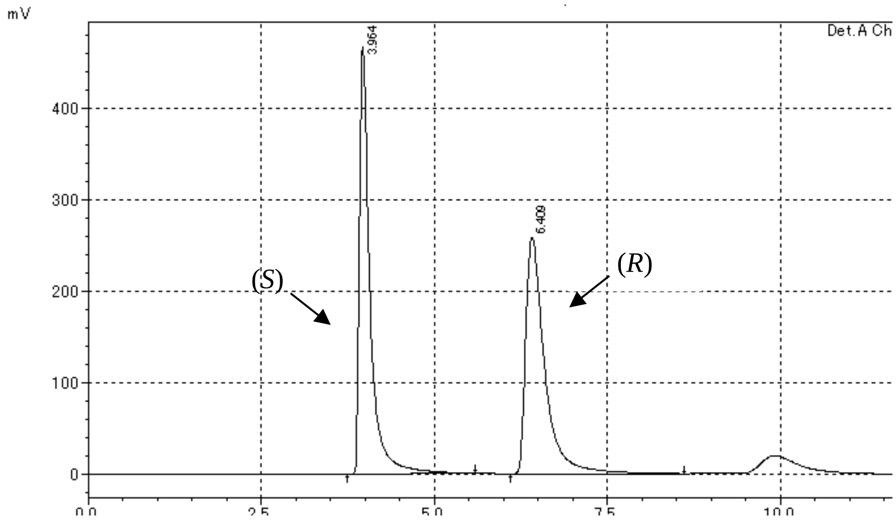

peak#	retention time (min)	area	height	area%
1	3.964	5052196	467143	50.589
2	6.409	4934474	258041	49.411
total				100.000

(*R*)-2,4,6-Tribromo-*N*,*N*-dicyclohexyl-3-hydroxybenzamide (2d).

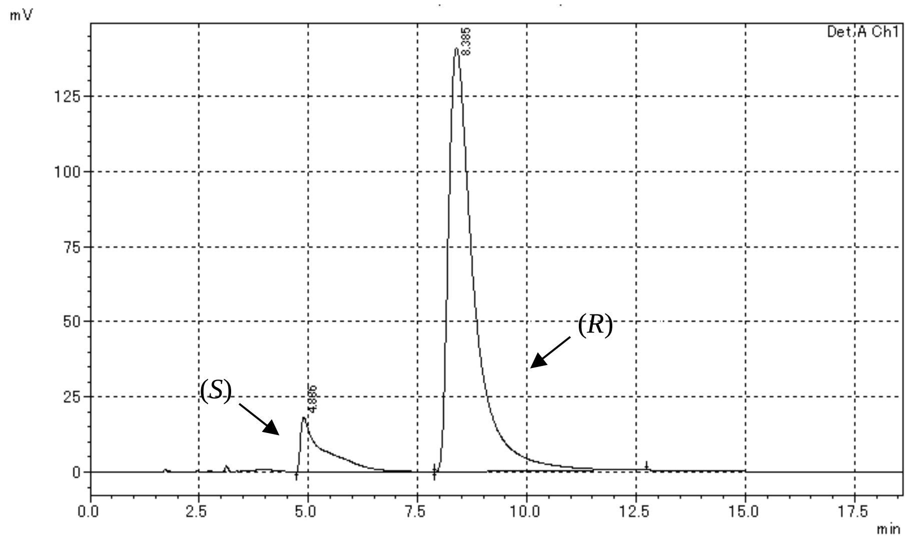

peak#	retention time (min)	area	height	area%
1	4.886	676618	17996	10.595
2	8.385	5709369	140995	89.405
total				100.000

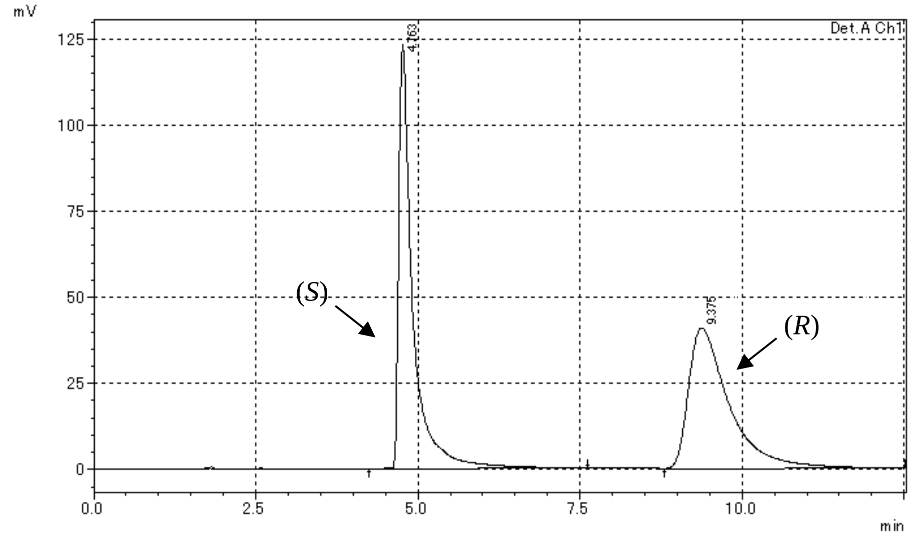

peak#	retention time (min)	area	height	area%
1	4.763	1872657	123423	51.222
2	9.375	1783317	40816	48.778
total				100.000

Piperidin-1-yl(2,4,6-tribromo-3-hydroxyphenyl)methanone (2e).

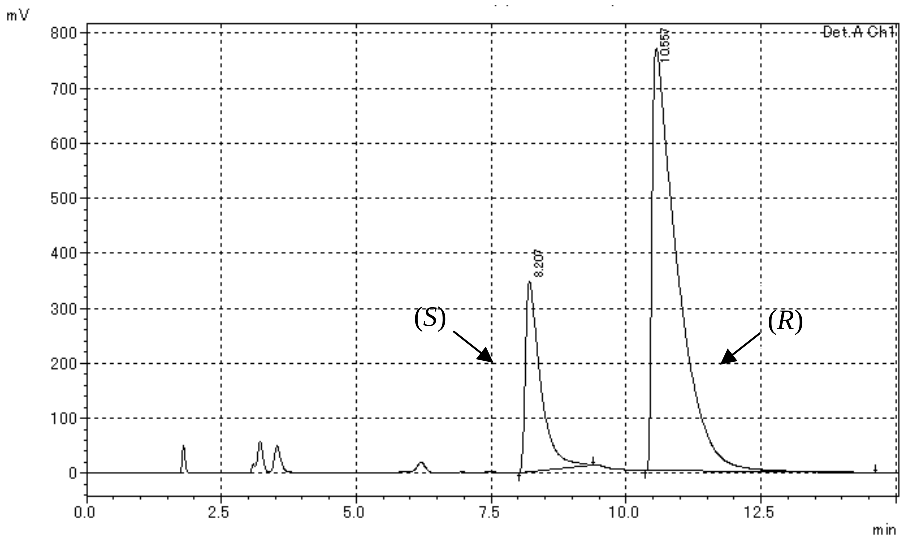

peak#	retention time (min)	area	height	area%
1	8.207	6799915	346775	21.466
2	10.557	24877176	768385	78.534
total				100.000

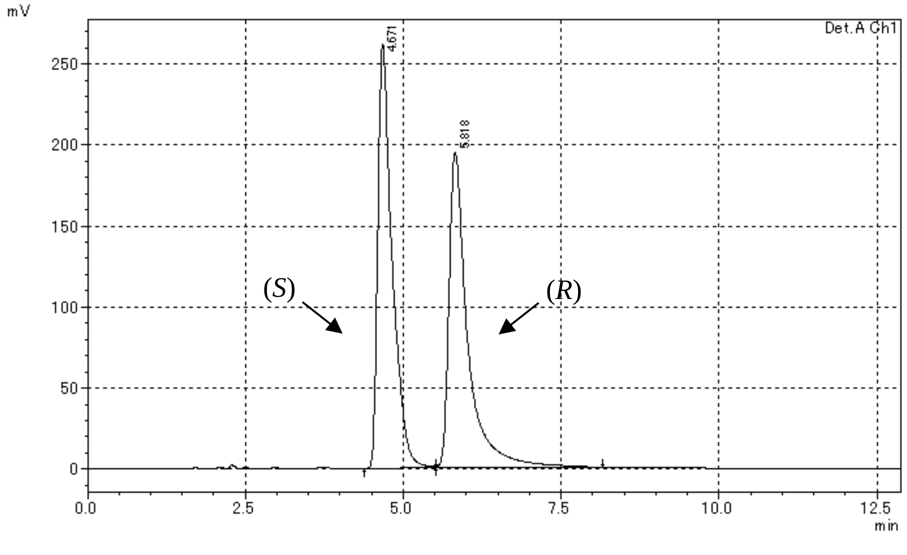

peak#	retention time (min)	area	height	area%
1	4.671	4154639	261792	50.763
2	5.818	4029694	194831	49.237
total				100.000

2,4,6-Tribromo-5-hydroxy-*N*,*N*-diisopropyl-[1,1′-biphenyl]-3-carboxamide (2f).

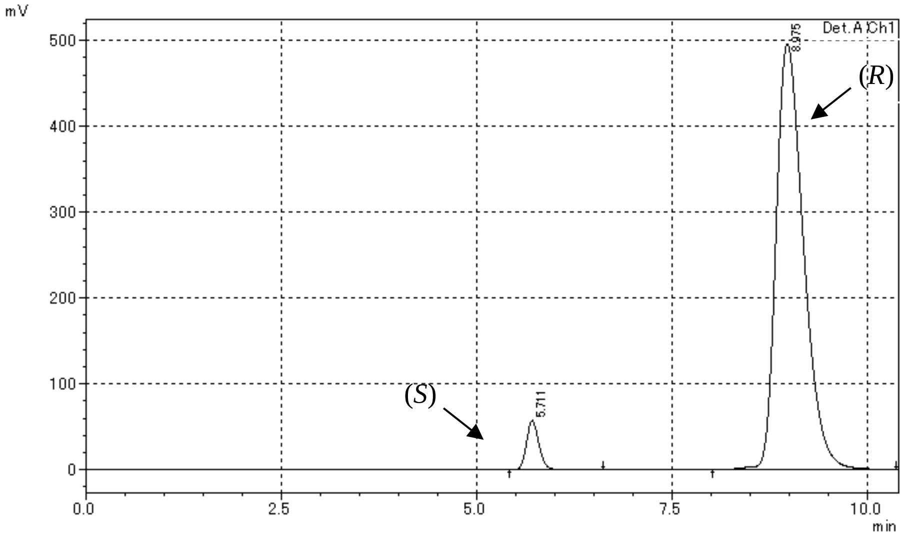

peak#	retention time (min)	area	height	area%
1	5.711	632162	57309	4.956
2	8.975	1212224	496056	95.044
total				100.000

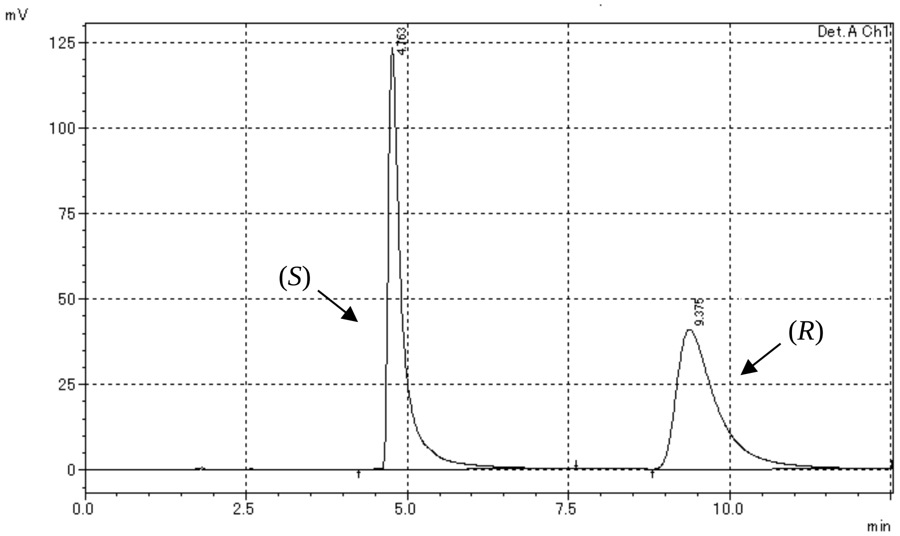

peak#	retention time (min)	area	height	area%
1	4.763	1872657	123423	51.222
2	9.375	1783317	40816	48.778
total				100.000

2,4,6-Tribromo-5-hydroxy-*N*,*N*-diisobutyl-4′-methoxy-[1,1′-biphenyl]-3-carboxamide (2g).

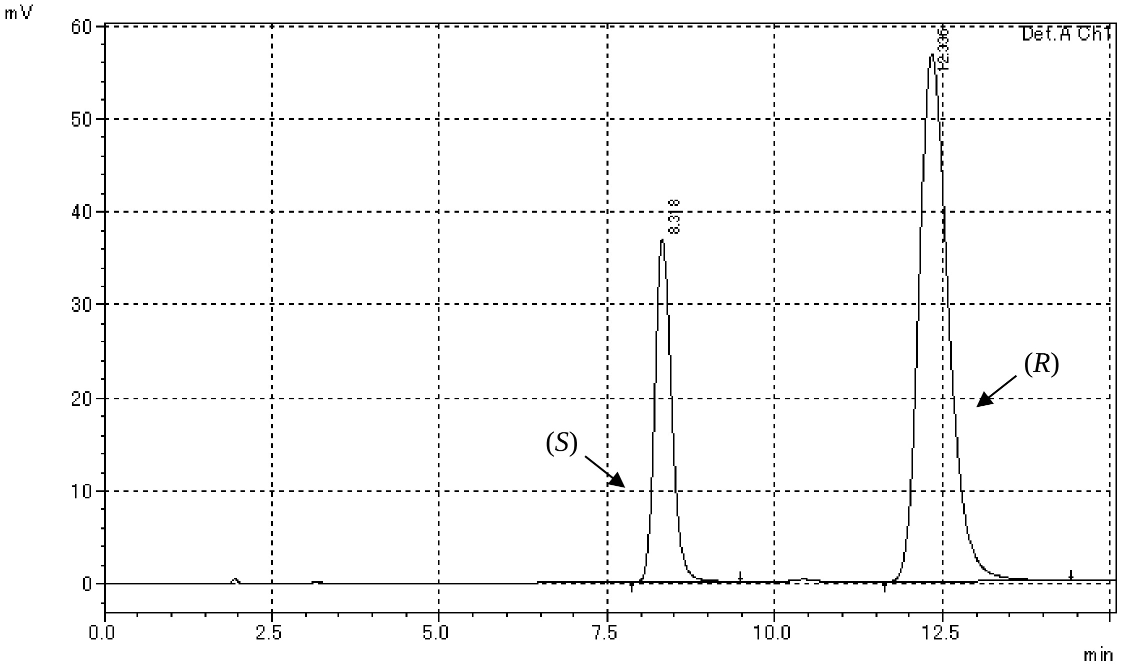

peak#	retention time (min)	area	height	area%
1	8.318	644593	36824	27.061
2	12.336	17373811	56731	72.939
total				100.000

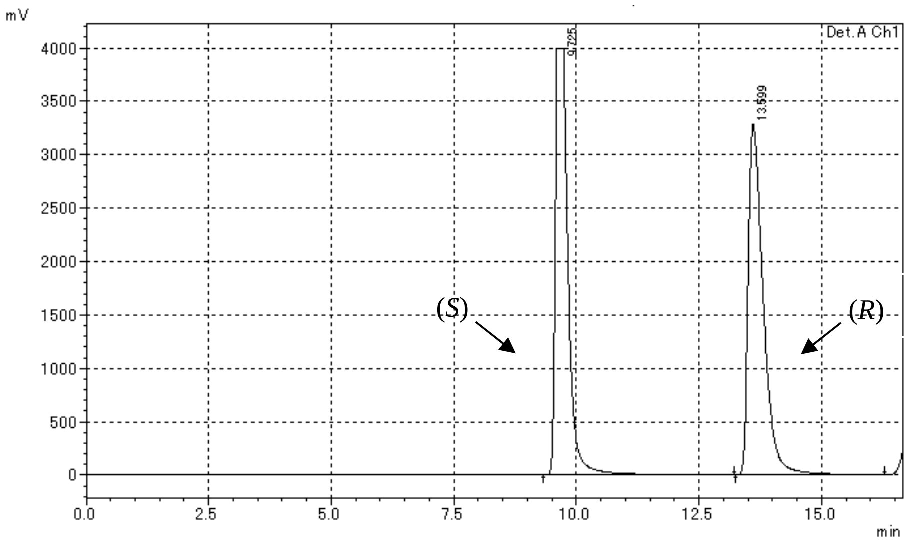

peak#	retention time (min)	area	height	area%
1	9.725	73326186	3999678	51.066
2	13.599	70264164	3281542	48.934
total				100.000

2,4,6-Tribromo-5-hydroxy-*N*,*N*-diisopropyl-4′-(trifluoromethyl)-[1,1′-biphenyl]-3-carboxamide (2h).

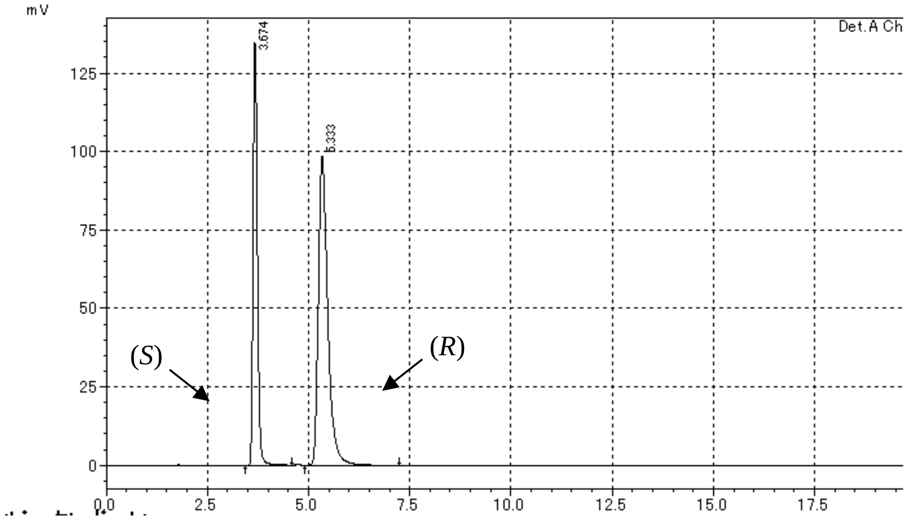

peak#	retention time (min)	area	height	area%
1	3.674	980406	134838	38.092
2	5.333	1593387	98473	61.908
total				100.000

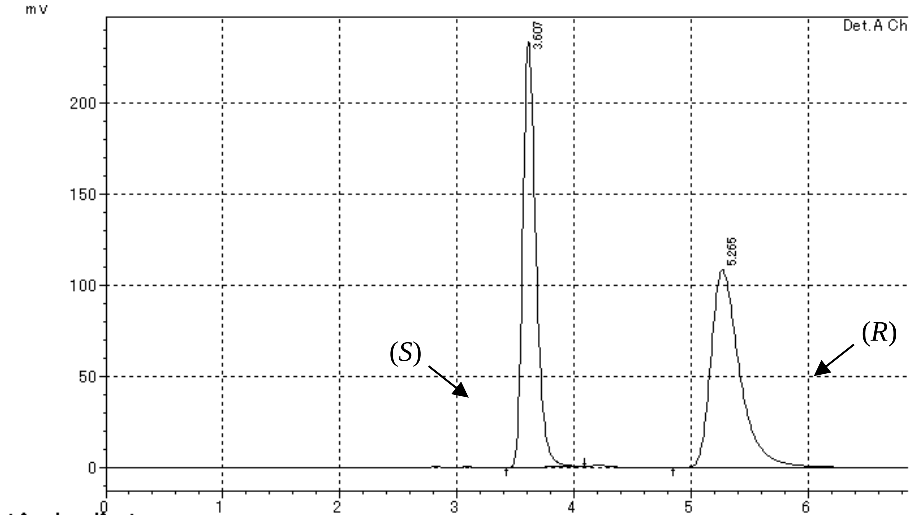

peak#	retention time (min)	area	height	area%
1	3.607	1829487	233634	49.709
2	5.265	1850888	108407	50.291
total				100.000

2,4,6-Tribromo-3-hydroxy-*N*,*N*-diisopropyl-5-(naphthalen-2-yl)benzamide (2i).

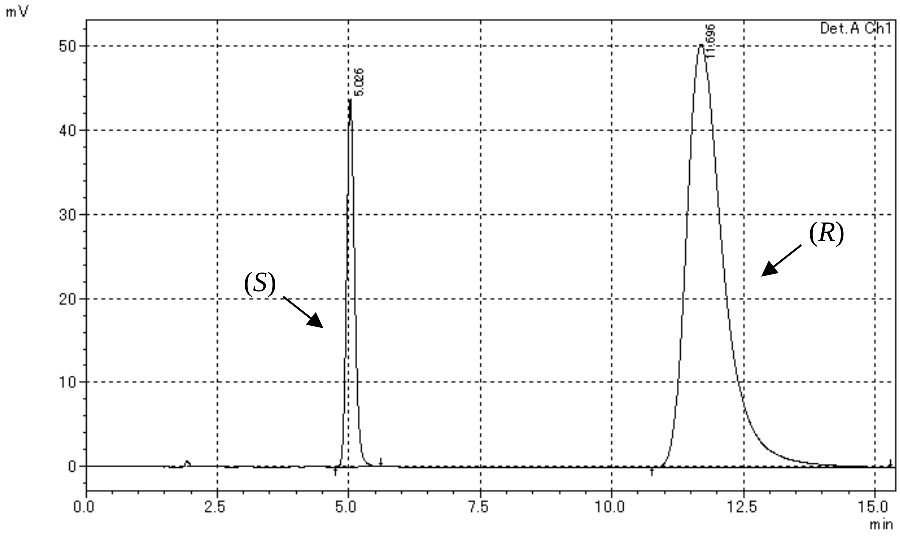

peak#	retention time (min)	area	height	area%
1	5.026	469616	43695	15.973
2	11.696	2470372	50387	84.027
total				100.000

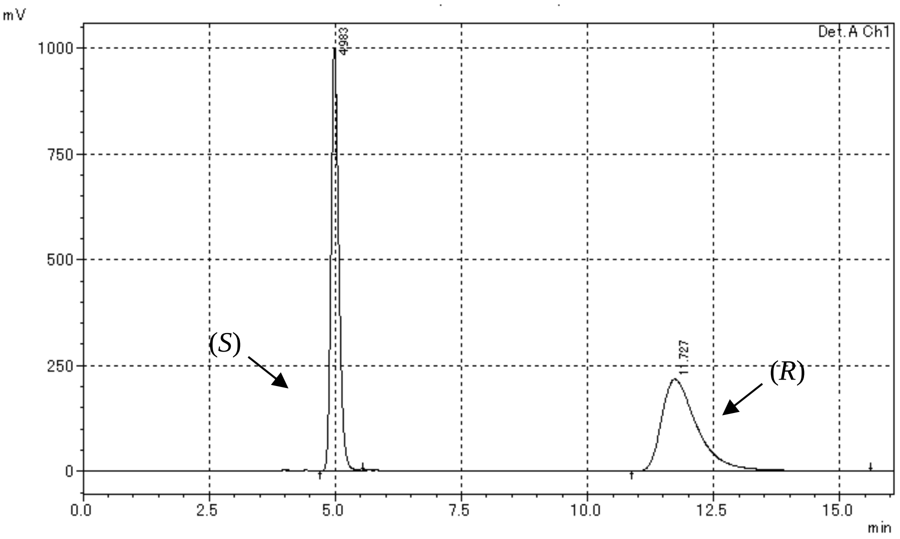

peak#	retention time (min)	area	height	area%
1	4.987	10753313	1000196	50.037
2	11.727	10737206	217719	49.963
total				100.000

2,4,6-Tribromo-3-cyclopropyl-5-hydroxy-*N*,*N*-diisopropylbenzamide (2j).

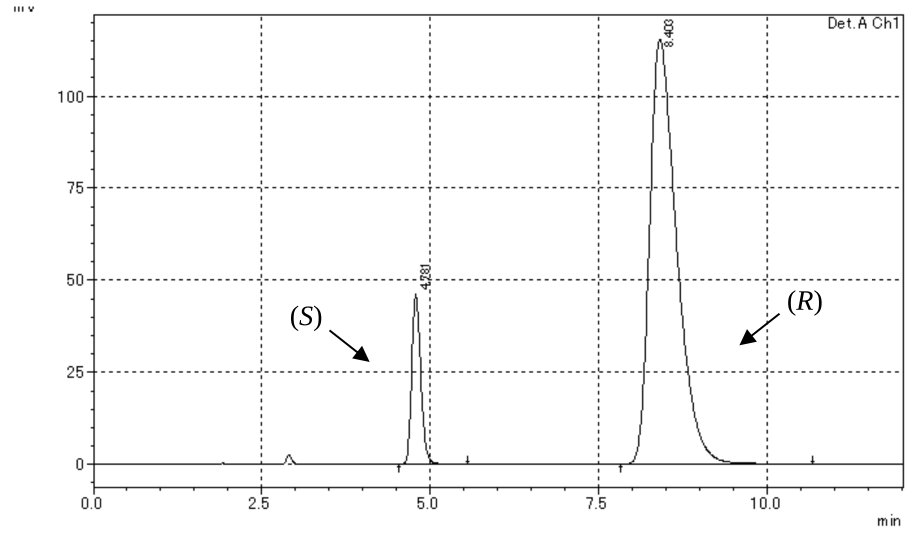

peak#	retention time (min)	area	height	area%
1	4.781	430854	46150	11.795
2	8.403	3221901	115353	88.205
total				100.000

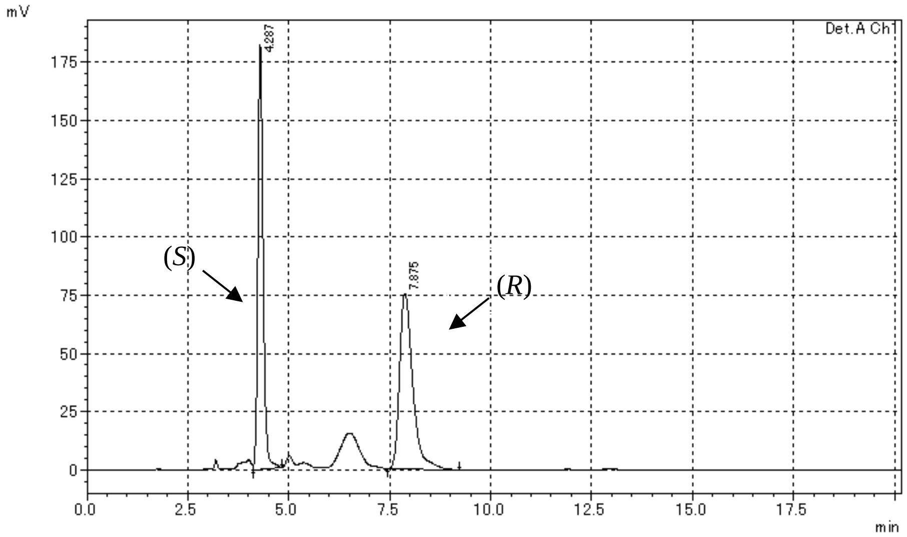

<ピークレポート>

検出器A Ch1 250nm

ピーク#	保持時間	面積	高さ	面積%	高さ%	マーク
1	4.287	1668862	182349	49.419	70.761	
2	7.875	1708114	75347	50.581	29.239	
合計		3376976	257696	100.000	100.000	

peak#	retention time (min)	area	height	area%
1	4.287	1668862	182349	49.419
2	7.875	1708114	75347	50.581
total				100.000

3,5-Dibromo-2-hydroxy-*N*,*N*-diisopropyl-[1,1′-biphenyl]-4-carboxamide (2k).

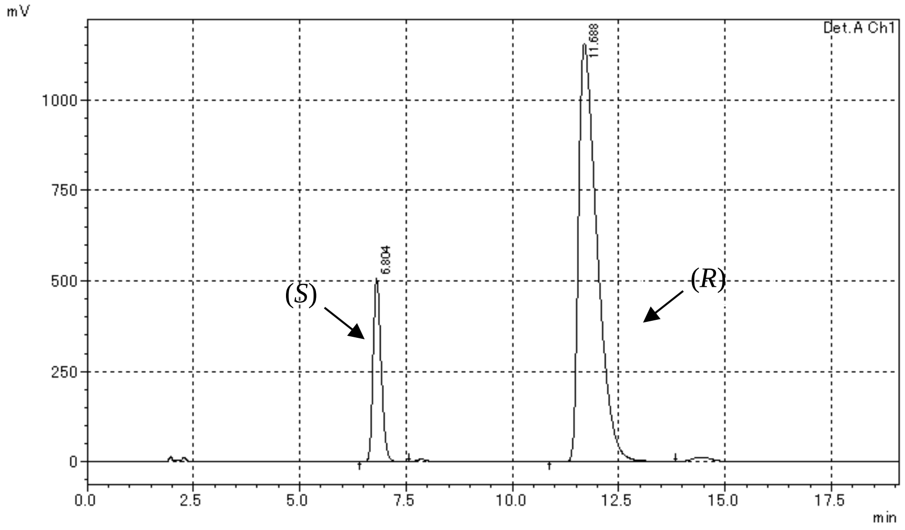

peak#	retention time (min)	area	height	area%
1	6.804	6658694	508131	15.960
2	11.688	35062429	1155409	84.040
total				100.000

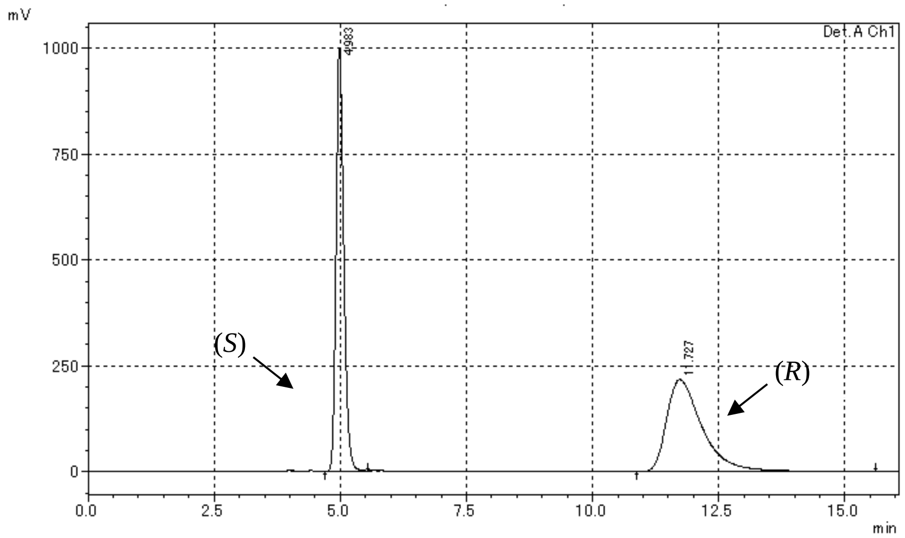

peak#	retention time (min)	area	height	area%
1	5.611	2404740	209452	49.378
2	9.096	2465312	97496	50.622
total				100.000

3,5-Dibromo-4-cyclopropyl-3-hydroxy-*N*,*N*-diisopropylbenzamide (2l).

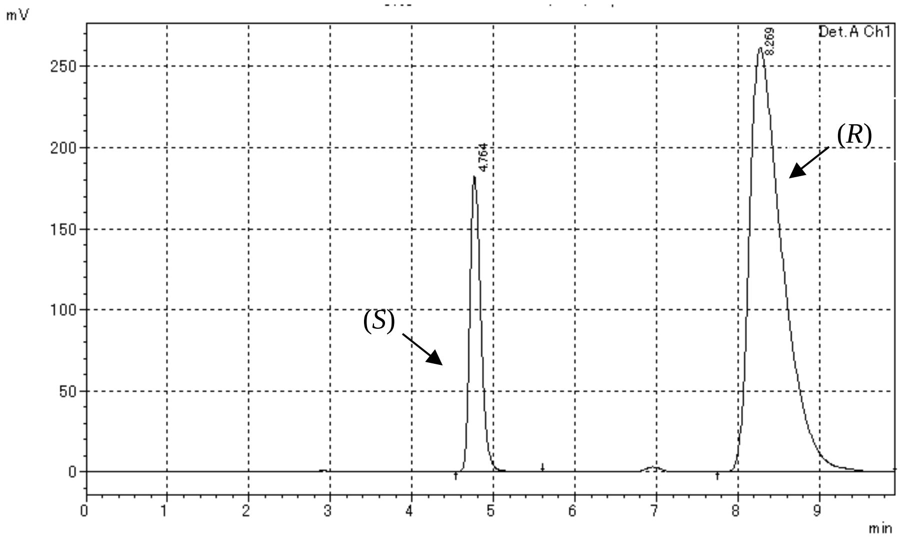

peak#	retention time (min)	area	height	area%
1	4.764	1698087	182482	18.846
2	8.269	7312347	261699	81.154
total				100.000

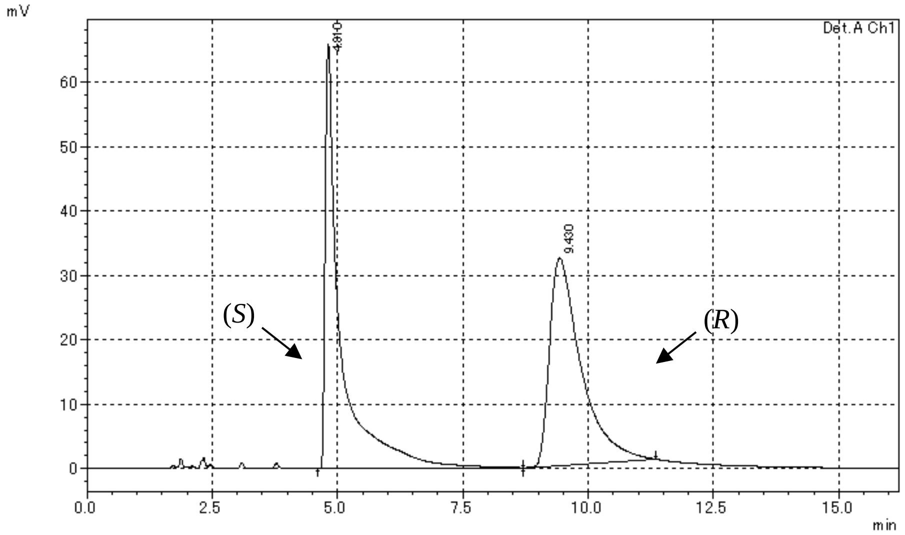

peak#	retention time (min)	area	height	area%
1	4.810	1392539	65889	49.754
2	9.430	1406282	32149	50.246
total				100.000

ORTEP Drawing of 2d

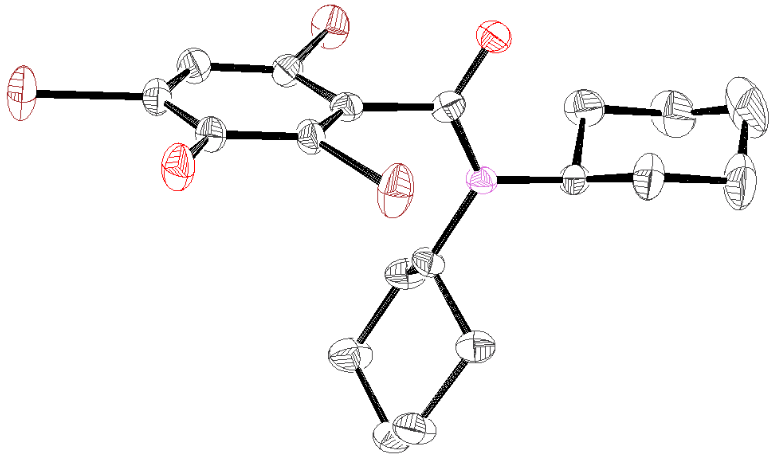

A. Crystal Data

Identification code	**2d**
Empirical Formula	$C_{19}H_{24}Br_3NO_2$
Formula Weight	538.12
Crystal Color, Habit	Colorless, Prism
Crystal Dimensions	0.760 × 0.560 × 0.550 mm
Crystal System	Orthorhombic
Lattice Type	Primitive
Lattice Parameters	a = 9.060(1) Å
	b = 14.023(2) Å
	c = 16.598(3) Å
	V = 2108.7(6) $Å^3$
Space Group	$P2_12_12_1$ (#19)
Z value	4
D_{calc}	1.695 g/cm^3
F_{000}	1064.00
μ(MoKα)	57.681 cm^{-1}

B. Intensity Measurements

Diffractometer	XtaLAB mini
Radiation	MoKα (λ = 0.71075 Å)

	Graphite monochromated
Voltage, Current	50 kV, 12 mA
Temperature	20.0 °C
Detector Aperture	75 mm (diameter)
Data Images	1080 exposures
ω Oscillation Range	–60.0–120.0°
Exposure Rate	48.0 sec./°
Detector Swing Angle	30.00°
ω Oscillation Range	–60.0–120.0°
Exposure Rate	48.0 sec./°
Detector Swing Angle	30.00°
ω Oscillation Range	–60.0–120.0°
Exposure Rate	48.0 sec./°
Detector Swing Angle	30.00°
ω Oscillation Range	–60.0–120.0°
Exposure Rate	48.0 sec./°
Detector Swing Angle	29.81°
ω Oscillation Range	–60.0–120.0°
Exposure Rate	48.0 sec./°
Detector Swing Angle	29.81°
ω Oscillation Range	–60.0–120.0°
Exposure Rate	48.0 sec./°
Detector Swing Angle	29.81°
Detector Position	49.89 mm
Pixel Size	0.146 mm
$2\theta_{max}$	55.0°
No. of Reflections Measured	Total: 21842
	Unique: 4825 (R_{int} = 0.0993)
	Friedel pairs: 2089
Corrections	Lorentz-polarization
	Absorption
	(trans. factors: 0.017–0.042)

C. Structure Solution and Refinement

Structure Solution	Direct Methods
Refinement	Full-matrix least-squares on F^2
Function Minimized	Σ w $(F_0^2 - F_c^2)^2$
Least Squares Weights	w = $1/[\sigma^2(F_0^2) + (0.1000 \cdot P)^2$ $+ 0.0000 \cdot P]$ where $P = (\mathrm{Max}(F_0^2,0) + 2F_c^2)/3$
$2\theta_{max}$ cutoff	55.0°
Anomalous Dispersion	All non-hydrogen atoms
No. Observations (All reflections)	4825
No. Variables	226
Reflection/Parameter Ratio	21.35
Residuals: R1 ($I>2.00\sigma(I)$)	0.0415
Residuals: R (All reflections)	0.0487
Residuals: wR2 (All reflections)	0.1129
Goodness of Fit Indicator	0.649
Flack Parameter (Friedel pairs = 2089)	–0.007(15)
Max Shift/Error in Final Cycle	0.001
Maximum peak in Final Diff. Map	1.19 $e^-/Å^3$
Minimum peak in Final Diff. Map	–0.83 $e^-/Å^3$

DFT Calculations of Rotational Barriers of 1a, 1m, 1o, 1p, 1q, and 2b

A. Reference.

Gaussian 09, Revision B.01,

Frisch, M. J.; Trucks, G. W.; Schlegel, H. B.; Scuseria, G. E.; Robb, M. A.; Cheeseman, J. R.; Scalmani, G.; Barone, V.; Mennucci, B.; Petersson, G. A.; Nakatsuji, H.; Caricato, M.; Li, X.; Hratchian, H. P.; Izmaylov, A. F. ; Bloino, J.; Zheng, G.; Sonnenberg, J. L.; Hada, M.; Ehara, M.; Toyota, K.; Fukuda, R.; Hasegawa, J.; Ishida, M.; Nakajima, T.; Honda, Y.; Kitao, O.; Nakai, H.; Vreven, T.; Montgomery, J. A. Jr.; Peralta, J. E.; Ogliaro, F.; Bearpark, M.; Heyd, J. J.; Brothers, E.; Kudin, K. N.; Staroverov, V. N.; Keith, T.; Kobayashi, R.; Normand, J.; Raghavachari, K.; Rendell, A.; Burant, J. C.; Iyengar, S. S.; Tomasi, J.; Cossi, M.; Rega, N.; Millam, J. M.; Klene, M.; Knox, J. E.; Cross, J. B.; Bakken, V.; Adamo, C.; Jaramillo, J.; Gomperts, R.; Stratmann, R. E.; Yazyev, O.; Austin, A. J.; Cammi, R.; Pomelli, C.; Ochterski, J. W.; Martin, R. L.; Morokuma, K.; Zakrzewski, V. G.; Voth, G. A.; Salvador, P.; Dannenberg, J. J.; Dapprich, S.; Daniels, A. D.; Farkas, O.; Foresman, J. B.; Ortiz, J. V.; Cioslowski, J.; Fox, D. J., Gaussian, Inc., Wallingford CT, 2010.

B. Details of energy diagrams for rotation of **1a**, **1m**, **1o**, **1p** and **1q**, including information on their input files.

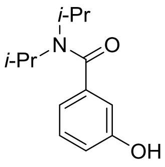

1a
7.6 kcal/mol

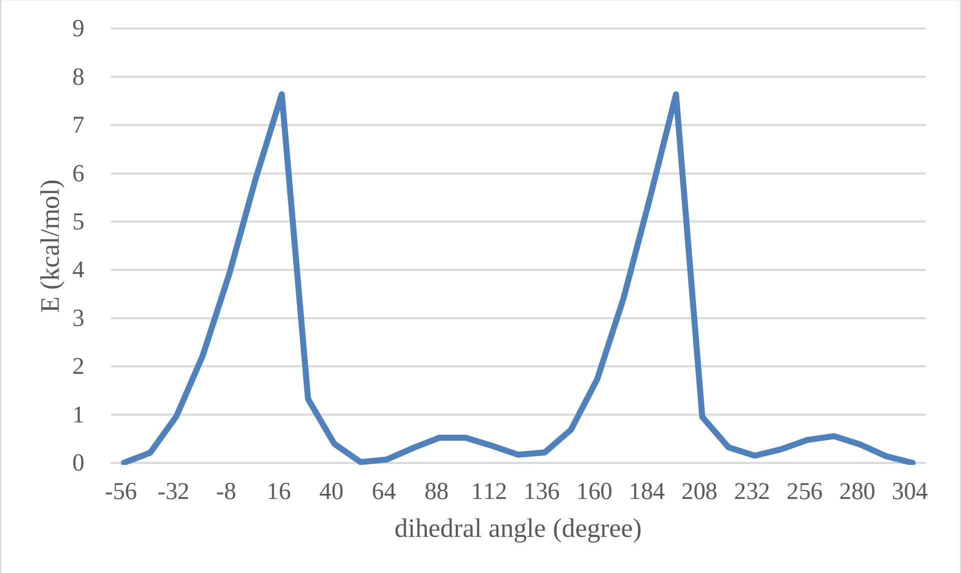

angle (degree)	energy (kcal/mol)	angle (degree)	energy (kcal/mol)
–55.9542	0	136.0457	0.2155
–43.9542	0.2115	148.0457	0.6848
–31.9542	0.9651	160.0457	1.7359
–19.9542	2.2204	172.0457	3.4092
–7.9542	3.9125	184.0457	5.4721
4.04573	5.8813	196.0457	7.6342
16.0457	7.6396	208.0457	0.9509
28.0457	1.3244	220.0457	0.3187
40.0457	0.3945	232.0457	0.1468
52.0457	0.0184	244.0457	0.2847
64.0457	0.0669	256.0457	0.4731
76.0457	0.3116	268.0457	0.5546
88.0457	0.5184	280.0457	0.3848
100.0457	0.5234	292.0457	0.1353
112.0457	0.3552	304.0457	0
124.0457	0.1718	$\Delta G^{\ddagger}$ =	7.63 kcal/mol

%nprocshared = 4
%mem = 16 GB
%chk = Amide2SCAN.chk

#p opt = modredundant b3lyp/6-31g(d) geom = connectivity

0 1

C	3.09796200	–1.43380300	0.99118200
C	3.78925800	–0.40712200	0.34474700
C	3.08952400	0.48747800	–0.47001900
C	1.70412500	0.36571900	–0.61903700
C	1.01618700	–0.65847600	0.03762000
C	1.72196800	–1.57350600	0.83213100
H	3.64590100	–2.13387700	1.61605400
H	4.86666000	–0.30715700	0.46502100
H	1.18809600	1.06756500	–1.26626900
H	1.18672500	–2.39095000	1.30327400
C	–0.45655900	–0.91942400	–0.19748200
O	–0.78446300	–2.04285400	–0.58244700
N	–1.36718300	0.09205700	–0.00586000
C	–2.78546200	–0.14230800	–0.39188300
H	–3.29334600	0.80005000	–0.16569600
C	–1.05523600	1.33501800	0.73578400
H	0.00831900	1.28486200	0.96900800
C	–1.80356200	1.41142000	2.07745200
H	–2.88837500	1.48904600	1.94491400
H	–1.59486700	0.52810000	2.68966400
H	–1.47588100	2.29739100	2.63329300
C	–1.28382400	2.59336600	–0.11588400
H	–2.34107700	2.73431900	–0.36759100
H	–0.95647300	3.48191300	0.43566600
H	–0.71712700	2.54541200	–1.05139700
C	–3.46935400	–1.24088700	0.43826300
H	–3.02686100	–2.21535200	0.22778700
H	–3.37821000	–1.03894100	1.51072300
H	–4.53725300	–1.27482100	0.19079700
C	–2.93002600	–0.38059000	–1.90215300
H	–3.99235500	–0.43748500	–2.16683600
H	–2.48165800	0.44216600	–2.46999800

H	–2.44687600	–1.31413000	–2.19645500
O	3.70175600	1.50675100	–1.14778500
H	4.65658800	1.46499900	–0.98340800

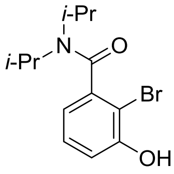

1m
19.0 kcal/mol

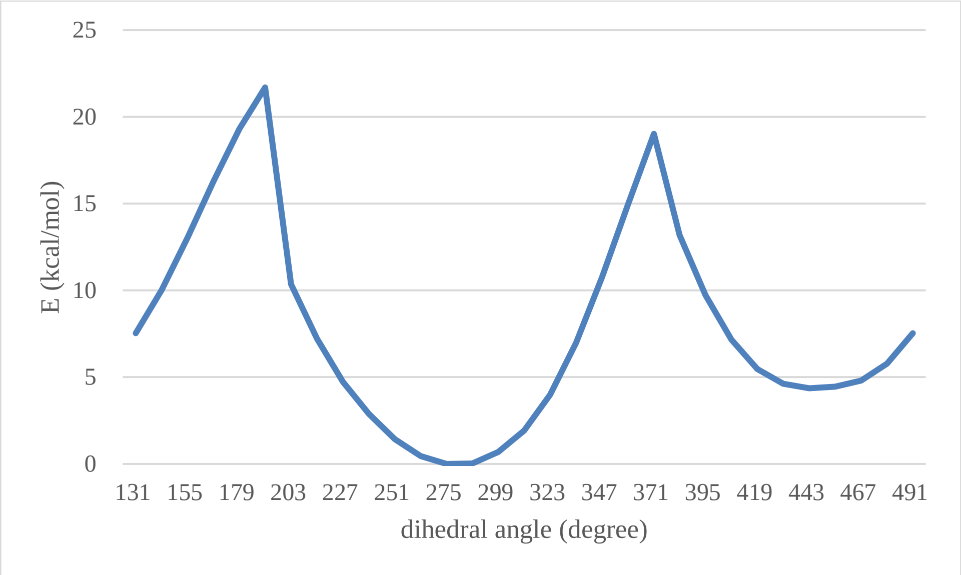

angle (degree)	energy (kcal/mol)	angle (degree)	energy (kcal/mol)
131.1454	7.532	323.1454	3.984
143.1454	10.027	335.1454	6.988
155.1454	13.048	347.1454	10.756
167.1454	16.296	359.1454	14.954
179.1454	19.311	371.1454	19.022
191.1454	21.693	383.1454	13.2
203.1454	10.357	395.1454	9.73
215.1454	7.226	407.1454	7.164
227.1454	4.732	419.1454	5.476
239.1454	2.883	431.1454	4.621
251.1454	1.43	443.1454	4.366
263.1454	0.452	455.1454	4.444
275.1454	0	467.1454	4.808
287.1454	0.027	479.1454	5.776
299.1454	0.684	491.1454	7.532
311.1454	1.929	$\Delta G^{\ddagger}$ =	19.02 kcal/mol

%nprocshared = 4
%mem = 1 GB
%chk = AMIDEBRSCAN.chk

#p opt = modredundant b3lyp/6-31g(d) geom = connectivity

0 1

C	0.94537100	2.79860100	–0.78459000
C	2.26025700	2.45375900	–0.55510700
C	2.59805800	1.14542500	–0.26627800
C	1.61521800	0.17199800	–0.21424000
C	0.29442000	0.51711500	–0.42699000
C	–0.03664000	1.83430800	–0.71292500
H	0.69084300	3.81262900	–1.01749000
H	3.02924800	3.20003700	–0.60108100
H	–1.06195700	2.07742400	–0.88551300
C	–0.82082400	–0.48902200	–0.51377400
N	–2.03133100	–0.09295700	0.04725800
O	–0.68007700	–1.52590900	–1.12784900
O	3.89255500	0.76861900	–0.02333600
H	4.52083500	1.49428800	–0.11031000
C	–2.06198200	0.18775700	1.52605200
H	–2.95420900	–0.29993100	1.88473400
C	–2.17055700	1.68428400	1.85289100
H	–2.36825200	1.81140600	2.91212400
H	–1.24853300	2.19449500	1.61286200
H	–2.97651000	2.14199200	1.29497000
C	–0.89085800	–0.45626500	2.29350300
H	–1.09484700	–0.39050300	3.35651900
H	–0.78224400	–1.50198400	2.03242800
H	0.04264900	0.05036800	2.10038800
C	–3.26702500	–0.70446900	–0.53404300
H	–3.34852600	–1.73830700	–0.21392900
C	–3.23337900	–0.65505600	–2.07572600
H	–4.18349900	–1.00421400	–2.46318200
H	–3.08740300	0.37050600	–2.39762700
H	–2.44742000	–1.27108400	–2.47589400
C	–4.51899600	0.07549900	–0.08314700
H	–4.43375200	1.10934200	–0.39436500
H	–5.38907200	–0.35686700	–0.56315000
H	–4.68860000	0.04504500	0.98356800
Br	2.17206700	–1.61197800	0.19399600

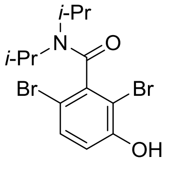

1o
27.3 kcal/mol

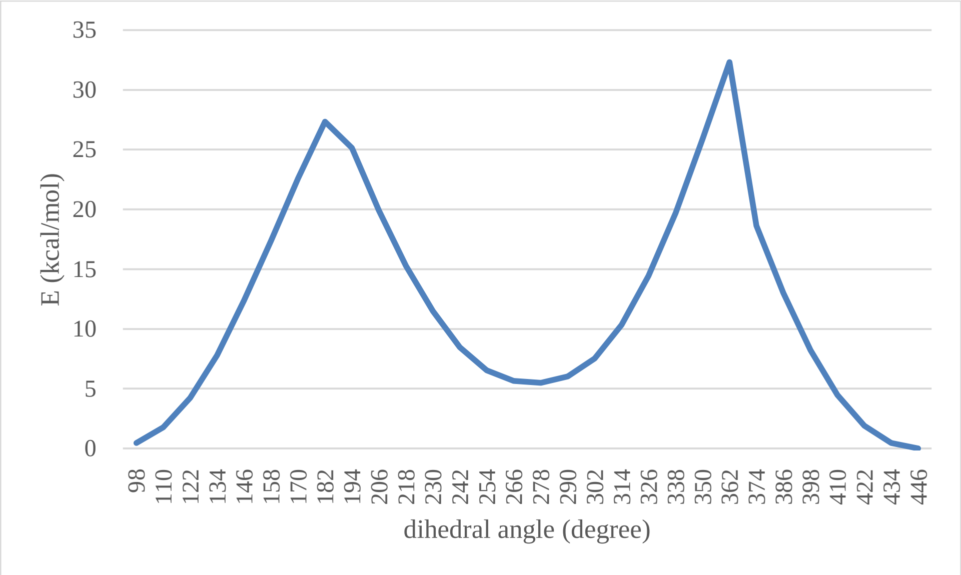

angle (degree)	energy (kcal/mol)	angle (degree)	energy (kcal/mol)
86.2195	0	278.2195	5.493
98.2195	0.452	290.2195	6.021
110.2195	1.784	302.2195	7.535
122.2195	4.242	314.2195	10.329
134.2195	7.796	326.2195	14.422
146.2195	12.382	338.2195	19.695
158.2195	17.395	350.2195	25.899
170.2195	22.594	362.2195	32.324
182.2195	27.342	374.2195	18.651
194.2195	25.144	386.2195	13.01
206.2195	19.93	398.2195	8.227
218.2195	15.259	410.2195	4.465
230.2195	11.492	422.2195	1.907
242.2195	8.455	434.2195	0.465
254.2195	6.528	446.2195	0
266.2195	5.647	$\Delta G^{\ddagger}$ =	27.34 kcal/mol

%nprocshared = 8
%mem = 30 GB
%chk = AMIDEBRBRrevrevrevSCAN.chk

#p opt = modredundant b3lyp/6-31g(d) geom = connectivity

0 1

C	–1.44396800	2.74381600	0.19188400
C	–2.71610800	2.19335800	0.31868300
C	–2.91380800	0.81734800	0.16666500
C	–1.80910600	0.00320200	–0.12267400
C	–0.52351500	0.53559600	–0.25814400
C	–0.36365200	1.91661800	–0.09652500
H	–1.29389600	3.81031500	0.31544000
H	–3.56426500	2.83766000	0.54054100
C	0.62946200	–0.33803900	–0.74117400
O	0.78450500	–0.43864600	–1.95443300
N	1.41182400	–0.95056900	0.19017400
Br	–2.08720000	–1.87195200	–0.31205800
Br	1.37567000	2.69913500	–0.24854800
O	–4.13445200	0.23178900	0.29120900
H	–4.79574500	0.91774200	0.47381900
C	2.52884600	–1.81820600	–0.27067100
H	2.99018100	–2.18912100	0.64929700
C	1.19956900	–0.79378900	1.64643500
H	0.34214800	–0.12649200	1.75138200
C	2.02671700	–3.03881300	–1.05670400
H	1.56981100	–2.73173800	–1.99910700
H	2.86851500	–3.70666900	–1.27430600
H	1.28574000	–3.59973900	–0.47738000
C	3.60144100	–1.02800600	–1.03538400
H	3.95286300	–0.17513500	–0.44520600
H	4.45930400	–1.67895600	–1.24112500
H	3.20819100	–0.65592800	–1.98300300
C	0.82101500	–2.12367300	2.31561100
H	1.63086400	–2.85997200	2.26037700
H	0.60048100	–1.95596300	3.37596600
H	–0.06929800	–2.54381800	1.83943600
C	2.39250500	–0.10369700	2.32510900
H	2.58136400	0.86640300	1.85669000
H	2.17151200	0.06037600	3.38589900
H	3.30621300	–0.70580000	2.26837700

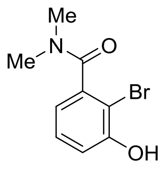

1p
14.2 kcal/mol

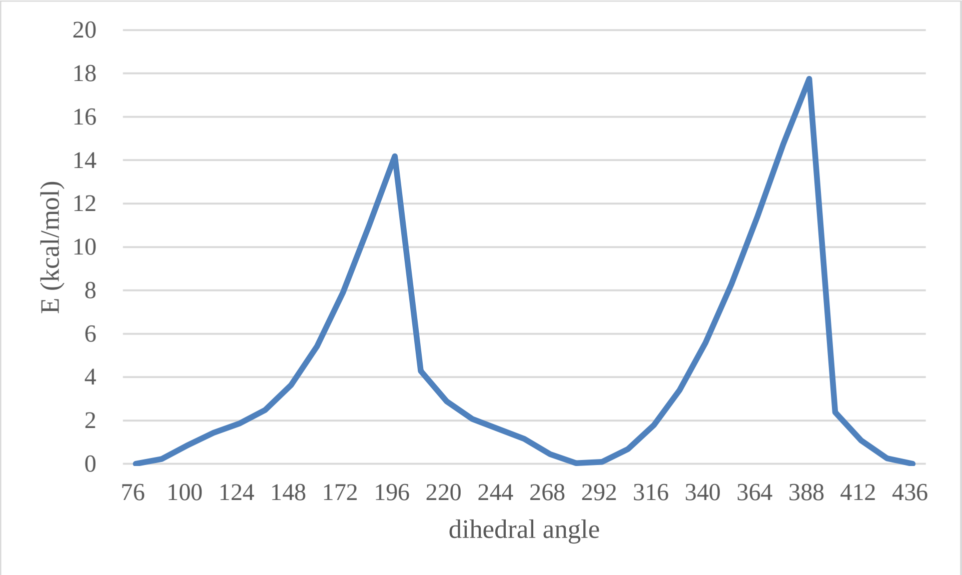

angle (degree)	energy (kcal/mol)	angle (degree)	energy (kcal/mol)
75.5281	0	267.5281	0.448
87.5281	0.229	279.5281	0.038
99.5281	0.86	291.5281	0.089
111.5281	1.433	303.5281	0.681
123.5281	1.865	315.5281	1.79
135.5281	2.483	327.5281	3.393
147.5281	3.63	339.5281	5.58
159.5281	5.417	351.5281	8.282
171.5281	7.899	363.5281	11.398
183.5281	10.964	375.5281	14.737
195.5281	14.184	387.5281	17.752
207.5281	4.284	399.5281	2.383
219.5281	2.884	411.5281	1.082
231.5281	2.071	423.5281	0.264
243.5281	1.608	435.5281	0
255.5281	1.156	$\Delta G^{\ddagger}$ =	14.18 kcal/mol

%nprocshared = 8
%mem = 30 GB
%chk = MeAmideBr2SCAN.chk

#p opt = modredundant b3lyp/6-31g(d) geom = connectivity

0 1

C	2.23978600	0.18571300	0.13824000
C	2.70693800	1.49875000	0.25356800
C	1.82388200	2.57145900	0.14280700
C	0.46910200	2.34520100	–0.08080800
C	–0.02161500	1.03605600	–0.17678500
C	0.87048700	–0.03422200	–0.07685100
H	3.76776700	1.67372000	0.42313600
H	2.20085000	3.58667600	0.22510700
H	–0.22068800	3.17526900	–0.19325800
C	–1.48613600	0.85412200	–0.52306800
O	–1.87414700	1.18569300	–1.63906300
N	–2.32168000	0.37151500	0.44813600
C	–3.71458200	0.11722000	0.11028200
H	–3.94057200	–0.95419800	0.19496900
H	–4.37765100	0.66626000	0.79092400
H	–3.88660400	0.44616700	–0.91359300
C	–1.91556600	–0.00168900	1.79236400
H	–2.60301700	0.44732500	2.52023700
H	–1.93145800	–1.09133500	1.92364900
H	–0.90880900	0.35815900	2.00193200
O	3.06119900	–0.89575600	0.22868600
H	3.97229700	–0.58656300	0.35261400
Br	0.25284200	–1.83077800	–0.24737500

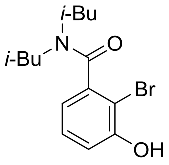

1q
18.0 kcal/mol

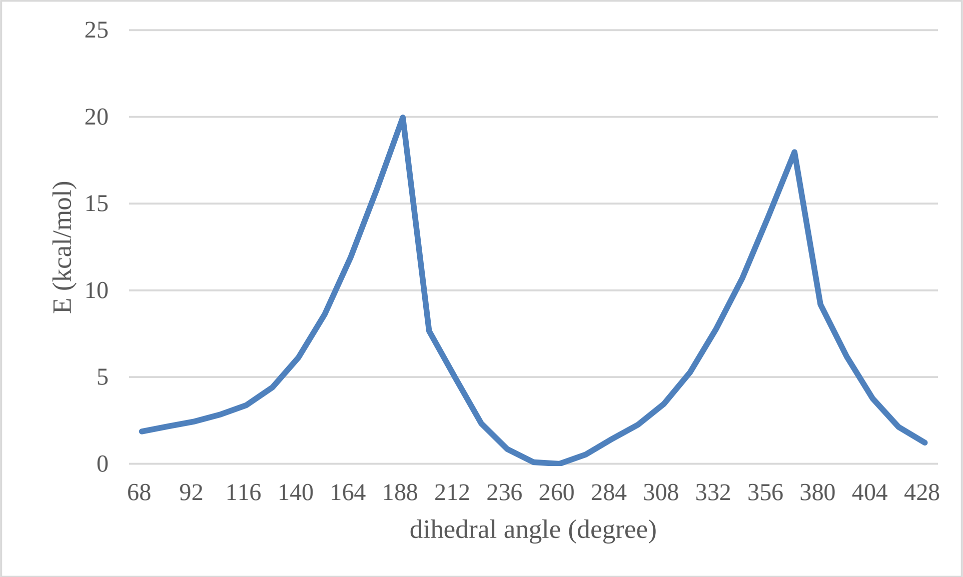

angle (degree)	energy (kcal/mol)	angle (degree)	energy (kcal/mol)
68.2176	1.87	260.2176	0
80.2176	2.159	272.2176	0.549
92.2176	2.447	284.2176	1.42
104.2176	2.85	296.2176	2.266
116.2176	3.384	308.2176	3.465
128.2176	4.412	320.2176	5.28
140.2176	6.129	332.2176	7.763
152.2176	8.618	344.2176	10.713
164.2176	11.895	356.2176	14.236
176.2176	15.836	368.2176	17.963
188.2176	19.969	380.2176	9.187
200.2176	7.663	392.2176	6.179
212.2176	4.981	404.2176	3.777
224.2176	2.331	416.2176	2.125
236.2176	0.849	428.2176	1.232
248.2176	0.096	$\Delta G^{\ddagger}$ =	17.96 kcal/mol

%nprocshared = 8
%mem = 30 GB
%chk = iBuAmideBrSCAN.chk

#p opt = modredundant b3lyp/6-31g(d) geom = connectivity

0 1

C	3.50087200	–0.69635700	2.16773000
C	2.29675400	–0.81132900	2.85826700
C	1.08819700	–0.78487600	2.16715800
C	1.07224600	–0.64591500	0.77187400
C	2.28434800	–0.54823900	0.08327700
C	3.50538500	–0.56231500	0.77507500
H	4.44756500	–0.71360700	2.70457600
H	2.30550800	–0.92403400	3.93848500
H	0.14825200	–0.88383000	2.70203100
C	–0.23980600	–0.77149000	0.02319300
O	–0.46732800	–1.80305600	–0.60297800
N	–1.14722800	0.25318400	0.09799200
C	–2.44917000	0.01885900	–0.54566400
H	–2.87724300	0.99559200	–0.78584300
H	–2.26744900	–0.51102900	–1.48405800
C	–0.98906800	1.49694900	0.85772800
H	–1.86567000	1.60055000	1.51307800
H	–0.11779100	1.40252800	1.50936900
C	–0.84270500	2.77626200	0.00380800
H	–1.64841200	2.78160700	–0.74400500
C	–1.02911200	4.00977000	0.90040300
H	–0.26789500	4.04029100	1.69109100
H	–0.93547300	4.93333800	0.31836900
H	–2.01377100	4.01595200	1.38359600
C	0.49909200	2.82937300	–0.73552200
H	0.67838000	1.93132900	–1.33294800
H	0.53736400	3.69510600	–1.40694000
H	1.33086200	2.92381700	–0.02496900
C	–3.40359400	–0.80163800	0.33506600
H	–3.57946200	–0.27161800	1.28357000
H	–2.89804500	–1.74228400	0.58143300
C	–4.75929900	–1.11941800	–0.32618000
H	–4.55070800	–1.60114700	–1.29373600
C	–5.54876700	–2.11911800	0.53220600
H	–5.78354600	–1.69199600	1.51626100

H	–6.49764300	–2.38961100	0.05392100
H	–4.97910200	–3.04120700	0.69582000
C	–5.59885400	0.13989800	–0.59448900
H	–5.80542500	0.67804500	0.34044900
H	–5.09962500	0.83605200	–1.27765500
H	–6.56367900	–0.12242000	–1.04435100
O	4.65532500	–0.44523800	0.05711400
H	5.40954900	–0.49210500	0.66540700
Br	2.31717000	–0.39136400	–1.81330200

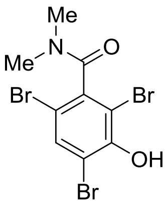

2b
22.9 kcal/mol

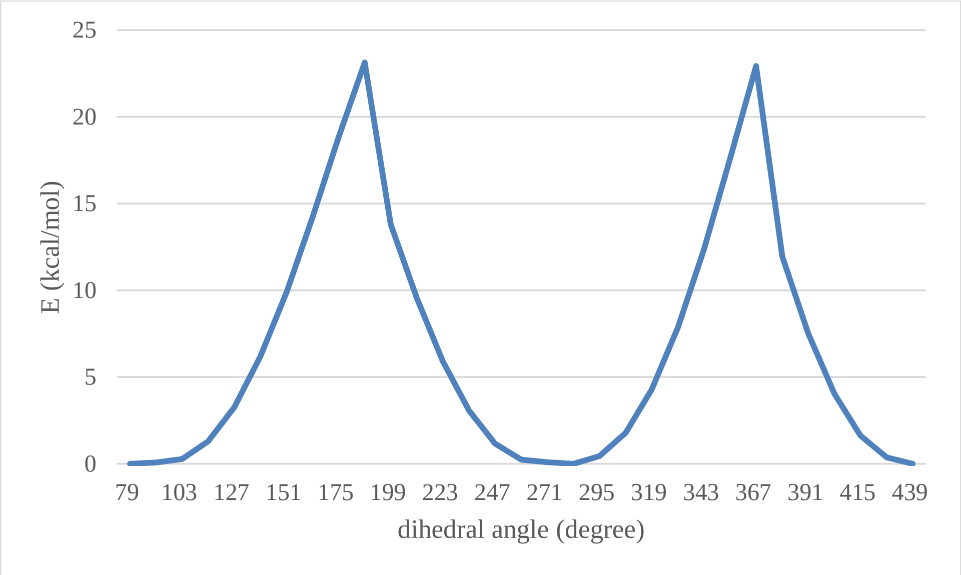

angle (degree)	energy (kcal/mol)	angle (degree)	energy (kcal/mol)
78.5121	0	270.5119	0.1036
90.5121	0.091	282.5119	0.0023
102.512	0.2912	294.5119	0.4431
114.5119	1.2979	306.5119	1.7949
126.5119	3.2791	318.5119	4.2895
138.5119	6.1924	330.5119	7.8576
150.512	9.8914	342.5119	12.354
162.512	14.2054	354.5119	17.5996
174.5119	18.8082	366.5119	22.9257
186.5119	23.1391	378.5119	11.9367
198.5119	13.82	390.5119	7.5159
210.5119	9.5484	402.5119	4.0308
222.5119	5.9113	414.5119	1.6377
234.5119	3.0711	426.5119	0.3722
246.5119	1.1754	438.5119	0
258.5119	0.2454	$\Delta G^{\ddagger}$ =	22.92 kcal/

%nprocshared = 4
%mem = 1 GB
%chk=AMIDE_2b_scan.chk

#p opt = modredundant b3lyp/6-31g(d) geom = connectivity

0 1

C	–2.05676	–0.24088	0.01737
C	–1.31596	–1.41599	–0.00423
C	0.0682	–1.33606	–0.11403
C	0.72343	–0.10201	–0.19609
C	–0.05166	1.05968	–0.15901
C	–1.45117	1.02214	–0.05434
H	–1.80994	–2.37719	0.0631
C	2.21648	–0.03058	–0.47757
O	2.59976	–0.18157	–1.63022
N	3.04628	0.18457	0.58404
C	4.47188	0.34419	0.33407
H	4.78195	1.38226	0.5151
H	5.04472	–0.31343	0.99852
H	4.67194	0.08411	–0.70448
C	2.59673	0.43955	1.9422
H	3.21389	–0.13208	2.64476
H	2.67508	1.50586	2.19321
H	1.56006	0.12713	2.06643
O	–2.13805	2.18269	–0.02283
H	–3.0896	1.97674	0.03974
Br	0.79483	2.7623	–0.22725
Br	1.07497	–2.95562	–0.11557
Br	–3.9633	–0.32081	0.15967
